# A high-resolution map of non-crossover events reveals impacts of genetic diversity on mammalian meiotic recombination

**DOI:** 10.1038/s41467-019-11675-y

**Published:** 2019-08-29

**Authors:** Ran Li, Emmanuelle Bitoun, Nicolas Altemose, Robert W. Davies, Benjamin Davies, Simon R. Myers

**Affiliations:** 1 The Wellcome Centre for Human Genetics, Roosevelt Drive, University of Oxford, Oxford, OX3 7BN UK; 2 Department of Statistics, University of Oxford, 24-29 St Giles’, Oxford, OX1 3LB UK; 3 Present Address: Target Discovery Institute, NDM Research Building, University of Oxford, Old Road Campus, Headington, Oxford, OX3 7FZ UK; 4 Present Address: Department of Bioengineering, Stanley Hall, University of California, Berkeley, CA 94720 USA

**Keywords:** DNA mismatch repair, Homologous recombination, DNA recombination, Genetic hybridization, Genetic variation

## Abstract

During meiotic recombination, homologue-templated repair of programmed DNA double-strand breaks (DSBs) produces relatively few crossovers and many difficult-to-detect non-crossovers. By intercrossing two diverged mouse subspecies over five generations and deep-sequencing 119 offspring, we detect thousands of crossover and non-crossover events genome-wide with unprecedented power and spatial resolution. We find that both crossovers and non-crossovers are strongly depleted at DSB hotspots where the DSB-positioning protein PRDM9 fails to bind to the unbroken homologous chromosome, revealing that PRDM9 also functions to promote homologue-templated repair. Our results show that complex non-crossovers are much rarer in mice than humans, consistent with complex events arising from accumulated non-programmed DNA damage. Unexpectedly, we also find that GC-biased gene conversion is restricted to non-crossover tracts containing only one mismatch. These results demonstrate that local genetic diversity profoundly alters meiotic repair pathway decisions via at least two distinct mechanisms, impacting genome evolution and *Prdm9*-related hybrid infertility.

## Abstract (web summary)

During meiotic recombination, genetic information is transferred or exchanged between parental chromosome copies. Using a large hybrid mouse pedigree, the authors generated high-resolution maps of these transfer/exchange events and discovered new properties governing their processing and resolution.

## Introduction

During meiosis, genetic information is exchanged between homologous chromosomes via the process of recombination. In mammals and other taxa, recombination is essential for the proper pairing of homologous chromosomes (synapsis) and their segregation into gametes and, together with mutation, generates all genetic variation^1,2^. In many species, most recombination events cluster into small 1–2 kb regions of the genome, called recombination hotspots. In mice, humans and likely many other vertebrate species^3^, these hotspots are positioned mainly by PRDM9^4–9^, a zinc-finger protein that binds specific DNA sequence motifs and deposits at least two histone modifications, H3K4me3 and H3K36me3, on the surrounding nucleosomes^10,11^. DNA double-strand breaks (DSBs) subsequently form near a small subset of PRDM9 binding sites in each cell^12^, and DSB processing results in single-stranded DNA decorated with the strand exchange proteins RAD51 and DMC1^8^. Meiotic recombination hotspots can be mapped genome-wide by performing versions of Chromatin ImmunoPrecipitation with high-throughput sequencing (ChIP-seq) in bulk gonadal tissue samples^13–15^. Because H3K4me3 is deposited at essentially all PRDM9 binding sites, meiosis-specific H3K4me3 ChIP-seq enrichment can be used to approximate the frequency of PRDM9 binding at each site^16,17^. DMC1 marks DSB sites prior to repair processing^8^, so DMC1 enrichment at individual hotspots increases both with the rate at which DSBs occur and with the average time until these DSBs are repaired.

**Fig. 1:**
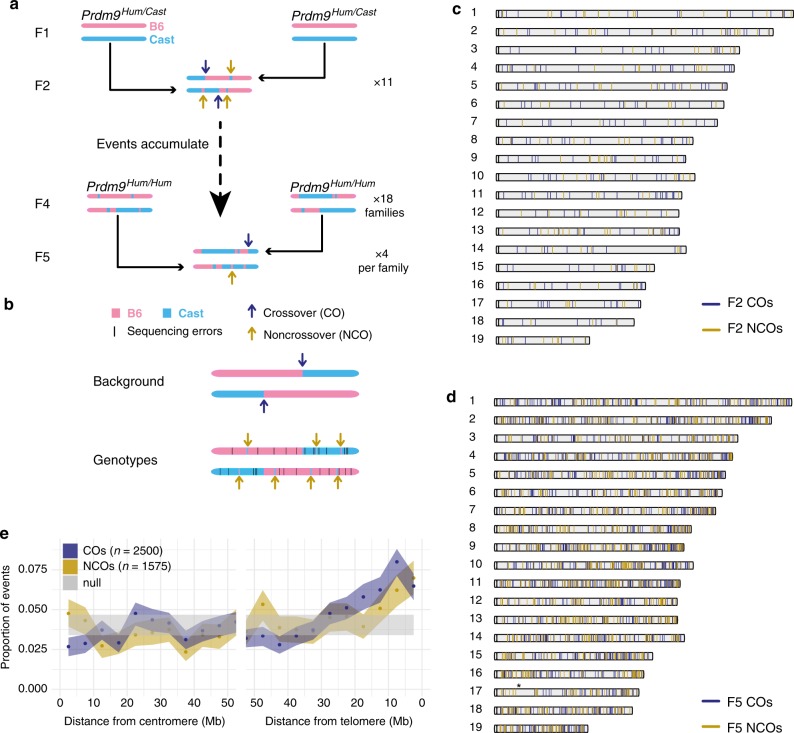
Study design and properties of CO and NCO events. **a** Study design. Arrows indicate locations of de novo CO and NCO events. 11 F2 mice, 36 F4 mice, and 72 F5 mice were sequenced. **b** Detection of NCOs by comparing observed genotypes and background. **c**, **d** Distribution of identified CO breakpoints and NCOs across autosomes from F2 (**c**) and F5 (**d**) animals. **d** Includes both de novo and distinct inherited events from the F5 mice. Asterisk on Chr17 indicates the position of the *Prdm9* gene, which was selected to be homozygous and may show lower power to detect nearby F5 events. **e** Binning all events by their distance to the centromere or telomere (*x*-axis); shaded regions represent 90% bootstrap CIs. “Null” represents the results of scrambling NCO positions uniformly across each chromosome

Each DSB can ultimately repair by homologous recombination in several ways. First, the single-stranded ends surrounding each DSB must participate in homology search, a still largely unresolved molecular process in meiosis, to identify a suitable repair template within the vast search space of the genome. The homologue is favoured as a repair template, as opposed to the sister chromatid^18^. One exception is the X chromosome, which has no homologue in males and is thought to rely on sister-templated repair later in male meiotic prophase^18^. A minority of DSBs form crossovers (COs), involving reciprocal exchanges between homologues, while many more DSBs become non-crossovers (NCOs), in which a section of genetic material is copied (converted) from the homologue, without the donating chromosome being altered^19^ (Fig. 1a).

Although the role of recombination in shaping genetic variation is well understood, our understanding of possible effects in the reverse direction—of local genetic variation on NCO and CO event outcomes—remains very incomplete in mammals. Previous work^20–22^ has revealed impacts of genetic variation on the formation of DSBs, by altering PRDM9 binding properties. We previously published evidence that the degree to which PRDM9 binding is symmetric—that is, whether PRDM9 binds both homologues equally well at each site—predicts increased synapsis and fertility in certain hybrid male mice^23^. Moreover, individual asymmetric hotspots show elevated DMC1 ChIP-seq enrichment relative to H3K4me3, most consistent with the possibility that these DSBs take longer to repair^23^. One study in mice reported that hotspots with high polymorphism rates, particularly those with asymmetry, show fewer COs than expected from their DMC1 enrichment in males^22^. However, their data could not discriminate between the following causal possibilities: (i) genetic diversity *per se* was responsible (similar to findings in yeast^24^); (ii) DMC1 elevation through repair delay entirely drove the signal; or (iii) these sites were preferentially repaired instead by NCO recombination. Despite this important progress, it therefore remains unclear how, or even if, local genetic variation might impact eventual recombination outcomes following DSB formation, or how this might differ for COs and NCOs and between sexes.

Furthermore, many fundamental questions remain about the process of NCO recombination itself due to the difficulty of detecting NCO events, as NCOs are very short^25^ and the ability to detect them relies on the conversion of nearby SNPs, which is less likely to occur in individuals with low heterozygosity. Previous studies in humans^26,27^ have revealed that in males, most (~70%) NCO events occur within PRDM9-positioned recombination hotspots and are predominantly short (<1 kb) and simple: they comprise contiguous tracts of converted SNPs. In contrast, complex NCO events, which contain both converted and non-converted SNPs and often extend over 1 kb, are seen at a greater rate in females than males, and they show an association with maternal age^27^. Finally, human NCO events show a strong overall bias towards G/C bases (68%)^26–28^, as opposed to A/T bases^29–31^, occurring via an unresolved molecular mechanism. This phenomenon is thought to have driven regional differences in the GC-content of many species genome-wide^32–34^. Possible causes of this GC-bias include either subtle event initiation biases^33,35^, or heteroduplex DNA repair pathways^36^. It has been unclear to what extent these findings for humans might generalise to other species, or to what extent findings from individual hotspots^25,28,37^ might generalise to hotspots genome-wide. Importantly, lack of power has prevented resolution thus far of basic questions about meiotic recombination, including any precise estimate of the length of underlying NCO tracts^25–27,38^, the total number of homologous recombination events per meiosis, or where NCO events position relative to PRDM9 binding sites and DSBs, although studies at individual hotspots in mice have suggested a fairly broad distribution^25,39^.

To investigate links between genetic variation and repair outcomes, we mapped both CO and NCO events in mice, including mice humanised at *Prdm9*^23^, in both sexes. Critically, we also gathered complementary H3K4me3 and DMC1 ChIP-seq data (DMC1 data generated elsewhere^40^) in the male parental, or closely related, animals^17,20,22,23,27,40^, allowing us to analyse PRDM9 binding and DSB formation. Together, these data provide an unprecedented opportunity to investigate each step of meiotic recombination genome-wide and with high resolution, from PRDM9 binding, to DSB formation, to CO and NCO repair.

## Results

### Building a high-resolution CO and NCO map

We identified both CO and NCO events in hybrids of two mouse strains: C57BL/6J, humanised at *Prdm9* (hereafter B6^*Hum*^, and predominantly of *Mus musculus domesticus* origin) and CAST/EiJ (hereafter CAST, predominantly of *Mus musculus castaneus* origin). Their high sequence divergence (0.7%; Methods) improves power to detect NCO events in offspring. B6^*Hum*^ is identical to C57BL/6J except that the portion of the B6 *Prdm9* exon 10 encoding the DNA-binding zinc-finger array has been replaced with the orthologous sequence from the human *PRDM9 B* allele^23^, to produce a new allele we label *Prdm9*^*Hum*^, distinct from the *Prdm9*^*Cast*^ allele possessed by CAST. The different *Prdm9* alleles allow us to distinguish the properties of *Prdm9*^*Cast*^ and *Prdm9*^*Hum*^ controlled recombination hotspots, with the humanised allele being of interest because it has not co-evolved with either mouse subspecies’ genome. We sequenced 11 F2 offspring of (B6xCAST)F1 mice, and after breeding for five generations in total to accumulate recombination events controlled by *Prdm9*^*Hum*^, we sequenced 72 (B6xCAST)F5-*Prdm9*^*Hum/Hum*^ mice and their 36 F4 parents (Methods; Fig. 1a; Supplementary Fig. 1; Supplementary Data 1). We also gathered ChIP-seq data for both DMC1^40^ and H3K4me3 in testes from male (B6xCAST)F1-*Prdm9*^*Hum/Cast*^ mice, allowing us to compare these to NCO/CO event outcomes. Importantly, the high heterozygosity in these mice enabled us to assign ChIP-seq reads to each homologue separately, providing a measure of homologue-specific PRDM9 binding and DSB formation. 23,748 DMC1 peaks correspond to DSB hotspots, and 63,050 PRDM9-dependent H3K4me3 peaks mark PRDM9 binding sites. Essentially all DMC1 peaks show evidence of H3K4me3 enrichment (98% at *p* < 0.05 by likelihood ratio testing; Supplementary Data 2). For most peaks, we are able to determine which *Prdm9* allele controls them (Supplementary Note 1). Together these data allow us to compare signatures of PRDM9 binding, DSB formation, and NCO/CO events in both sexes.

To find both CO and NCO events, we developed and applied a Hidden Markov Model (HMM)-based algorithm to infer background states (B6/B6, B6/CAST and CAST/CAST) across the genome in each mouse to test potential gene conversions against (Fig. 1b and Supplementary Note 2). Each SNP is assigned a particular background state. CO events correspond to background changes between successive SNPs. SNPs with genotypes not matching their local background represent either rare NCO events, or common sequencing errors. Following careful filtering to exclude such errors (Supplementary Note 3 and Supplementary Table 1) we identified 183 NCOs and 295 CO events on autosomes from the 11 F2 animals (Fig. 1c) and 1,392 NCOs and 2,205 COs in the 72 F5 mice (Fig. 1d and Supplementary Data 3 and 4). This represents ~3-fold more NCO events identified by direct sequencing, which avoids ascertainment biases, than in the largest previous mammalian study^27^, which was performed in humans - allowing inter-species comparisons. Sequencing-based validation of F2 events (Methods) estimated that 91% of the identified NCO events are real, while using simulations we estimate our power to identify those NCO events containing at least 1 SNP as 73% or above (Methods and Supplementary Fig. 2a, b). In the F5 mice, we were able to identify both de novo and parentally inherited NCO and CO events; and we were able to assign a subset to the maternal or paternal meiosis (Methods). Among the 821 F5 de novo COs, we identified 321 paternal events and 382 maternal events (along with 118 events that were unable to be assigned). NCO events can be assigned to a genetic background by determining whether they result from a DSB on the B6 or CAST chromosome. In the 510 de novo NCOs from F5 mice, we identified 121 paternal events and 130 maternal events (with 259 unassigned). We note that our CO breakpoints are resolved to the sequence level, with a median CO breakpoint resolution of 396 bp.

### Overall event properties

NCO and CO events, as well as DMC1 and H3K4me3, show enrichment nearer to telomeres, especially male COs (Fig. 1e and Supplementary Fig. 2c, d). This is broadly similar to patterns observed in other mice^41–43^, humans^21^, and other organisms such as dogs (which have lost *PRDM9*)^44^, although COs show somewhat stronger telomeric enrichment than NCOs. NCOs are also enriched near centromeres, especially on smaller chromosomes (Fig. 1e and Supplementary Fig. 2e). Notably, the telomere effect appears less pronounced among events controlled by the *Prdm9*^*Cast*^ allele (Supplementary Fig. 2f). By examining the joint distribution of COs and NCOs in F2 mice, we confirm that NCOs can occur on both sister chromatids within each pair, regardless of whether each sister also has a CO (Supplementary Table 2). We also found that broad-scale CO and NCO rates have a strong positive association with GC content; after controlling for GC content, we found that COs are less likely to occur in regions belonging to the gene-rich chromatin compartment A^45^ (Supplementary Table 3 and Supplementary Note 4).

Surprisingly and in contrast to events in human females, 99.5% of observed NCO events were simple and comprised contiguous tracts of converted SNPs, with no non-converted SNPs amongst them. Similarly, 99.4% of de novo COs were simple background switches. This implies that complex events are extremely unusual in mice. Moreover, we observed a very high overlap of both CO and NCO events with recombination hotspots identified by ChIP-seq, stronger than observed in humans^21,26,27^, with little recombination in the remainder of the genome. In F2 mice (whose parents have the same F1 genetic background as the ChIP-seq samples), 96% of CO events and 92% of NCO events overlap either DMC1 or H3K4me3 ChIP-seq peaks (adjusted for false-positive NCO events and chance overlap; 84% unadjusted). This reduces only modestly in F5 mice, where only *Prdm9*^*Hum*^-controlled recombination hotspots are active (Supplementary Table 4), so hotspots identified in the heterozygous F1 mouse are still informative for meioses occurring in F4 mice. Our findings also confirm that female recombination mainly occurs in hotspots also active in male mice, in which our ChIP-seq data were gathered, although there may be sex-specific differences in hotspot heat^15^ (Supplementary Fig. 2d).

**Fig. 2:**
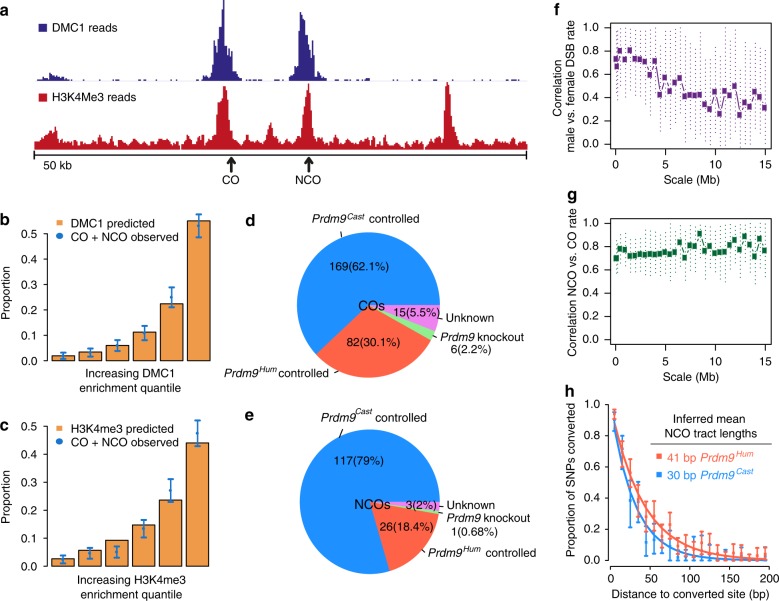
DMC1, H3K4me3, and *Prdm9* allele predict CO and NCO properties. **a** DMC1 and H3K4me3 ChIP-seq peaks in a 50-kb region on Chr10 (starting at mm10 position 122871029), with single NCO and CO events overlapping these peaks. **b**, **c** the proportion of F2 CO + NCO events at hotspots binned by increasing DMC1 (**b**) and H3K4me3 (**c**) ChIP-seq enrichment. **d**, **e** Fraction of COs (**d**) and NCOs (**e**) controlled by the *Prdm9*^*Cast*^ or *Prdm9*^*Hum*^ alleles from F2 mice, overlapping DMC1 hotspots in the *Prdm9* knockout mouse, or non-identifiable (Unknown). **f** Correlation of underlying total recombination rates between females and males (pooling NCO and CO events), for rates binned at different scales (x-axis); dotted lines show 95% CIs for true correlations. **g** As **f**, but showing correlations between (sex-averaged) NCO and CO rates at different scales. **h** Decay in probability that nearby SNPs are co-converted, with inter-SNP distance, conditional on a SNP being converted

NCO and CO events both occur in individual recombination hotspots with probabilities approximately proportional to their estimated heat using either DMC1 or H3K4me3 enrichment (Fig. 2a–c, Supplementary Fig. 3f): over 50% of all hotspot-associated F2 NCO or CO events occur in only the 4,000 hottest hotspots (ranked by DMC1 enrichment), around one sixth of all hotspots. Strong dominance of *Prdm9*^*Cast*^-controlled over *Prdm9*^*Hum*^-controlled hotspots is observed for both event types, and in our ChIP-seq data (Fig. 2d, e and Supplementary Fig. 3a, b). Because binding sites for the humanised allele have not experienced evolutionary hotspot erosion^20^, this phenomenon cannot explain the dominance of the *Prdm9*^*Cast*^ allele. Others have recently provided evidence that higher expression of *Prdm9*^*Cast*^ than *Prdm9*^*Hum*^ is also unlikely as an explanation^40^, although we cannot rule out that the dominance of the *Prdm9*^*Cast*^ allele over *Prdm9*^*Hum*^ is due to the presence of greater levels of (or more stable) PRDM9^Cast^ protein.

Alternatively, or in conjunction, stronger binding affinity of the CAST protein for its motif may underlie the observed dominance. Indeed, both a smaller number and weaker average intensity of PRDM9 (and mirroring H3K4me3) ChIP-seq peaks were recently reported in testes from B6 compared with CAST mice^46^. Similarly, we observe weaker H3K4me3 enrichment at *Prdm9*^*Hum*^-controlled hotspots compared to *Prdm9*^*Cast*^-controlled hotspots (Supplementary Fig. 3e). If occurring also in mice, the propensity of PRDM9^Hum^ to bind to promoters, which have lower DSB and recombination rates in humans and comprise ~10% of its binding sites^16^, might also help make the *Prdm9*^*Cast*^ allele appear even more dominant.

After accounting for sampling variation (Methods), we estimated correlation between recombination rates at different scales (Fig. 2f, g and Supplementary Fig. 3c,d). This revealed sex differences in recombination rates (combining COs and NCOs to gain power), with 100% correlation excluded, and decreasing correlations at broader scales (Fig. 2f). We observe strong (>70%) correlation between sex-averaged NCO and CO rates, although we also find very strong evidence that these events differ in their positioning along the chromosome, especially at broad scales (Fig. 1e and Supplementary Fig. 2), and the NCO rate is much higher than the CO rate at all scales.

### Length, number, and positioning of NCO tracts

We leveraged the high SNP density (~1 SNP per 170 bp) and large number of events in our system to estimate the underlying NCO event tract lengths (accounting for the fact that if a NCO event does not contain a SNP, it is not observed; Methods), separately for hotspots controlled by *Prdm9*^*Cast*^ and *Prdm9*^*Hum*^. The data show relatively good fits to an exponential distribution (Fig. 2h), but with significant differences in estimated mean NCO tract length (*p* = 0.0018 by bootstrapping): 30 bp for *Prdm9*^*Cast*^ (95% confidence interval (CI) 25–35 bp), and 41 bp for *Prdm9*^*Hum*^ (35–48 bp CI). This is unexpected and implies that *Prdm9* alleles can differ in basic properties of how recombination events resolve. We confirmed that this difference cannot be explained by a difference in SNP density surrounding hotspots controlled by each allele (Supplementary Note 5). These tract length estimates are at the lowest end of the broad existing estimates for humans and mice^25–27,38^.

Previous studies using microscopy have reported 200–400 visible DMC1 foci marking individual DSB sites per meiosis in mice^38,47,48^. However, some of these DSBs, e.g. those occurring on the X-chromosome in males, might repair using the sister chromatid, yielding no converted SNPs. Using our sequencing data, we directly estimated the total number of DSBs that repair from the homologue per meiosis. Given our tract length estimates, we inferred an average total of 300.5 DSBs (95% CI 258.5–370.5) per meiosis repairing using the homologue, 90% of these being NCOs^38,49,50^ (Supplementary Note 6). Estimates are very similar in both F1 and F4 parents. This suggests that the vast majority of the estimated 200–400 DSBs per meiosis undergo homologue-templated repair, rather than sister-templated repair^36,51^.

**Fig. 3:**
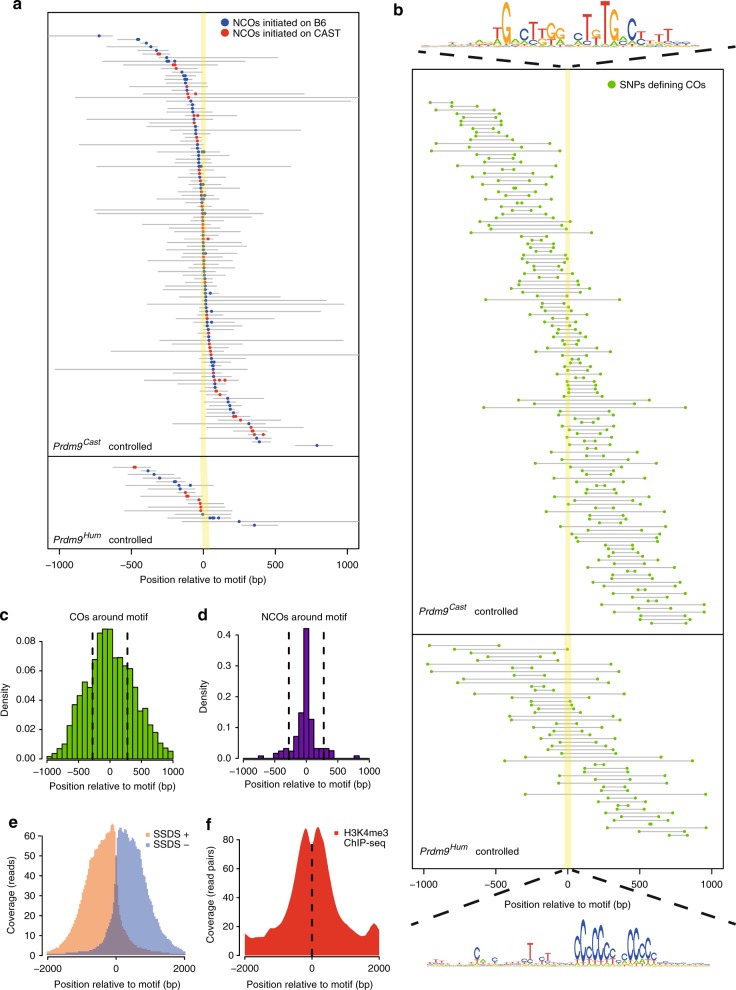
Positioning of NCOs, COs, DMC1, and H3K4me3 around PRDM9 binding motifs. **a** F2 NCOs occurring within hotspots possessing robustly identified PRDM9 binding motifs (114 *Prdm9*^*Cast*^-controlled NCOs and 17 *Prdm9*^*Hum*^-controlled NCOs). Coloured dots are converted SNPs and grey lines represent upper bound of converted tracts. Yellow shading indicates the identified PRDM9 binding target. **b** F2 COs around PRDM9 binding motifs (141 *Prdm9*^*Cast*^-controlled COs and 52 *Prdm9*^*Hum*^-controlled COs). Green dots are SNPs defining CO boundaries within grey delineating regions. COs that have large intervals (>2 kb) between the two defining SNPs are not shown in this plot. **c** Density of COs occurring around motifs. Bar height at each position is proportional to the probability that a breakpoint happens within that bin. **d** Density of NCOs occurring around motifs. The distance between an NCO and motif is defined as the mid-point of the minimal converted tract to the centre of the nearest identified hotspot motif. The distribution was normalised by SNP density in each bin to correct for increased power to see a NCO event where SNP density is high. **e**, **f** Mean DMC1 and H3K4me3 ChIP-seq read coverage around motifs, for the hotspots shown in (**a**)and (**b**). For DMC1, we separated plus strand (SSDS+) and minus strand (SSDS−) reads. Note *x*-axis scale differs from (**c**) and (**d**). Source data for motif logos in **b** are provided as a Source Data file

Both NCO and CO event centres distribute symmetrically around PRDM9 binding motifs that we identified within hotspots (Methods, Fig. 3a–d and Supplementary Fig. 4a–d). NCO events cluster very near to motifs (potentially overlapping them in 70% of cases; Fig. 3a, d), slightly less strongly than clustering of mapped DSB breakpoints^39^, but with a far tighter range than the DMC1 and H3K4me3 ChIP-seq enrichment values (Fig. 3e, f). CO events spread more broadly (Fig. 3b, c), consistent with previous studies^12,25,39,49^. Thus, NCO gene conversion appears restricted to sites very close to initiating DSBs themselves, and more distantly positioned NCOs appear to occur only rarely, in contrast to results from a single mouse hotspot^25^.

### Effects of SNP density on GC-biased gene conversion

**Fig. 4:**
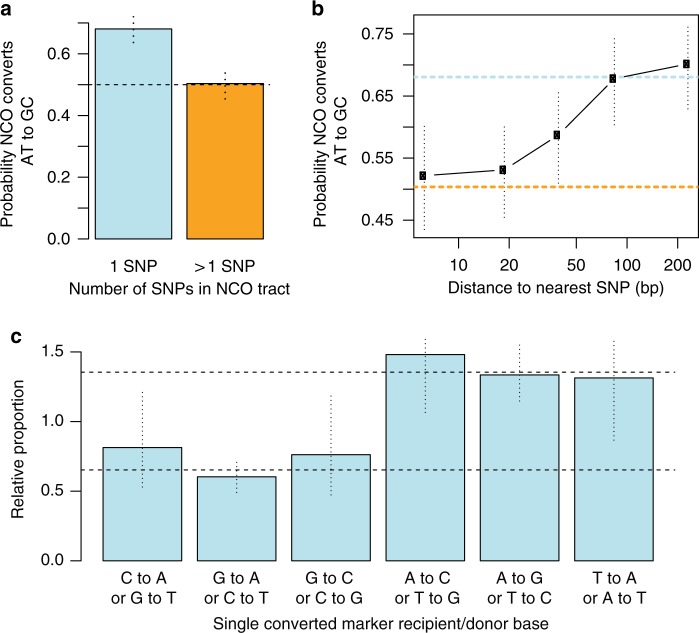
GC-biased gene conversion is absent in multi-SNP NCO tracts. **a** AT-to-GC conversion in single-SNP vs multiple-SNP tracts. **b** GC-bias in groups of converted SNPs, binned according to their distance to the nearest SNP (log-scaled axis). **c** For each of the possible strand-equivalent combinations of NCO donor/recipient alleles (x-axis; e.g. the first bar represents a Strong-to-Weak transversion SNP: recipient C converts to donor A, or G converts to T), the proportion of observed single-SNP NCOs of that type is plotted, relative to the corresponding proportion for the nearest non-converted markers, which lack GC-bias. Vertical lines: 95% CIs (binomial test). Horizontal dotted lines: mean relative proportions for NCO events whose recipient types are G/C or A/T respectively

Our NCO events show strong evidence of AT-to-GC bias, though initially weaker than seen in humans^26^, for both *Prdm9*^*Cast*^-controlled (64%) and *Prdm9*^*Hum*^-controlled (60%) hotspots (*p* < 2 × 10^−9^, two-sided binomial test; Supplementary Table 5). We next focussed on NCO events within *Prdm9*^*Hum*^-controlled hotspots for further investigation, because the genomic GC-content has not evolved alongside this allele. We tested for a difference in NCO tracts containing a single SNP with those containing multiple SNPs (Fig. 4a). Surprisingly, this revealed GC-bias to occur exclusively in single-SNP NCO tracts, which show a near-identical GC-bias (68%) in both males and females (Supplementary Data 5). In complete contrast, no bias (*p* = 0.92, two-sided binomial test) is seen for all multiple-SNP tracts combined, and the difference relative to single-SNP tracts is highly significant (*p* = 1.1 × 10^−7^, Fisher’s exact test). We exactly replicated this finding (*p* = 5.6 × 10^−4^, Fisher’s exact test) in NCO events within hotspots in humans^27^, in both males and females (Supplementary Data 5), so it represents a conserved phenomenon across these mammals. GC-bias strength is unaltered even if DSBs happen only on one homologue (Supplementary Fig. 5a and Supplementary Data 5), implying a mechanism driven by heteroduplex repair^36^ rather than DSB formation^33,35,37^.

A restriction of GC-bias to single-SNP tracts might reflect either some GC-biased process preventing longer events occurring, or a direct impact of the number of SNPs within heteroduplex DNA on whether GC-bias occurs. To distinguish between these possibilities, we stratified SNPs by distance to their nearest neighbouring SNP and measured their GC-bias if they fell within NCO events (Fig. 4b). Strikingly, SNPs near to other SNPs, and therefore almost always co-converted with them, show no GC-bias evidence. Conversely SNPs further than typical NCO tract lengths, >100 bp from the nearest SNP, show the ~68% bias observed in humans, in whom SNP density is much lower^26,27^. This implies that local genetic diversity itself influences GC-biased gene conversion at NCOs, and therefore there must be at least two distinct processes operating to repair heteroduplex stretches formed at DSBs, one which is strongly GC-biased, and another which dominates when multiple mismatches exist, and shows no GC-bias.

To further characterise GC-bias, we estimated conversion rates of different types of SNPs in the donor and recipient chromosomes at single-SNP NCO sites (Fig. 4c and Supplementary Note 7). We normalised these relative to their conversion rates in multi-SNP events (Supplementary Fig. 5b), or to flanking SNP composition (Fig. 4c), both of which show no GC-bias and gave near-identical results. The simplest model which can explain the data is if there are two distinct conversion rates: a lower rate if the recipient chromosome (i.e. the homologue in which the DSB occurs) carries a G or a C, and higher rate if the recipient carries an A or a T. For example, G/C transversions appear to convert at the lower rate. This could be explained by a model where a GC-biased repair process can resolve heteroduplex DNA in favour of the recipient chromosome if it carries a G or C base—effectively blocking conversion of that base.

Notably, we do not observe a consistent GC-bias for CO events, which are accompanied by long conversion tracts of ~500 bp in size^25^. However, we did observe a very small number of complex CO events (0.6% of all de novo COs observed), which contain non-converted markers surrounded by converted markers resulting from the same meiosis. In humans, complex COs have also been observed^35,52^ and have been shown to have GC-bias^27,37^. We hypothesised that complex COs and NCOs might result from blocking of conversion of particular markers where the recipient chromosome carries a G or C base, thus generating a GC-bias in the remaining markers which *are* converted. We observed a total of 12 such non-converted markers within 8 complex NCO events, and 7 within 7 complex CO events. Remarkably, for 18 of these 19 cases the recipient chromosome carries a G or C base (*p* = 7.6 × 10^−5^ by two-sided binomial test). These results suggest that while a GC-neutral mechanism operates for most multi-SNP NCO tracts, producing simple events, occasionally some local, almost 100% GC-biased mechanism may operate at individual SNPs to generate rare complex NCO and CO events.

### CO and NCO depletion when PRDM9 fails to bind the homologue

For NCO events, we can identify on which homologue the underlying DSB occurred. In the F2 mice, we observed a bias: 60% of the observed NCOs were initiated on the B6 background (*p* < 10^−3^, two-sided binomial test). This is due to the behaviour of the *Prdm9*^*Cast*^ allele, which accounts for 80% of observed NCO events (Fig. 2e), and which shows a strong preference for binding to the B6 background (Supplementary Fig. 6a; 66% of NCOs, *p* < 10^−3^, two-sided binomial test), explained by evolutionary hotspot erosion of CAST-controlled hotspots on the CAST genetic background^20,22^. Because PRDM9 binding, DSB formation, and NCO formation are all elevated on the B6 chromosome, this implies that no strong compensation mechanism acts to equalise the number of DSBs or recombination events on different homologues, although weaker compensation that we lack power to detect might occur. As expected, the *Prdm9*^*Hum*^ allele binds and initiates recombination events equally on both backgrounds overall (Supplementary Fig. 6a, *p* = 0.63, two-sided binomial test). Furthermore, the fraction of NCOs initiating on the B6 background (from both sexes) correlates highly with the fraction of testis DMC1 and H3K4me3 ChIP-seq enrichment from that background (Supplementary Fig. 6b–e), confirming similar overall hotspot behaviour in both sexes.

Recombination hotspots can be separated into asymmetric cases where DSBs occur mainly on one homologous chromosome, and symmetric cases where DSBs occur equally on both homologues. Among the most asymmetric hotspots, we observed SNPs or indel polymorphisms within 96% of identified motifs overall (Supplementary Fig. 6f), implying their asymmetry is almost always driven by sequence changes disrupting PRDM9 binding on one homologue. Consequently, asymmetric hotspots remain asymmetric in both sexes and between F2 and F5 animals. As in other hybrid mice^23^, we observed that the ratio of DMC1 to H3K4me3 ChIP-seq enrichment increases roughly two-fold at asymmetric hotspots compared to symmetric hotspots (Supplementary Fig. 6g), a fact most easily explained by delayed repair of DSBs for which the homologue is not bound by PRDM9^23^. To test whether such asymmetry might also change the outcomes of DSB repair, we measured the numbers of NCO and CO events actually occurring in asymmetric vs. symmetric hotspots, relative to their expectations according to DMC1 and H3K4me3 enrichment (Methods and Supplementary Note 8).

**Fig. 5:**
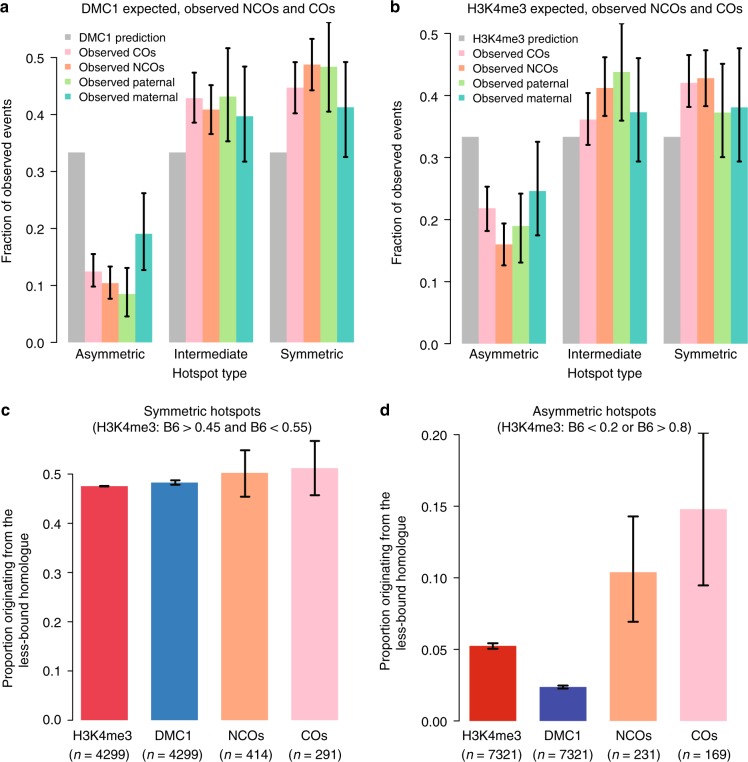
COs and NCOs are depleted in asymmetric hotspots in both sexes. **a** Human-controlled DMC1 hotspots were separated into three bins (asymmetric, intermediate, and symmetric) according to symmetry, so that each bin contains the same number of predicted events according to DMC1 enrichment (Supplementary Note 8). Grey bars show the DMC1-predicted expected fraction of events in each bin. The four coloured bars (vertical lines: 95% CIs) show the observed fraction of (sampled) F5 de novo events: COs, NCOs, and paternal or maternal recombination events. **b** As **a**, except predicted events were defined using H3K4me3. **c**, **d** Expected vs observed initiation biases at symmetric (**c**) and asymmetric (**d**) hotspots, as defined by H3K4me3 symmetry. If asymmetry-related recombination deficiency occurred equally on both homologues, then the fraction of DMC1 reads and the fraction of NCO and CO initiations on the less-bound homologue would be predicted to closely match its fraction of H3K4me3, as in the symmetric case (**c**). Error bars are 95% bootstrap CIs

With all else being equal we expect any two groups of hotspots that are matched to have the same total DMC1 or H3K4me3 enrichment to also have similar numbers of CO and NCO events (Fig. 2b, c). However, when we grouped *Prdm9*^*Hum*^-controlled hotspots according to their (a)symmetry we instead observed a strong depletion of both NCO and CO events in the most asymmetric hotspots (*p* = 10^−27^ and *p* = 10^−23^, chi-square test, respectively, after controlling for factors influencing power; Fig. 5), whether DMC1 or H3K4me3 was used. We replicated this signal for *Prdm9*^*Cast*^; for both males and females; and for de novo and inherited events in F5 mice, as well as events in F2 mice (Supplementary Note 8 and Supplementary Fig. 7), so this is a general property of asymmetric hotspots in both sexes. Because the *Prdm9*^*Hum*^ allele in particular did not co-evolve alongside the mouse genome, asymmetric hotspots controlled by this allele arise from sequence variants that overlap and disrupt PRDM9^Hum^ binding sites on one homologue or the other by chance (i.e. not due to historical hotspot drive). This implies a mechanistic impact of asymmetry on recombination independent of hotspot erosion or other evolutionary forces^23^.

Importantly, we found that this homologous recombination deficiency is driven by PRDM9 binding asymmetry alone (measured by homologue-specific H3K4me3 enrichment), rather than SNP diversity elsewhere within hotspots (Supplementary Table 6). Furthermore, for DSBs occurring on the less-bound chromosome of asymmetric hotspots (measured by homologue-specific DMC1 enrichment), we found that NCO events occur at the expected rate for symmetric hotspots (Supplementary Note 8). Similarly, we observe that a greater *fraction* of both CO and NCO events initiate on the less-bound homologue compared to the expectation from H3K4me3 or DMC1 asymmetry (Fig. 5c, d). We also observe a reduced fraction of DMC1 enrichment relative to H3K4me3 enrichment on the less-bound homologue, consistent with the more-bound homologue taking longer to repair (Fig. 5c, d). This implies that when DSBs occur at asymmetric hotspots on the more frequently bound chromosome, the resulting lack of observed NCO or CO recombination events must be specifically driven by a lack of PRDM9 binding to its homologue, rather than any impact of diversity per se, even within PRDM9 binding motifs.

## Discussion

Our results indicate a sex-averaged NCO rate in mice carrying humanised *Prdm9* of around 10^−6^ per base, which is strikingly below human estimates of around 4.1 × 10^−6^ and 7.7 × 10^−6^ in males and females, respectively^27^. We estimate that there are 300.5 total COs and NCOs per meiosis in mice, consistent with previous microscopy studies showing there are 200–400 DSBs per meiotic cell^38,47,48^. This implies that most DSBs in mice undergo homologue-templated repair, and it suggests that the true number of NCOs per meiosis in mice is unlikely to be much larger than our estimate. The greater overall NCO rate in humans may be due in part to the greater frequency of long, complex NCOs in humans, which can each convert a large number of sites^26,27^. These long, complex events can extend up to 100 kb and comprise up to 11.3% of human NCOs detected by sequencing; they were also found to be enriched in females and to increase in frequency with maternal age^27^. We found that similar events are nearly absent in mice (0.5% of all NCO events detected in this study, and never as long as those seen in humans). We suggest that this difference may reflect the timespan of dictyate arrest, which occurs before the completion of female recombination, and lasts decades in humans vs. months in mice. These findings support the hypothesis that complex NCO events in humans might arise from the repair of non-programmed DNA damage occurring over time, consistent with the fact that they are different in other ways: they mainly occur outside *PRDM9*-controlled hotspots, they are often longer than 1 kb, and they show GC-bias regardless of their size^27^.

Using DMC1^40^ and H3K4me3 ChIP-seq data, one can infer that DSBs form at a small subset^12^ of PRDM9 binding sites in each cell on average proportionally to the rate of PRDM9 binding to each site^23^. However, we find that the processing, repair and eventual recombination outcomes at each PRDM9 binding site all depend strongly on the sequence of the homologue at that site. In this study, we revealed for the first time that both CO and NCO events are depleted, in both sexes, at asymmetric recombination hotspots (relative to expectations from both H3K4me3 and DMC1 enrichment; Fig. 5 and Supplementary Fig. 7). That is, when the unbroken homologue is not strongly bound and/or marked by PRDM9 (primarily due to motif-disrupting SNPs on the homologue; Supplementary Fig. 6f), the DSB seems less able to repair by homologue-templated recombination. This effect cannot be explained by an increase in genetic diversity alone, as has been suggested^22^; we found that only nearby SNPs that abolish PRDM9 binding symmetry have any association with the CO or NCO rate (Supplementary Table 6). One potential consequence of the reduction of homologue-templated repair at asymmetric hotspots is the mitigation of hotspot erosion caused by the over-transmission of alleles that disrupt PRDM9 binding^20,23^.

A recent crossover-mapping study, which sequenced single sperm from the same mouse cross examined here, similarly found CO depletion and DMC1 excess at asymmetric hotspots^40^. Here we have gone further by demonstrating that both COs and NCOs are depleted at asymmetric sites, showing that many DSBs at asymmetric hotspots are not just delayed in their repair, but they often completely fail to ever repair from their homologue. Moreover, this occurs in female as well as male meiosis. This supports the hypothesis that homology search is the key process disrupted at asymmetric hotspots, rather than downstream events like CO versus NCO repair decisions. One hypothesis is that PRDM9 binding and/or its associated chromatin marks on the unbroken homologue assist the homology search machinery with the challenging task of finding the correct homologous template for repair. This could explain the wider asynapsis and infertility seen in male mice where asymmetric hotspots predominate^23,53^, although additional factors must act to explain sex differences in hybrid fertility. There still remains the question of how asymmetric hotspots are repaired, if not from the homologue, in hybrid mice whose fertility is not disrupted. We suggest that asymmetric hotspots sometimes behave like DSBs on the X chromosome in males, which repair late from the sister chromatid and, like asymmetric hotspots, show excess DMC1 enrichment^18,23^. Because sister-templated repair does not convert any SNPs, we are unable to directly detect such events.

Our results demonstrate that local genetic differences between homologues can also affect the process of GC-biased gene conversion (gBGC) at NCO sites. We confirmed gBGC does not depend on DMC1 enrichment or hotspot symmetry (Supplementary Fig. 5 and Supplementary Data 5), and this implies it must operate downstream of DSB formation, during heteroduplex mismatch resolution. We found that within hotspots in both humans and mice, gBGC acts almost exclusively on potential conversion tracts containing only a single SNP (i.e. mismatch), with essentially identical bias in each species (68% of NCOs convert A/T to G/C; Supplementary Data 5)^26,27^. In contrast, nearly all multi-SNP NCO tracts are simple stretches of converted markers with no apparent GC-bias, implying more than one pathway for heteroduplex repair. It has been hypothesised that gBGC may function to oppose the mutagenic effects of recombination^37^; our results suggest that this effect is weakened at sites with high local heterozygosity, which are also predicted to have a higher gene conversion rate.

**Fig. 6:**
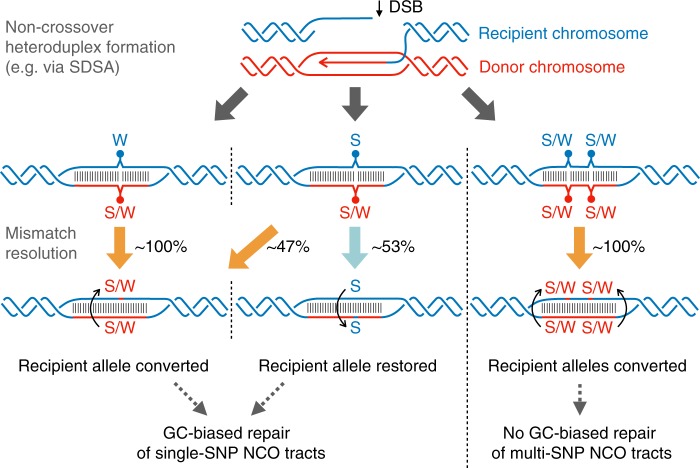
Possible model of NCO mismatch repair pathway choice. NCO repair pathways such as Synthesis-Dependent Strand Annealing (SDSA) form heteroduplex DNA between the recipient homologue’s original sequence (blue) and a small tract of DNA copied from the donor homologue (red). Any mismatching bases must be resolved either by restoring to the recipient allele or converting to the donor allele; only the latter can be detected in offspring. Three possible NCO heteroduplex tracts are depicted, differing in the number and type of mismatch sites (these regions are illustrated as unwound for visual aid purposes only). In all cases, one proposed mismatch repair mechanism (orange arrow) converts site(s) on the recipient chromosome in a donor-strand-biased manner to the allele of the donor chromosome (red). However, in the particular case shown in the middle column—where there is a single mismatch site and the recipient chromosome contains a Strong (S = G or C), not Weak (W = A or T), allele—a different proposed repair mechanism (blue arrow) operates ~53% of the time and restores the G/C recipient allele in a recipient-strand-biased manner, resulting in no observable gene conversions at that site (Supplementary Fig. 8 and Supplementary Note 7). The sum of these two effects can explain the overall 68% bias of observed gene conversions towards G/C

As one possible model to explain these gBGC observations, we propose that there are two strand-aware heteroduplex repair pathways at NCOs (illustrated in Fig. 6). The first proposed pathway can repair all types of heteroduplex mismatches and always selects the bases copied from the donor homologue—we call this the donor-biased pathway. The second proposed pathway operates only at heteroduplexes containing a single mismatch with a G or C base on the recipient strand, and it always selects the recipient strand’s base—we call this the GC-restoring pathway. All mismatches repaired by the donor-biased pathway will be observable as gene conversions. However, because the GC-restoring pathway restores the recipient allele, its outcome cannot be directly observed (equivalently, we say gene conversion is blocked at that site). While we cannot formally rule out other possible models of NCO repair, our proposed strand-aware model can fully explain our observations of selective GC-bias as well as our number of observed NCO events while only requiring ~300 total DSBs per meiosis. In contrast, a strand-unaware model would require ~465 DSBs per meiosis, which is outside the range of previous experimental observations^38,49,50^ (Supplementary Fig. 8b and Supplementary Note 7). Furthermore, at 19 observed CO events within highly (>95%) asymmetric hotspots containing a mutation within their PRDM9 motif, we observe transmission of the cold binding site allele to offspring in 95% of cases (Supplementary Note 7), as has been previously observed at an individual hotspot^20^. Therefore, for those longer conversion tracts within CO events at least, a strand-aware mechanism of heteroduplex repair appears to bias repair towards the unbroken donor homologue.

Importantly, both proposed pathways can repair single-GC recipient sites: we calculated that the GC-restoring pathway would need to operate at these sites 53% of the time to account for the observed 68% overall GC-bias among observed events (Fig. 6, Supplementary Fig. 8a, Supplementary Note 7). For example, the GC-biased mechanism might operate only at G or C recipient bases, but not both, which would cap the bias at NCO sites to (at most) 67%, very close to the observed fraction. Almost all observed complex NCOs and COs, though very rare, appear to be explained by this GC-restoring pathway operating on individual SNPs within the tract, with the recipient homologue carrying a G or C base in 95% of such non-converted markers we observed (Supplementary Note 7). Similar behaviour was observed at complex COs within a single human hotspot, suggesting a conserved mechanism across species^37^. Because the proposed GC-biased pathway operates at single SNPs and shows strong base-specific and strand-specific biases, it may involve proteins like those found in the base excision repair (BER) pathway, as previously suggested^29^. In contrast, the proposed donor-biased repair pathway, which can operate on longer stretches including multiple SNPs, is more consistent with proteins in the mismatch repair (MMR) pathway, several of which are known to be essential for meiosis in mice^54,55^.

Here we have uncovered the first examples of local genetic diversity affecting both the ability to undergo homologue-templated repair (due to polymorphisms altering PRDM9 binding symmetry), and the decision to repair via GC-biased gene conversion (depending on the number and spacing of local sequence polymorphisms), both downstream of DSB formation. Another unexpected influence on NCO events, again occurring downstream of DSB formation, is the *Prdm9* allele, with *Prdm9*^*Hum*^-controlled NCOs having an average length 11 bp (37%) longer than *Prdm9*^*Cast*^-controlled NCOs. It is unclear whether this reflects PRDM9 binding directly, or some indirect impact, e.g. how PRDM9 binds relative to nucleosome positions or other genetic or epigenetic features. Further work will need to investigate the exact mechanisms by which PRDM9 binding to the unbroken homologue promotes homologue-templated repair as well as the exact repair pathways that can explain why NCO recombination outcomes depend on local heterozygosity.

## Methods

### Mouse breeding, library preparation and sequencing

CAST/Eij (CAST) mice were sourced from MRC Harwell (UK). The C57BL/6 J (B6) line humanised at the *Prdm9* zinc-finger array (B6^Hum^) was generated previously^23^. Breeding of CAST and B6^Hum^ mice (F0) was carried out in both directions (using females and males of each type) to generate (B6xCAST)F1 hybrid, heterozygous offspring. To study properties of the humanised *Prdm9* allele, we genotyped as previously^23^ and selected 26 F2 mice of each sex homozygous for humanised *Prdm9*. We crossed these animals and their offspring for three further generations and selected 72 F5 offspring, comprising two of each sex from each of 18 pairs of F4 parents. One B6^Hum^ mouse, one CAST mouse, 11 F2 mice and all 18 F4/F5 families (36 F4 parents and 72 F5 offspring) were subjected to whole genome sequencing. Genomic DNA was extracted from spleen using the DNAeasy Blood and Tissue Kit (Qiagen), according to the manufacturer’s instructions. Libraries were prepared by the Oxford Genomics Centre at the Wellcome Centre for Human Genetics (Oxford, UK) using established Illumina protocols (with a Nextera DNA Library Prep Kit, fragmented to an average of 500 bp).

We sequenced to obtain coverage of ~10× for the F0/F4 mice, and 20x for the F2 and F5 mice via the Illumina Hiseq2500 (F0 and 4 F2 mice; paired 100-bp reads) or Hiseq4000 platforms (remaining mice; paired 100-bp reads for 7 F2 mice, paired 150-bp reads for F4 and F5 mice). Sequencing reads were aligned to mm10 using BWA^56^ (v. 0.7.0) followed by Stampy^57^ (v. 1.0.23, option bamkeepgoodreads). We used Picard tools (v. 1.115) (http://broadinstitute.github.io/picard) to merge bam files from different lanes for the same sample and identify duplicate reads. GenomeAnalysisTK-3.3-0 (GATK) was used for local indel realignment followed by base quality score recalibration, and variant calling, using known indel targets and SNPs between B6 and CAST from the Mouse Genome Project (MGPv4) data^58^. We filtered variants using GATK’s Variant Quality Score Recalibrator (VQSR), employing the set of variants present on the Affymetrix Mouse Diversity Genotyping Array as a set of true positive variation^59^. We used the annotations “HRun”, “HaplotypeScore”, “DP”, “QD”, “FS”, “MQ”, “MQRankSum”, and “ReadPosRankSum” to train VQSR, and a sensitivity threshold of 90% for the true positive set to define the set of newly genotyped sites that passed VQSR filtration. Heterozygous sites within the F0 individuals will mimic observed NCOs in the F2 mice. To remove potential hidden heterozygous sites within the F0 individuals, we removed all variants not genotyped as matching the homozygous reference allele in B6, or the homozygous alternative allele for CAST from MGPv4^58^. We obtained 13,946,562 and 13,940,079 reliable autosomal SNPs from the F2 and F5 samples, or roughly one SNP for about every 170 bp, which were used for downstream analysis.

### Identifying unique NCO and CO events

Using the HMM method described in the Supplementary Note 2 to define a background state (homozygous CAST background, heterozygous, or homozygous B6 background) along the genome in each mouse, we identified state changes as CO events. Autosomal genotypes in F2 and F5 mice conflicting with their background were investigated as potential NCO events, but mainly represented sequencing errors. The region chr6:37000000–56000000 (mm10) was removed since it was observed to be not fully inbred in the F0 founders. We filtered to remove false-positive sites (Supplementary Table 1), apparently heterozygous sites in the F0 mice, false heterozygous calls exhibiting unequal numbers of reads supporting the two alleles, false homozygous calls due to low read depth, and others. About 99.97% of potential converted sites are removed. For example, the number of potential converted sites dropped greatly e.g. from 863,082 SNPs potentially converted to 183 distinct identified NCOs (240 SNPs) within the F2 mice.

For COs and NCOs identified in F5 animals, they were treated as inherited if the parents carried an identical event, and otherwise de novo. We identified 821 de novo COs, 1384 inherited COs, 510 de novo NCOs, and 882 inherited NCOs; thus about 37% of the events are de novo for both COs and NCOs. We used a previously described HMM algorithm^60^ to identify parent-of-origin in de novo COs (those occurring in the germ cells of the F4 parents). For de novo NCOs, we were only able to confidently assign parental origin when one of the parents was heterozygous at the converted sites, while the other was homozygous. In this case, the NCO must be inherited from the heterozygous parent.

We removed duplicate inherited CO and NCO events, yielding a set of unique events for downstream analyses. Of 1,575 observed NCO events, only eight were complex and involved background switching within the event. Among these eight NCOs, two of them are F5 de novo NCOs and six of them are inherited NCOs detected from F5 animals. Seven of them overlap a hotspot. Of 1,116 observed de novo CO events from F2 and F5 animals, seven were complex e.g. a CO accompanied by a NCO event. Six of them are from F5 de novo events and one of them is from an F2 animal. Of these seven events, six of them overlap a hotspot.

### NCO validation by Sanger sequencing

Of the 88 NCO events detected in F2 mice, we selected a subset for validation including 19/79 events located within a hotspot (prioritising events located within a single 500 bp region and excluding those located within repetitive regions), as well as all nine events located outside a hotspot. We PCR-amplified short regions (around 500 bp) overlapping the identified NCO sites using genomic DNA from the two F0 mice, the F2 mouse carrying the NCO, and up to three other related and/or unrelated F2 mice, using standard conditions (see Supplementary Table 7 for primer sequences). PCR products were purified using the QIAquick PCR Purification Kit (Qiagen) and analysed by direct Sanger sequencing (Source Bioscience, UK). Sequence data comparison and analysis was carried out using Chromas LITE (version 2.1.1). By comparison of genotypes, we identified true NCOs vs. false positives. This confirmed all 19 NCOs overlapping a hotspot (100%), and of nine non-hotspot cases, four were confirmed (44%). This suggests almost all hotspot-overlapping NCO events are likely real, and a higher false-positive rate in NCOs away from hotspots. Given that 84.2% of the F2 NCO events overlap hotspots (Supplementary Table 4), we estimate an overall fraction of validated detected NCO events as 0.842 + 0.44 × 0.158, i.e. 91.1%.

### Estimating power to identify NCOs

To estimate the power of our method to detect NCO events of varying tract lengths, we simulated NCOs with different mean tract lengths and ran our pipeline for identifying NCO events, including our filters. Because F2 events are controlled by both *Prdm9*^*Hum*^ and *Prdm9*^*Cast*^ and F5 de novo events are controlled by *Prdm9*^*Hum*^ alone, we performed two sets of simulations by using data from 11 F2 samples and 72 F5 samples. Because most recombination events overlap hotspots, we simulated NCOs in hotspot regions. For each mean tract length, we sampled 2,000 hotspots with probabilities proportional to their H3K4me3 enrichment. Within each hotspot, we sampled the centre of the NCO tract according to the distribution of NCOs around PRDM9 motifs after correcting for SNP density, and we sampled its tract length from an exponential distribution with a pre-defined mean tract length (which we varied from 10 to 100 bp with step size 10 and from 150 to 300 bp with step size 50). Sampled NCO tracts containing 0 SNPs were not counted as potentially detectable. Across these 2000 tracts, different animals possessed different ancestral backgrounds. For each tract in each animal, we checked if any of the other animals had a different ancestral background consistent with a gene conversion event in the first animal. If so, we sampled such a donor mouse (other events were ignored). We copied the sequencing information corresponding to the converted sites from the donor mouse, such as the allele depth, and we copied the sequencing information for the background from the recipient, such as mate-pair information. We copied the variant call information rather than reads themselves so all the information we copied was downstream of read mapping and variant calling. This copying process mimics the recombination process and the output would mimic true NCOs. Then, we applied the same filters to this simulated sequencing data at each sampled tract. We calculated our power by dividing the total number of simulated tracts left after filtering by the total number of simulated tracts overlapping at least one SNP (Supplementary Fig. 2a, b).

### H3K4me3 ChIP-seq

We performed ChIP-seq against H3K4me3 in testes from two littermate 23-week-old male (B6xCAST)F1-*Prdm9*^*Hum/Cast*^ mice (C57BL/6J-*Prdm9*^*Hum/B6*^ mother, CAST/Eij father; two biological ChIP replicates—one testis per mouse—plus one input chromatin replicate from one mouse) as previously described^23^ with several important modifications that increased ChIP stringency (underlined in the full protocol below). In brief, the testis tunica was removed, the seminiferous tubules were disassociated with tweezers and fixed at room temperature in 5 ml 1% formaldehyde in 1× PBS for 5 min followed by glycine quenching (125 mM final conc.). Following 2× centrifugation (2000 × *g*) and resuspension in 4 °C 1× PBS, pellets were snap-frozen using dry ice and stored at −80 °C. Frozen pellets were thawed and resuspended in 900 μl 1% SDS lysis buffer (1% SDS, 10 mM EDTA, 50 mM Tris pH 8.0, 2× protease inhibitors), dounced 20 times and sonicated in 300 μl aliquots in a Bioruptor Twin (Diagenode) sonication bath at 4 °C for three 5-min periods of 30 s on, 30 s off at high power, then cell debris was pelleted (14,000 × *g*, 15 min, 4 °C) and removed and aliquots were pooled. Sonicated lysates were diluted 1:10 in cold IP wash buffer (100 mM Tris pH 7.5, 500 mM LiCl, 1% NP-40, 1% sodium deoxycholate, filtered with a 0.45 micron filter unit) for antibody incubation. Magnetic beads were washed by adding 50 μl Invitrogen Sheep Anti-Rabbit Dynabeads (Thermo Fisher Scientific) per sample to 950 μl cold PBS/BSA (1× PBS, 5 mg/ml BSA, one tablet Roche Complete protease inhibitor per 50 ml, filtered with 0.45 micron filter). Bead solutions were placed on a magnetic rack and resuspended in 1 ml PBS/BSA four times. The washed beads were resuspended in 100 µl PBS/BSA per sample and added to the diluted chromatin samples for pre-clearing for 2 h at 4 °C with rotation. Beads were removed, and 100 µl of pre-cleared chromatin was set aside for the input control. Five microlitres of rabbit polyclonal anti-H3K4me3 antibody (Abcam ab8580) were added to the remaining pre-cleared chromatin and incubated overnight at 4 °C with rotation. 50 µl beads were washed and resuspended in 1× PBS/BSA as before, then incubated with the diluted chromatin samples for 2 h at 4 °C with rotation. Beads were then washed five times for 3 min each with 10 ml cold LiCl Wash Buffer (100 mM Tris pH 7.5, 500 mM LiCl, 1% NP-40, 1% sodium deoxycholate, filtered with a 0.45 micron filter unit), then washed once with cold 1X TE (10 mM Tris-HCl pH 7.5, 0.1 mM Na_2_-EDTA). Bead pellets were resuspended in 200 μl room-temperature IP elution buffer (1% SDS, 0.1 M NaHCO3, filtered with a 0.45 micron filter unit) and vortexed to mix. Samples were incubated in a 65 °C water bath for 1 h with mixing at 15 min intervals to uncouple beads from protein-DNA complexes. Samples were centrifuged (14,000 rpm, 3 min) and placed on a magnet to pellet beads, and supernatants were isolated and then incubated in a 65 °C water bath overnight to reverse crosslinks. For the input control, 50 µl of pre-cleared chromatin was processed in parallel. After de-crosslinking, samples were further incubated with 80 µg RNase A at 37 °C for 60 min and then with 80 µg Proteinase K at 55 °C for 90 min. DNA was purified using a Qiagen MinElute reaction cleanup kit and quantified using a Qubit High Sensitivity DNA kit (Thermo Fisher Scientific). This yielded roughly 1 ng of ChIP DNA per testis.

ChIP and total chromatin DNA samples were sequenced in multiplexed paired-end Illumina HiSeq2500 libraries (rapid run), yielding 63–71 million 51-bp read pairs per replicate after filtering. Sequencing reads were aligned to mm10 using BWA^61^ (v0.7.0-r313, option -q 10) followed by Stampy^57^(v1.0.23-r2059, option -bamkeepgoodreads), and reads not mapped in a proper pair or with an insert size larger than 10 kb were removed. Read pairs representing likely PCR duplicates were also removed by samtools^61^ rmdup (v0.1.19–44428 cd). Pairs for which neither read had a mapping quality score greater than 0 were removed. Fragment coverage from each replicate was then computed at each position in the genome using in-house code and the samtools^61^ (v0.1.19–44428 cd) and bedtools^62^ (v2.23.0, genomecov -d) packages. Peak calling was performed using our published peak calling algorithm (fully described, open-access, and open-source, in Altemose et al.^16^). Sets of broad H3K4me3 peaks were called by testing for enrichment in 100 bp bins across the genome then merging adjacent significantly enriched bins (at *p* < 10^−5^), which were used for most analyses including overlap calculations. Narrow peaks were called using 1 bp bins and requiring peak centres to be at least 1 kb apart (with a threshold of *p* < 10^−6^); these were used for the broad-scale plots of H3K4me3 enrichment and for the broad-scale GLM analysis. H3K4me3 enrichment was also force-called in 1 kb bins surrounding DMC1 peaks, again using the Altemose et al. algorithm^42^ (these are reported in Supplementary Data 2). PRDM9-independent H3K4me3 peaks were identified by taking the union of all peak regions shared between various pairs of previously published mice with non-overlapping *Prdm9* alleles (*Prdm9*^*PWD/H*^, *Prdm9*^*PWD/B6*^, *Prdm9*^*Hum/Hum*^, *Prdm9*^*B6/B6*^)^23^. The percentage of ChIP-seq read pairs originating from signal (as opposed to background) was estimated to be 87.4%, a significant improvement over our prior, less stringent, experimental method (which yielded 62–71% of read pairs from signal)^23^.

### DMC1 ChIP-seq

DMC1 ChIP-seq data were generated elsewhere^40^. Briefly, single-stranded DNA sequencing (SSDS) DMC1 ChIP-seq was performed as described previously^13^, using testes from a male (B6xCAST)F1-*Prdm9*^*Hum/Cast*^ mouse. ChIP and total chromatin DNA samples were sequenced in multiplexed paired-end Illumina HiSeq2500 libraries (rapid run), yielding 252 million 51-bp read pairs. We processed the data following the algorithm provided by Khil et al.^13^ to map the reads to mm10 and obtain type I reads. We then called DMC1 peaks as previously^23^. We defined NCO and CO events as occurring within hotspots if they were less than 1 kb away from either a DMC1 peak or an H3K4me3 peak (covering 4% of the genome).

### Testing for correlation rates at chosen scales

We assumed that given an underlying vector of (binned) mean values *W*_*k*_ along the genome, the *k*^th^ recombination-related quantity (number of observed recombination or NCO events in various classes), *N*_*ik*_, follows a Poisson distribution with mean *W*_ik_, in interval *i*. The *W*_*k*_ means vary along the genome and represent the underlying recombination rate parameters; this model is accurate provided (as is likely to be case) for a single meiosis, the number of expected events in each bin is small. Then the variance

1 $${\mathrm{Var}}\left( {N_{ik}} \right) = {\mathrm{E}}\left( {{\mathrm{E}}\left( {N_{ik}^2W_{ik}} \right)} \right) - {\mathrm{E}}\left( {W_{ik}} \right)^2 = {\mathrm{E}}\left( {W_{ik}} \right) + {\mathrm{Var}}\left( {W_{ik}} \right)$$

This enabled estimation of the variation in recombination rate along the genome, using the usual standard estimates of the mean and variance of the number of events, across bins genome-wide:

2 $${\mathrm{Var}}\left( {W_{ik}} \right) = {\mathrm{Var}}\left( {N_{ik}} \right) - {\mathrm{E}}\left( {N_{ik}} \right)$$

Further, the covariance

3 $${\mathrm{Covar}}\left( {N_{ij},N_{ik}} \right) = {\mathrm{E}}\left( {{\mathrm{E}}\left( {N_{ij}N_{ik}|W_{ij},W_{ik}} \right)} \right) - {\mathrm{E}}\left( {W_{ik}} \right){\mathrm{E}}\left( {W_{ij}} \right) = {\mathrm{Covar}}\left( {W_{ij},W_{ik}} \right)$$

Combining these results enabled estimation of the underlying correlation between *W*_*j*_ and *W*_*k*_ along the genome based on properties only of the observed Poisson counts *N*_*j*_ and *N*_*k*_:

4 $${\mathrm{Cor}}\left( {W_{ij},W_{ik}} \right) = \frac{{{\mathrm{Covar}}\left( {N_{ij},N_{ik}} \right)}}{{\sqrt {{\mathrm{Var}}\left( {N_{ij}} \right) - {\mathrm{E}}\left( {N_{ij}} \right)} \sqrt {{\mathrm{Var}}\left( {N_{ik}} \right) - {\mathrm{E}}\left( {N_{ik}} \right)} }}$$

The quantities in the above equation are all estimated in the usual way using standard estimates of mean and variance from the observed vectors of counts. At any interval size scale, we bootstrap re-sampled (10,000 times) the resulting disjoint intervals of the genome, to compute CIs for the estimator.

### Motif analysis

We used a Bayesian, ab initio motif finding algorithm to identify motifs within DSB hotspots^16,23^. For each DSB hotspot that is controlled by *Prdm9*^*Cast*^, a 1,000 bp sequence (centred on the hotspot centre) was extracted from the reference sequence (mm10). *Ab initio* motif identification was performed on the centre 600-bp sequences from the top 1,000 hotspots (ranked by DMC1 enrichment) that contained no bases overlapping annotated repeats. Motif calling proceeded in two stages: seeding motif identification, and motif refinement. Each seeding motif was obtained by first counting all 10-mers present in all input sequences, and from the top 50 most frequently occurring 10-mers, the one with the greatest over-representation in the central 300 bp of each peak sequence was chosen. This seeding 10-mer was then refined for 50 iterations as described in Davies et al.^23^. This refined motif was then force-called on the full set of the hotspots (without filtering) by re-running the refinement algorithm, providing a probability of motif occurrence within each hotspot, and also identifying the most likely motif location in each case. This motif was reported for each peak, along with position and strand. We did the same for DSB hotspots controlled by *Prdm9*^*Hum*^ and a 48-bp human motif was identified. We identified distinct sequence motifs, and their locations, within 97% of hotspots controlled by *Prdm9*^*Cast*^ and 74% of hotspots controlled by *Prdm9*^*Hum*^ (Supplementary Fig. 4a)^20,23,27,63^.

We used the SNPs generated as described above to determine whether each motif contains a SNP within its span. The distance from a motif to an event was defined as the distance from the centre of the motif to the nearest converted marker (lower bound for NCOs), or zero if a converted marker fell within the motif itself. We associated events < 1 kb from a motif with that motif-containing hotspot.

### Estimation of NCO tract lengths

To estimate NCO tract length, we assumed the converted tract follows an exponential distribution with rate parameter *λ*, where 1/*λ* is the mean tract length. While exponential tract lengths are not a fully accurate model, we can view this as a summary of tract properties, estimating the probability of co-conversion of pairs of markers as the distance between them increases. We computed a composite likelihood function for our NCOs and estimated *λ* via maximal likelihood. Specifically, for each converted site, viewing this site as a focal site, we examined the SNPs nearby and recorded for each SNP its distance from the focal SNP, and whether that SNP was also converted. If the SNP was also converted, then it was still in the gene conversion tract, otherwise it was not. Using this approach allowed our approach to be independent of SNP density, because we conditioned on SNP positions in our analysis. The probability that a SNP nearby a converted site is also converted is

5 $$\Pr \left( {{\mathrm{SNP}}\;{\mathrm{nearby}}\;{\mathrm{converted}}} \right) = \Pr \left( {{\mathrm{in}}} \right) = {\mathrm{e}}^{ - \lambda d}$$

where *d* is the distance from the nearby SNP to the converted site. We multiply these probabilities across all focal/nearby SNP pairs to obtain the (composite) likelihood of the data:

6 $$\Pr \left( {\mathrm{D}} \right) = \mathop {\prod}\limits_{{\mathrm{all}}\_{\mathrm{pairs}}} {\Pr \left( {{\mathrm{in}}} \right)^x\left( {1 - {\mathrm{Pr}}\left( {{\mathrm{in}}} \right)} \right)^{1 - x}}$$

where *x* = 1 if the SNP nearby is also converted and *x* = 0 otherwise. By maximising the likelihood using grid search for 1/*λ* from 1 to 1000 with step 0.1, we gained an estimate of tract length. Although distinct NCO events are reasonably modelled as independent, because pairs of SNPs within the same NCO event are not in fact independent, this is not a true likelihood (though the resulting estimator is statistically consistent as the number of independent conversion events increases). Therefore, to estimate uncertainty in the resulting estimates, we utilised bootstrapping of NCO events.

To perform bootstraps, we separated autosomal genomes into 258 non-overlapping 10 Mb blocks (the last block in each chromosome is shorter than 10 Mb). We re-sampled 258 blocks with replacement, where the probability of sampling each block is proportional to the length of that block, and from the resulting bootstrapped set of NCOs, re-estimated tract length via the same procedure. CIs were calculated from a total of 10,000 bootstraps. We implemented this procedure for two sets of NCO events; those overlapping human-controlled and those overlapping CAST-controlled hotspots, respectively. Specifically, we used events that overlap a DMC1/H3K4me3 peak to avoid using any false positives. For *Prdm9*^*Hum*^, we used de novo F5 NCOs along with F2 and inherited F5 NCOs controlled by *Prdm9*^*Hum*^ (815 events total). For *Prdm9*^*Cast*^, we used F2 and inherited F5 NCOs controlled by *Prdm9*^*Cast*^ (409 events total).

### Hotspot symmetry estimates

Sequence differences between the CAST and B6 genomes allowed us to quantify the fraction of ChIP-seq enrichment (either DMC1 or H3K4me3), coming from the B6 and CAST chromosomes. This also allowed us to determine whether individual hotspots in these hybrids were symmetric, with DSBs occurring equally on both chromosomes, or asymmetric, with a preference towards either the CAST or B6 chromosome.

Using SNPs distinguishing the B6 and CAST genomes, each type I read pair from a hybrid DSB library (DMC1 ChIP-seq) was assigned to one of the categories “B6”, “CAST”, “unclassified” or “uninformative” as in ref. ^23^, replacing PWD (*Mus musculus musculus* PWD/Ph) with CAST. For each DSB hotspot, the B6 cutting ratio was then computed as the fraction of B6 reads mapped within 1 kb of the hotspot centre, over the sum of B6 and CAST reads in that region. We followed a similar approach for H3K4me3 ChIP-seq, further correcting for background as in ref. ^23^. For both DMC1 and H3K4me3, we required ≥ 10 informative reads to define the B6 cutting ratio.

To order hotspots based on their symmetry, if the fraction of cuts estimated on the B6 and CAST chromosome were *x* and 1−*x*, respectively, we defined the overall hotspot symmetry as 4 × (1−*x*), which ranges from 0 for hotspots with events completely on one chromosome to 1 for hotspots with events occurring equally on both chromosomes^23^. We obtained additional results for events initiating on a known homologue by using homologous heat, defined as *xh*, where *h* is the estimated total heat of the hotspot, for events initiating on the CAST chromosome, and (1−*x*)*h* for events initiating on the CAST chromosome (Supplementary Note 8). Separate estimates of hotspot symmetry and homologous heat may be obtained from both H3K4me3 and DMC1 ChIP-seq data, for the same collection of hotspots. Because the H3K4me3 homologous heat captures how well the homologous chromosome is bound by PRDM9, it may be of stronger direct interest; however, homologous heat is only directly identifiable for NCO events, whose initiating homologue is known. For CO events, to be conservative (avoiding assumptions regarding conversion tracts to estimate homologous heat), we mainly used hotspot symmetry instead of homologous heat. For Supplementary Fig. 7 we used average homologous heat, defined as 2*hx*(1−*x*), which averages homologous heat over the strand an event occurs on.

### Estimating fraction of hotspots containing disrupted motifs

To estimate the proportion of hotspots of different levels of initiation on B6/CAST chromosomes containing SNPs within their PRDM9 binding motifs, we filtered to include only hotspots containing a clear motif (posterior probability > 0.99), and at least 20 informative reads in our DMC1 data in order to accurately estimate the proportion of reads from B6, and 5 sequencing reads from each homologue covering the motif region, to provide power to identify variants if present. Supplementary Fig. 6f shows the fraction of hotspots in each binned level of initiation on the B6 chromosome containing a SNP or Indel (using GATK prior to VQSR, or Platypus). Ninety-six percent of identified highly asymmetric hotspots (B6 initiation < 5% or >95% and *p* < 10^−10^ for binomial test of asymmetry) contained such a polymorphism.

### Testing whether asymmetry or SNP density affects repair

We fitted a generalised linear regression model to discern whether hotspot asymmetry or local SNP density better predicts variation of CO and NCO rates depending on genetic variation. For each hotspot containing an identified PRDM9 binding motif, we produced a binary response vector indicating whether an overlapping CO event occurred and fit a binomial generalised linear model. As model predictors, we used:

(i) The symmetry of the hotspot from DMC1
(ii) The log-transformed heat of the hotspot estimated by H3K4me3 enrichment (the H3K4me3 enrichment is incremented by a small value 0.0001 as there are a few hotspots with zero estimated enrichment)
(iii) SNP densities around the PRDM9 binding motif at different scales (±100 bp, ±500 bp, ±800 bp)

We then tested each coefficient for significance, conditional on the others. We separated the analysis for *Prdm9*^*Cast*^-controlled COs (all generated in the meiosis from F1 where there are two different *Prdm9* alleles) and de novo *Prdm9*^*Hum*^-controlled COs (all generated in the meiosis from F4 where there is only one type of *Prdm9* allele) to eliminate any effects of competition between the two alleles. Conditional on H3K4me3 enrichment and hotspot symmetry, SNP density has no significant effect on where COs happen (*p*-values from all three scales > 0.08) while both H3K4me3 enrichment and hotspot symmetry have significant positive effects on CO events conditional on SNP density (*p* < 0.05).

For NCO events, we performed a similar analysis, except that we corrected for power to detect NCOs by re-sampling the above hotspots according to the weight generated as described in Supplementary Note 8. We note that some hotspots appeared several times after rejection sampling. Again, all scales showed no significant effect of SNP density conditional on H3K4me3 enrichment and hotspot symmetry (*p* > 0.2). For *Prdm9*^*Hum*^-controlled NCOs, results show that H3K4me3 enrichment and hotspot symmetry have significant positive effects on NCOs conditional on SNP density (*p* < 0.003). Results from *Prdm9*^*Cast*^-controlled NCOs also suggest positive effects on prediction of NCOs, but p-values do not reach significance due to the smaller number of these events (<0.2). We discuss the weaker effect of symmetry for *Prdm9*^*Cast*^-controlled NCOs in Supplementary Note 8.

### Ethical compliance

All experiments involving research animals received local ethical review approval from the University of Oxford Animal Welfare and Ethical Review Body (Clinical Medicine board) and were carried out in accordance with the UK Home Office Animals (Scientific Procedures) Act 1986.

### Reporting summary

Further information on research design is available in the Nature Research Reporting Summary linked to this article.

## Supplementary information

Supplementary Information

Peer Review File

Description of Additional Supplementary Files

Supplementary Data 1

Supplementary Data 2

Supplementary Data 3

Supplementary Data 4

Supplementary Data 5

Reporting Summary

Source Data

## Data Availability

The mouse WGS sequencing data and variant calls are available under the SRA study accession SRP189007. The H3K4me3 ChIP-seq reads and peak calls are available under the GEO accession GSE119727. DMC1 ChIP-seq data^40^ were downloaded from GEO accession GSE124991. H3K9me3 ChIP-seq data^64^ from the broad-scale GLM analysis were downloaded from GEO accession GSE61613. The source data underlying Supplementary Tables 2 and 3 are provided as a Source Data file. Any additional data are available on request from the authors.

## Code availability

The computer code developed for the analysis of the datasets in the current study is available here: https://github.com/rosaranli/Detect_recombination_events_from_mouse_pedigree
