# Supplementary material for: A high-resolution map of non-crossover events reveals impacts of genetic diversity on mammalian meiotic recombination
Source: Nat Commun. 2019 Aug 29;10:3900. doi: 10.1038/s41467-019-11675-y (PMC6715734; doi:10.1038/s41467-019-11675-y)
Supplement: Supplementary file 1 — Supplementary Information [file 41467_2019_11675_MOESM1_ESM.pdf]

Supplementary Information

A high-resolution map of non-crossover events reveals impacts of genetic diversity on mammalian meiotic recombination

Ran Li†, Emmanuelle Bitoun†, Nicolas Altemose†, Robert W. Davies, Benjamin Davies, Simon R. Myers*

†contributed equally

*corresponding (myers@stats.ox.ac.uk)

Contents

p3 … **Supplementary Figure 1** Pedigree for all 279 mice bred in this study
p4 … **Supplementary Figure 2** Estimation of power to detect NCO events, and broad-scale patterns
p6 … **Supplementary Figure 3** Allelic dominance and broad-scale patterns
p7 … **Supplementary Figure 4** NCOs and COs distribute around PRDM9 binding motifs
p9 … **Supplementary Figure 5** GC-bias occurs independently of hotspot symmetry
p10 … **Supplementary Figure 6** Asymmetric hotspot properties
p12 … **Supplementary Figure 7** Recombination events avoid asymmetric hotspots
p14 … **Supplementary Figure 8** Comparison of two models for NCO repair

p16 … **Supplementary Table 1** Filters to identify true NCOs
p17 … **Supplementary Table 2** Joint distribution of COs and NCOs in F2 animals
p18 … **Supplementary Table 3** Raw correlations and GLM p-values examining broad-scale covariates of CO and NCO rates
p20 … **Supplementary Table 4** Summary of NCO/CO events overlapping DMC1 and/or H3K4me3 peaks
p21 … **Supplementary Table 5** GC-bias in NCO events
p22 … **Supplementary Table 6** P-values for the GLM analysis examining the effects of local SNP density on CO and NCO rates
p23 … **Supplementary Table 7** Primer sets used to validate a subset of NCO events by direct Sanger Sequencing of overlapping PCR amplicons

p24 … **Supplementary Note 1** Details of algorithm used to attribute *Prdm9* allelic control in hybrids
p25 … **Supplementary Note 2** Details of HMM algorithm used to identify CO and NCO events
p28 … **Supplementary Note 3** Details of filters to identify true NCOs
p30 … **Supplementary Note 4** GLM analysis of broad-scale predictors of CO and NCO rate, generating Supplementary Table 3
p32 … **Supplementary Note 5** Examining potential effects of SNP density on tract length estimation
p34 … **Supplementary Note 6** Details of algorithm used to estimate the number of autosomal DSBs in a single meiosis repairing using the homologue
p35 … **Supplementary Note 7** Further details of testing and characterizing the bias towards GC in NCO events
p45 … **Supplementary Note 8** Rejection sampling algorithm for COs and NCOs, construction of Fig. 5 and Supplementary Fig. 7, and testing for impacts of asymmetry on event resolution
p49 … **Supplementary References**

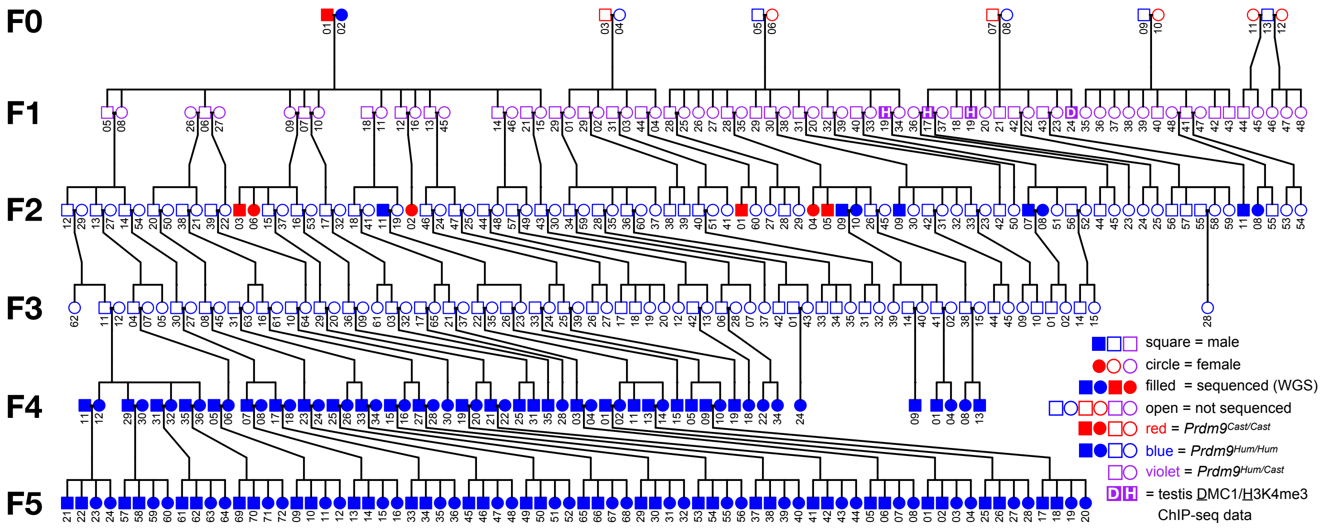

Supplementary Fig. 1 Pedigree for all 279 mice bred in this study. This illustrates the relationships of all sequenced and non-sequenced mice bred in this study, labelled with their IDs within each generation, and coloured by their PRDM9 alleles, with filled shapes representing mice that were sequenced (all information is listed in Supplementary Data 1). Note that to avoid line crossings, some mice are duplicated within a generation, e.g. appearing once in their litter and once in their breeding pair. This includes all *Prdm9*^{Hum} mice in F1-F4 that appear to have no offspring.

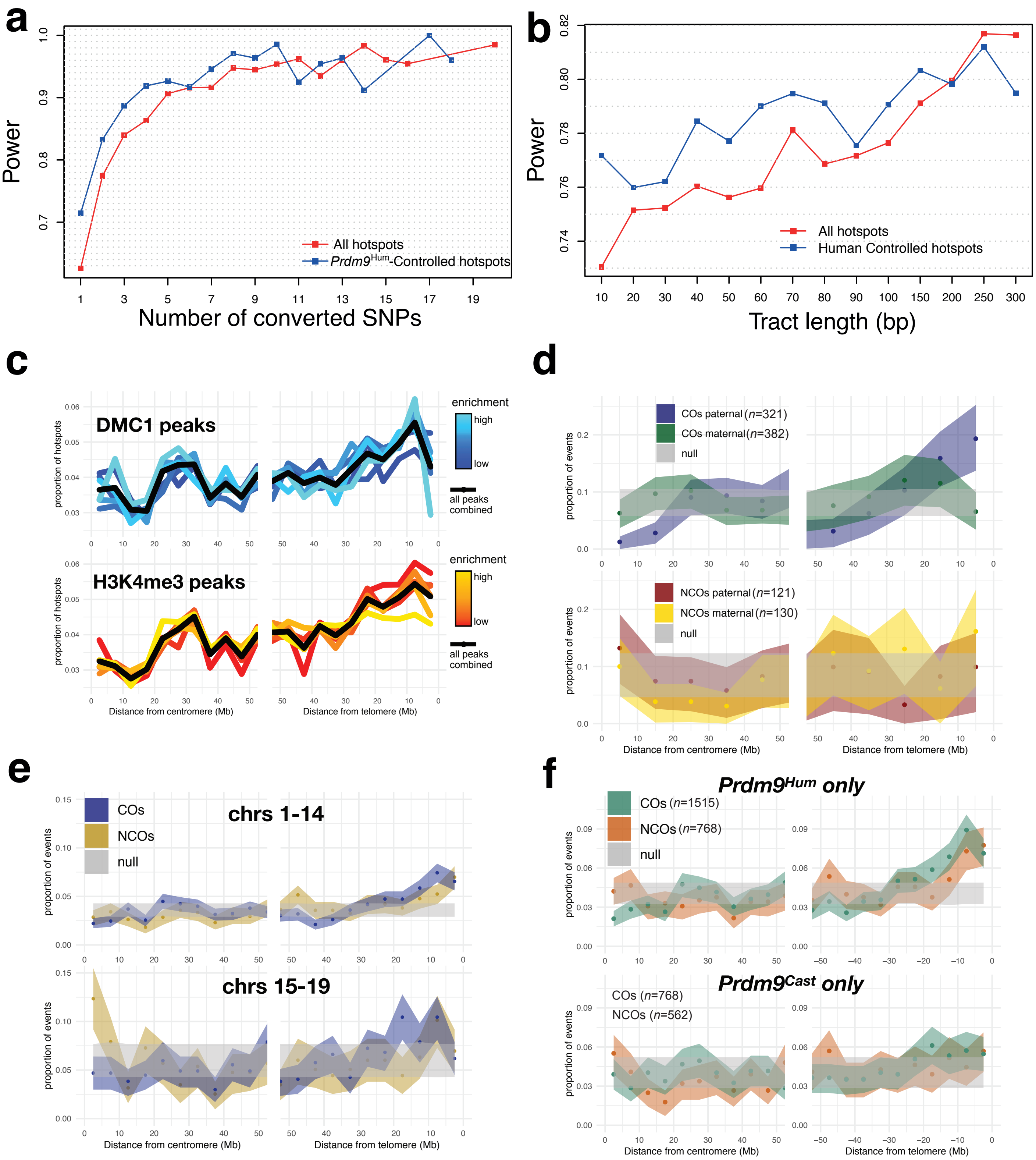

a
Power
Number of converted SNPs
All hotspots
Prdm9Hum-Controlled hotspots
b
Power
Tract length (bp)
All hotspots
Human Controlled hotspots
c
DMC1 peaks
H3K4me3 peaks
proportion of hotspots
enrichment
high
low
all peaks combined
Distance from centromere (Mb)
Distance from telomere (Mb)
d
COs paternal (n=321)
COs maternal (n=382)
null
NCOs paternal (n=121)
NCOs maternal (n=130)
proportion of events
Distance from centromere (Mb)
Distance from telomere (Mb)
e
COs
NCOs
null
chrs 1-14
chrs 15-19
proportion of events
Distance from centromere (Mb)
Distance from telomere (Mb)
f
Prdm9Hum only
COs (n=1515)
NCOs (n=768)
null
Prdm9Cast only
COs (n=768)
NCOs (n=562)
proportion of events
Distance from centromere (Mb)
Distance from telomere (Mb)

Supplementary Fig. 2 Estimation of power to detect NCO events, and broad-scale patterns. Simulations with increasing number of converted sites (**a)** or mean tract length (**b**). Red line: power to detect events in F2 mice, Blue line: power to detect human-controlled events in F5 mice. **c** Fraction of total DMC1/H3K4me3 enrichment coming from bins of genomic regions at different distances to the centromere or telomere (x-axis). DMC1 peaks or de novo H3K4me3 peaks were also split into sextiles by enrichment (~4000 peaks each for DMC1), and the profile for each sextile is plotted and coloured by enrichment. **d** as **Fig. 1e** but showing the distributions of the relatively few CO events (upper) and NCO events (lower) with known parental origin. **e** Plotting COs and NCOs across larger chromosomes 1-14 and smaller chromosomes 15-19 to show the difference in NCO enrichment at the centromere. **f** as (**e**) but showing the distributions of events controlled by each *Prdm9* allele separately.

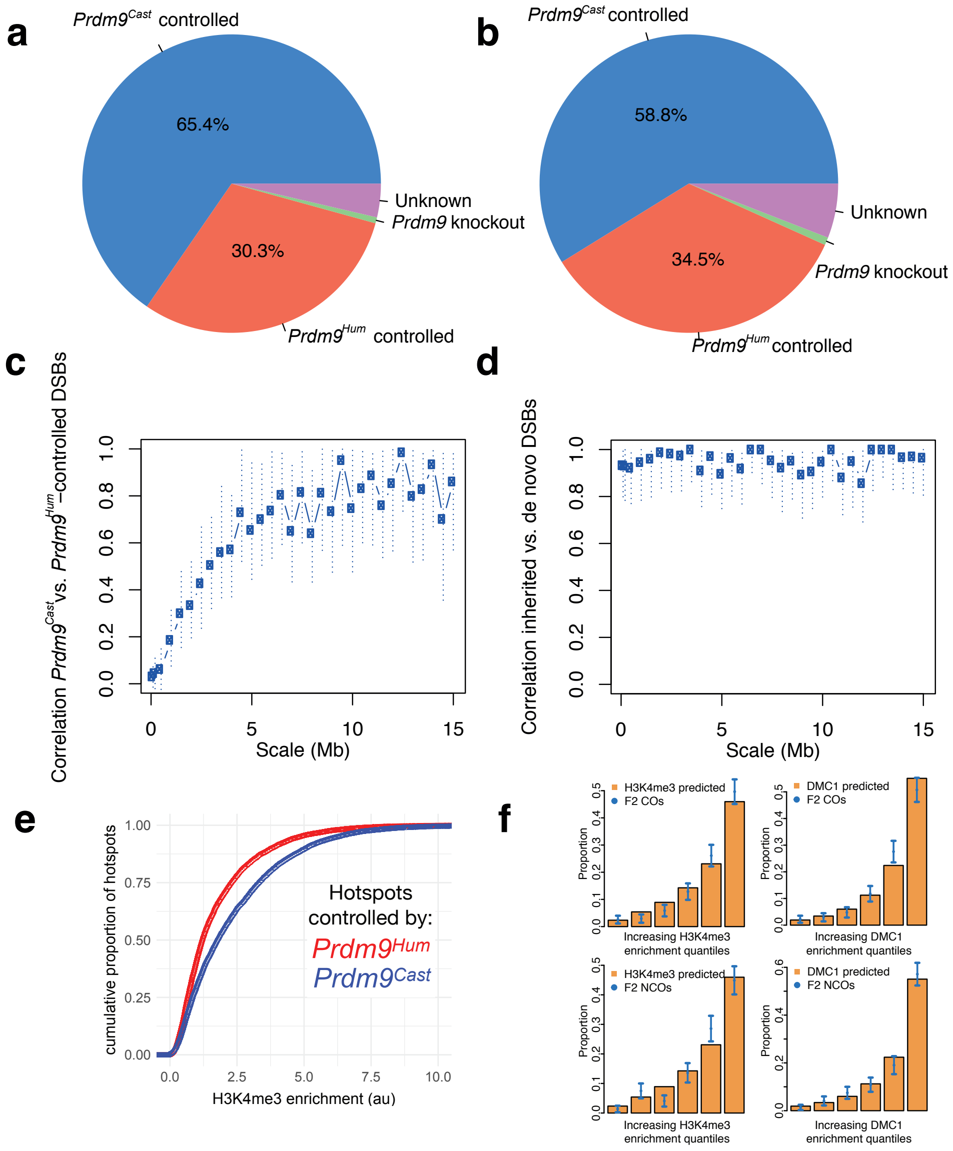

Supplementary Fig. 3 Allelic dominance and broad-scale patterns. DMC1 (**a**) and H3K4me3 (**b**) enrichment at DMC1 hotspots partitioned according to the controlling *Prdm9* allele. **c** Estimated underlying correlation between $Prdm9^{Cast}$-controlled recombination events and $Prdm9^{Hum}$-controlled recombination events at different scales. Details are as for Fig. 2g. **d** Correlation between inherited recombination events and *de novo* recombination events at different scales, as for **c**. **e** the empirical cumulative distribution functions for H3K4me3 enrichment at DMC1 hotspots controlled by each *Prdm*9 allele. **f** as Fig. 2b,c but separately for COs and NCOs.

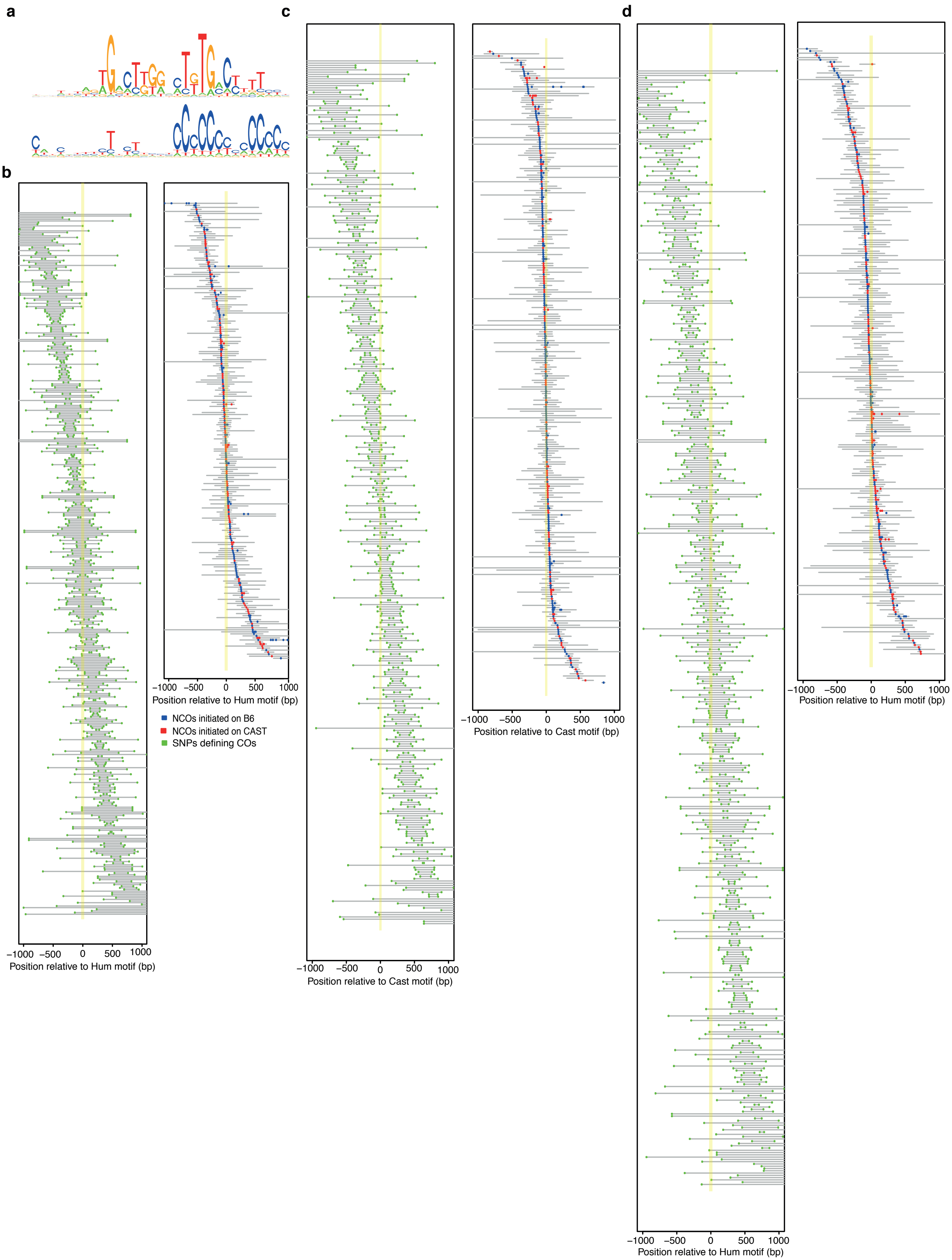
a
b
c
d
−1000 −500 0 500 1000
Position relative to Hum motif (bp)
NCOs initiated on B6
NCOs initiated on CAST
SNPs defining COs
Position relative to Cast motif (bp)

Supplementary Fig. 4 NCOs and COs distribute around PRDM9 binding motifs. **a** Distinct PRDM9 motifs identified, and their locations, within 97% of hotspots controlled by *Prdm9*^{Cast} (top) and 74% of *Prdm9*^{Hum} hotspots (bottom, only most informative bases shown). Distribution of F5 *de novo* (**b**) and inherited (**c-d**) COs (left) and NCOs (right) controlled by *Prdm9*^{Cast} (**c**) or *Prdm9*^{Hum} (**d**) around the PRDM9^{Hum} (**b, d**) or PRDM9^{Cast} (**c**) binding motifs. Sample sizes in the plots **b-d** from left to right are 427, 225, 385, 287, 490, and 276 respectively.

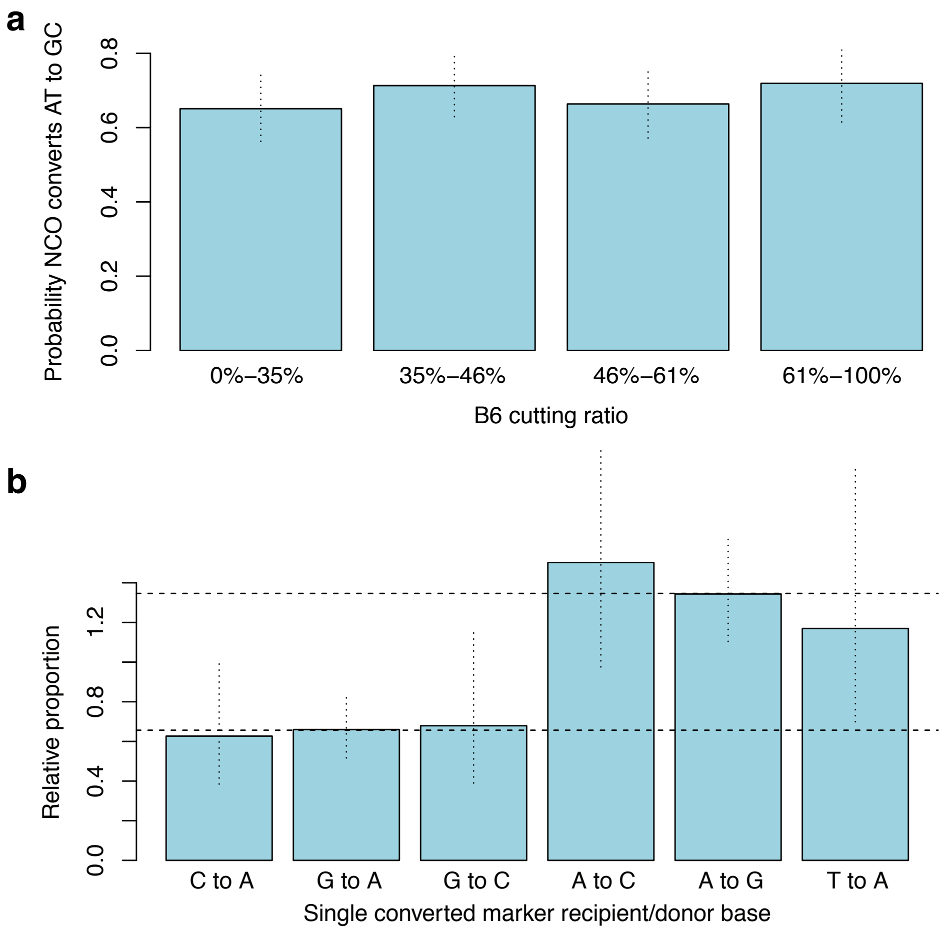

Supplementary Fig. 5 GC-bias occurs independently of hotspot symmetry. **a** GC-bias (y-axis) in hotspots with different fractions of reads coming from B6 (x-axis). **b** For each of the possible combinations of NCO donor/recipient alleles (x-axis; e.g. the first bar represents a Strong-to-Weak transversion SNP: recipient C converts to donor A, or G converts to T), the proportion of observed single-SNP NCOs of that type is plotted, relative to the corresponding proportion for all SNPs within observed multiple-SNP NCOs. Vertical lines: 95% CIs (binomial test). Horizontal dotted lines: mean relative proportions for NCO events whose recipient types are G/C or A/T respectively.

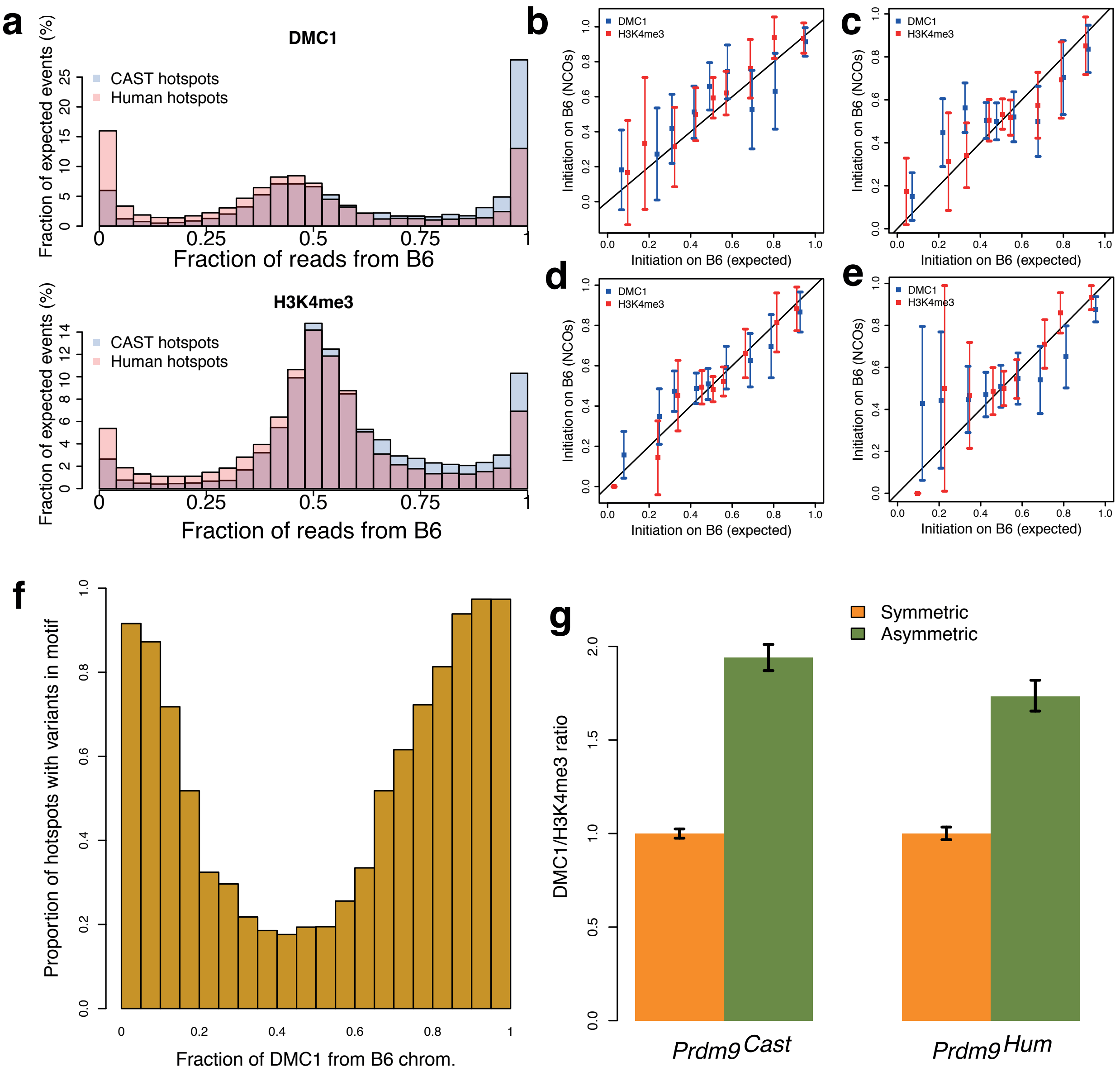
a
DMC1
CAST hotspots
Human hotspots
Fraction of expected events (%)
Fraction of reads from B6
H3K4me3
b
c
d
e
DMC1
H3K4me3
Initiation on B6 (NCOs)
Initiation on B6 (expected)
f
Proportion of hotspots with variants in motif
Fraction of DMC1 from B6 chrom.
g
Symmetric
Asymmetric
DMC1/H3K4me3 ratio
Prdm9 Cast
Prdm9 Hum

Supplementary Fig. 6 Asymmetric hotspot properties. **a** Proportion of DMC1/H3K3me3 reads coming from hotspots binned according to the bias in chromosome-informative reads towards the B6 chromosome (100%: all reads from B6). *Prdm9*^{Hum}-controlled and *Prdm9*^{Cast}-controlled hotspots are shown separately. **b**-**e** NCOs were binned according to their predicted (H3K4me3 or DMC1) B6 cutting ratio, and expected (x-axis) versus observed (y-axis) fraction of events initiating on B6 are plotted. Vertical lines: 95% CIs. Plots show events that overlap DMC1 hotspots that have defined symmetry estimates. They are from (**b**) F2 147 NCOs, (**c**) F5 *de novo* 319 NCOs, (**d**) F5 inherited 386 NCOs controlled by *Prdm9*^{Hum} and (**e**) F5 inherited 292 NCOs controlled by *Prdm9*^{Cast}. **f** For *Prdm9*^{Hum} and *Prdm9*^{Cast} hotspots binned according to the fraction of DMC1 reads from the B6 chromosome (x-axis), the fraction containing SNP/indel variants within the PRDM9 binding motif (y-axis). **g** Genome-wide autosomal ratio of mean DMC1 enrichment to mean H3K4me3 enrichment for asymmetric hotspots (fraction of reads from B6 chromosome is either larger than 0.95 or smaller than 0.05) relative to symmetric hotspots (fraction of reads from B6 chromosome is larger than 0.4 and smaller than 0.6) in both *Prdm9*^{Hum} and *Prdm9*^{Cast}-controlled hotspots. Error bars: 95% bootstrap CIs for the ratio of means.

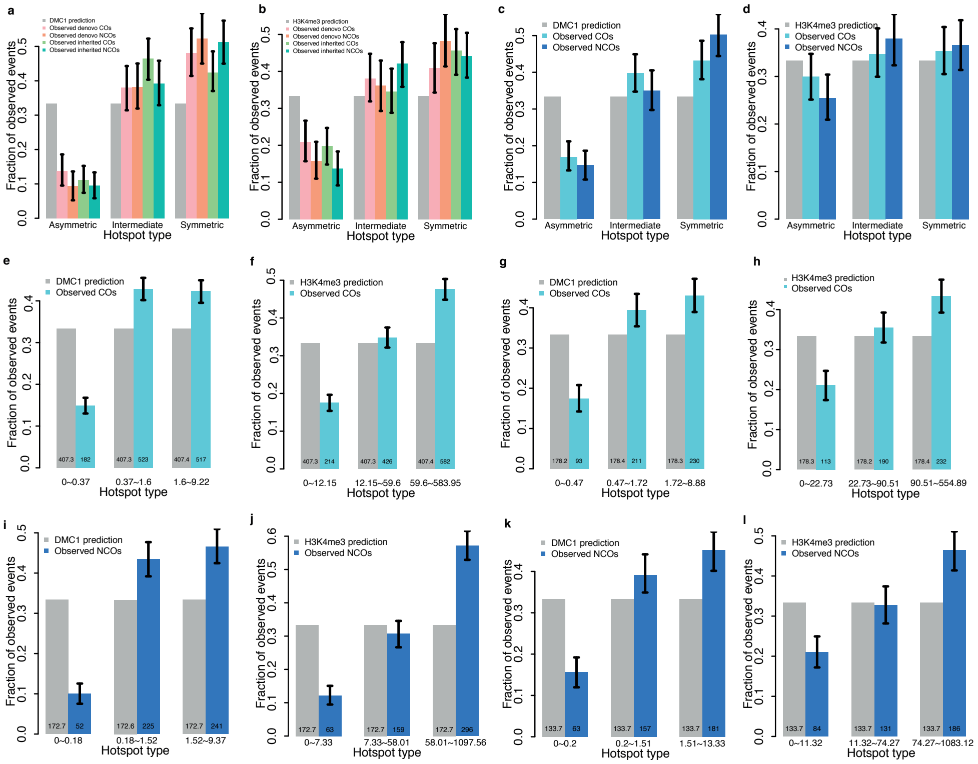

Supplementary Fig. 7 Recombination events avoid asymmetric hotspots. **a** *Prdm9*^{Hum}-controlled hotspots are binned by their symmetry (Supplementary Note 8) into asymmetric, intermediate, and symmetric hotspots, so each bin has the same expected number of events according to DMC1 enrichment. Grey bars: expected event fraction in bins from DMC1 enrichment. Coloured bars: observed number of (re-sampled) events in each bin, in labelled categories *de novo* COs from F5, *de novo* NCOs, inherited COs controlled by *Prdm9*^{Hum} and inherited NCOs controlled by *Prdm9*^{Hum}. Vertical lines: 95% bootstrapped confidence intervals. **b** As **a** except the binning and predicted events are calculated according to H3K4me3 enrichment. **c** As **a** but for recombination events controlled by *Prdm9*^{Cast}, so there are no *de novo* F5 events. **d** As **c** except the predicted events are calculated according to H3K4me3 enrichment. **e-h** As **a-d** for CO events (no rejection sampling), now binning hotspots according to their average enrichment on the homologous chromosome, and for labelled alleles and measures of hotspot heat

(DMC1/H3K4me3). **e-f** show COs controlled by *Prdm9*^{Hum} and **g-h** show COs controlled by *Prdm9*^{Cast}. **i-l** As **e-h**, except for (all) NCO events, and now binning hotspots according to their heats on the homologous chromosome (Supplementary Note 8), because the initiating chromosome is identifiable for NCOs. **i-j** show NCOs controlled by *Prdm9*^{Hum} and **k-l** show NCOs controlled by *Prdm9*^{Cast}.

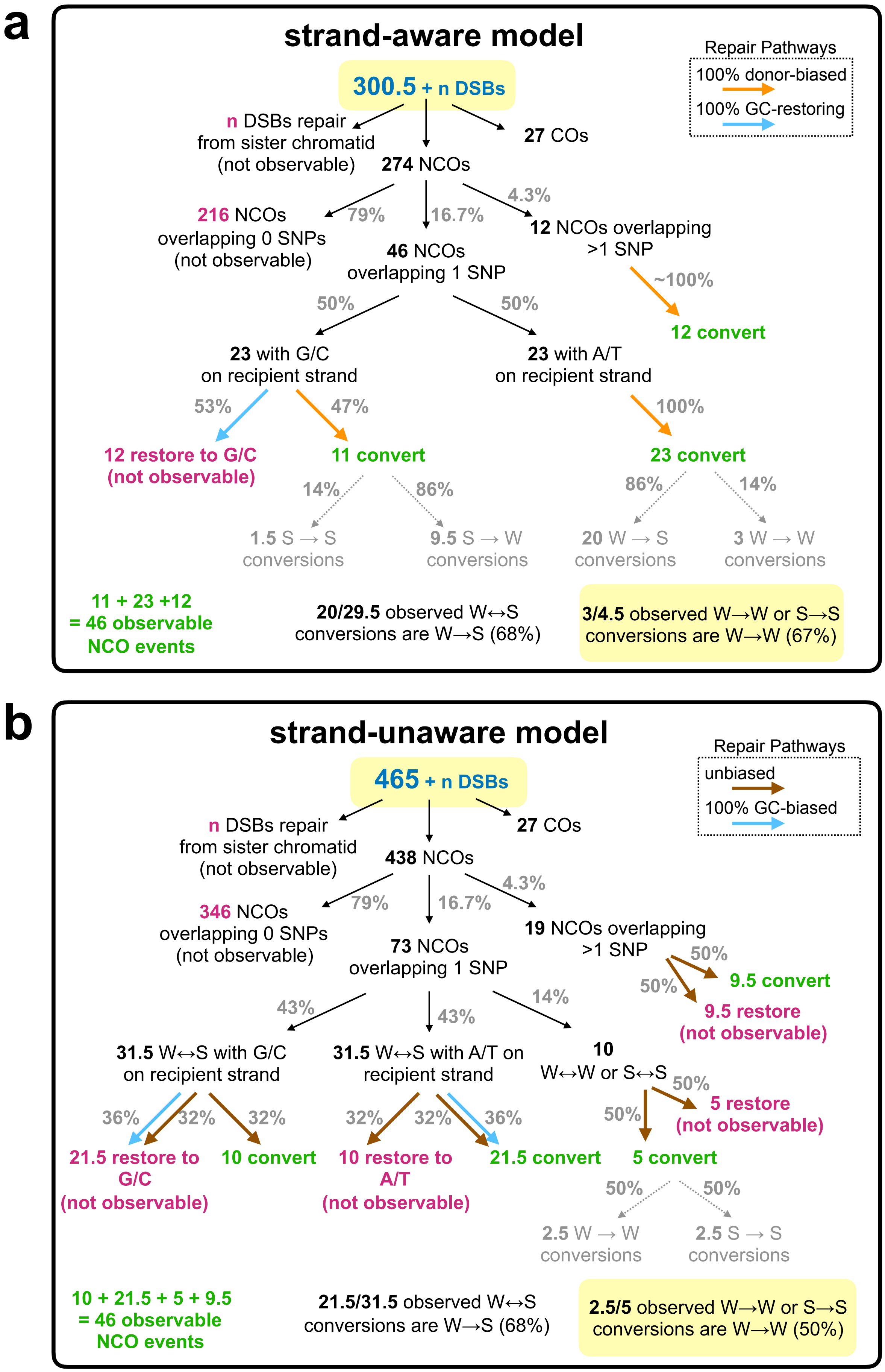
a
strand-aware model
Repair Pathways
100% donor-biased
100% GC-restoring
300.5 + n DSBs
n DSBs repair from sister chromatid (not observable)
27 COs
274 NCOs
79%
16.7%
4.3%
216 NCOs overlapping 0 SNPs (not observable)
46 NCOs overlapping 1 SNP
12 NCOs overlapping >1 SNP
~100%
12 convert
50%
50%
23 with G/C on recipient strand
23 with A/T on recipient strand
53%
47%
100%
12 restore to G/C (not observable)
11 convert
23 convert
14%
86%
86%
14%
1.5 S → S conversions
9.5 S → W conversions
20 W → S conversions
3 W → W conversions
11 + 23 +12 = 46 observable NCO events
20/29.5 observed W↔S conversions are W→S (68%)
3/4.5 observed W→W or S→S conversions are W→W (67%)
b
strand-unaware model
Repair Pathways
unbiased
100% GC-biased
465 + n DSBs
n DSBs repair from sister chromatid (not observable)
27 COs
438 NCOs
79%
16.7%
4.3%
346 NCOs overlapping 0 SNPs (not observable)
73 NCOs overlapping 1 SNP
19 NCOs overlapping >1 SNP
50%
50%
9.5 convert
9.5 restore (not observable)
43%
43%
14%
31.5 W↔S with G/C on recipient strand
31.5 W↔S with A/T on recipient strand
10 W↔W or S↔S
36%
32%
32%
32%
32%
36%
50%
50%
21.5 restore to G/C (not observable)
10 convert
10 restore to A/T (not observable)
21.5 convert
5 convert
5 restore (not observable)
50%
50%
2.5 W → W conversions
2.5 S → S conversions
10 + 21.5 + 5 + 9.5 = 46 observable NCO events
21.5/31.5 observed W↔S conversions are W→S (68%)
2.5/5 observed W→W or S→S conversions are W→W (50%)

Supplementary Fig. 8 Comparison of two models for NCO repair. The proportion of events along each arrow was determined empirically or by simulation, and we fit the models to explain at least the ~45 observable events we predict per meiosis given our detected events (see Supplementary Note 7). **a** The model proposed in this study, relying on a donor-biased pathway and a GC-restoring pathway. On average, only 12 potentially observable NCOs are restored and thus made not observable in each meiosis, requiring only ~300 DSBs to explain the observable ~46 events. We highlight this, and another important prediction: we expect W to W conversions to happen more often than expected compared to S to S conversions (our results are suggestive, though not conclusive, that this is true). **b** An alternative model that repairs heteroduplexes without any strand bias. This model "wastes" 46 potentially observable events and thus requires ~465 DSBs per meiosis to yield ~46 observable events, and it predicts W-W and S-S conversions should happen at the same rate.

Supplementary Table 1 Filters to identify true NCOs.

Filter	Description	Applied to
1	Removing sites that have read depth <20 in MGP version 4 data in B6 or CAST	All sites
2	Removing sites that have >1 alternative reads in B6 and sites that have >1 reference reads in CAST	All sites
3	Removing sites called heterozygous in any of the 28 strains of mice in the MGP data	All sites
4	Removing sites shared by >2 F2 animals	All sites
5	Removing sites such that within 500bp there are >28 reads whose mate pairs map (insert size) >1kb away	All sites
6	Removing sites covered by less than 10 good reads*	All sites
7	Removing sites that are called different by Platypus version 0.7.9.1	All sites
8	Removing sites that have read depth >95% quantile for the sample	Heterozygous
9	Removing sites that have <3 good reference reads or <3 good alternative reads	Heterozygous
10	Removing sites that show allelic imbalance (>70% of reads agree with non-converted background)	Heterozygous
11	Removing sites if they have genotype quality (GQ) <30	Heterozygous
12	Removing sites that have any good reads from the other allele	Homozygous
13	Removing sites where the nearby non-converted sites overlapping read pairs containing potential converted sites show allelic imbalance (potential allelic "dropout")	Homozygous
14	After applying the above filters, removing sites if there are >2 sites filtered within 500bp, and the fraction of removed sites in this region (<500 bp) is >50%. Iterate this process until we do not remove further sites ("guilt by association")	All sites
15	After step 14, recovering potential converted sites <1000bp from conversion events passing filters (avoid removal of genuine long or complex events by accidentally failing filters). Iterate until we do not recover additional sites.	All sites

* "Good" reads are defined as reads whose mate pair is not mapped to other chromosomes, and with insert size ≤1000bp. For reads containing the converted site, this site is >5bp from any indel and >10bp from the end of the read. Most properly mapped read pairs comfortably satisfy the first condition; we found empirically that alignment artefacts for reads failing the second condition led to many miscalled NCO events. See Supplementary Note 3 for further details.

Supplementary Table 2 Joint distribution of COs and NCOs in F2 animals.

F2 mouse ID	1 CO	1 NCO	2 CO	2 NCO	3 CO	3 NCO	4 CO	4 NCO	5 CO	5 NCO	6 CO	6 NCO	7 CO	7 NCO	8 CO	8 NCO	9 CO	9 NCO	10 CO	10 NCO
**F2_01**	1	1	2	0	0	0	1	1	2	2	2	2	2	0	1	3	1	1	2	0
**F2_02**	0	2	2	3	1	0	1	1	0	1	2	1	0	1	1	1	2	2	2	2
**F2_03**	2	3	1	1	1	0	2	1	2	2	2	2	1	0	0	0	1	2	2	1
**F2_04**	2	0	2	2	2	1	3	1	2	0	2	0	1	0	2	1	0	0	2	1
**F2_05**	3	2	3	1	1	2	1	1	1	2	1	0	2	0	1	2	2	1	2	4
**F2_06**	2	4	0	0	1	1	2	0	0	1	0	0	1	2	1	1	2	1	0	1
**F2_07**	2	0	3	1	1	1	2	1	1	2	2	0	1	0	2	0	3	2	2	0
**F2_08**	2	2	1	0	1	1	3	3	2	1	0	1	1	0	1	1	2	0	4	0
**F2_09**	1	1	4	4	1	2	1	0	3	1	3	0	3	1	2	0	1	1	1	0
**F2_10**	3	1	4	0	3	0	3	1	4	0	0	0	4	0	1	1	0	1	2	1
**F2_11**	0	0	1	5	1	1	3	1	4	1	2	2	3	0	4	2	2	1	1	1
**sum**	18	16	23	17	13	9	22	11	21	13	16	8	19	4	16	12	16	12	20	11
**sum (n=0)**	2	3	1	4	1	4	0	2	2	2	3	6	1	8	1	3	2	2	1	4

F2 mouse ID	11 CO	11 NCO	12 CO	12 NCO	13 CO	13 NCO	14 CO	14 NCO	15 CO	15 NCO	16 CO	16 NCO	17* CO	17* NCO	18 CO	18 NCO	19 CO	19 NCO	sum CO	sum NCO	sum CO=0	sum CO=0 & NCO>0
**F2_01**	2	1	1	1	1	2	1	0	1	1	0	1	1	2	0	0	1	0	22	18	3	1
**F2_02**	1	1	0	1	2	1	0	0	2	0	3	1	1	0	0	0	1	0	21	18	6	4
**F2_03**	3	1	1	2	2	3	2	1	2	1	1	1	1	0	2	0	1	1	29	22	1	0
**F2_04**	2	1	1	2	3	1	0	1	1	0	0	0	2	0	1	0	0	1	28	12	4	2
**F2_05**	4	1	2	0	1	3	0	0	3	0	2	0	1	1	0	0	0	0	30	20	3	0
**F2_06**	1	0	0	1	2	1	1	1	1	0	1	1	2	2	1	0	1	1	19	18	5	3
**F2_07**	2	1	1	1	2	1	1	1	1	0	1	0	1	1	2	1	1	2	31	15	0	0
**F2_08**	1	1	1	1	2	2	1	0	0	2	2	1	1	1	1	0	0	0	26	17	3	2
**F2_09**	0	0	0	0	1	0	0	0	1	0	1	1	1	0	0	1	0	2	24	14	5	2
**F2_10**	3	0	1	1	2	1	2	1	1	0	0	0	1	0	1	0	0	0	35	8	4	1
**F2_11**	3	0	1	0	1	1	3	1	0	1	0	1	0	0	1	1	0	2	30	21	4	3
**sum**	22	7	9	10	19	16	11	6	13	5	11	7	12	7	9	3	5	9	295	183	**38***	**18**
**sum (n=0)**	1	4	3	3	0	1	4	5	2	7	4	4	1	6	4	8	6	5				

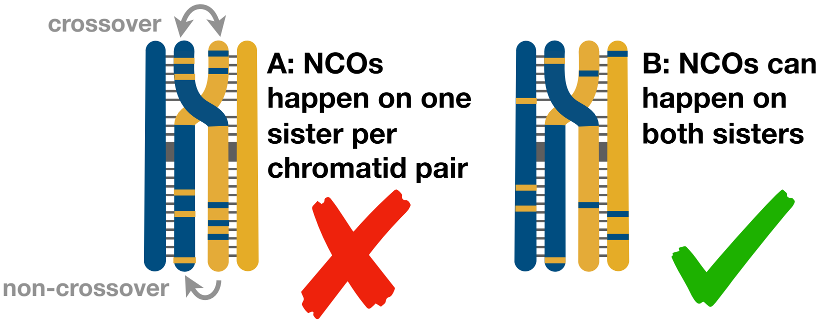

The number of COs and NCOs detected on each F2 chromosome are listed side by side. Here we focus on 38 inherited chromosomes that have 0 detected crossovers (*we exclude chr17 from counts as it contains *Prdm9*, which was selected to be homozygous in these mice). Given that we have full power to detect crossovers in F2 mice for much of the genome, when we observe an inherited chromosome pair with no crossovers, we are confident that in most cases it derived from two sister chromatids that did not participate in crossover recombination, one from each parent (obligate crossovers likely occurred on the other sisters). We were able to detect at least one NCO event on each of 18 of these 38 chromosomes, confirming that NCOs can occur on sister chromatids that do not participate in crossover. Source data are provided as a Source Data file.

Supplementary Table 3 Raw correlations and GLM p-values examining broad-scale covariates of CO and NCO rates.

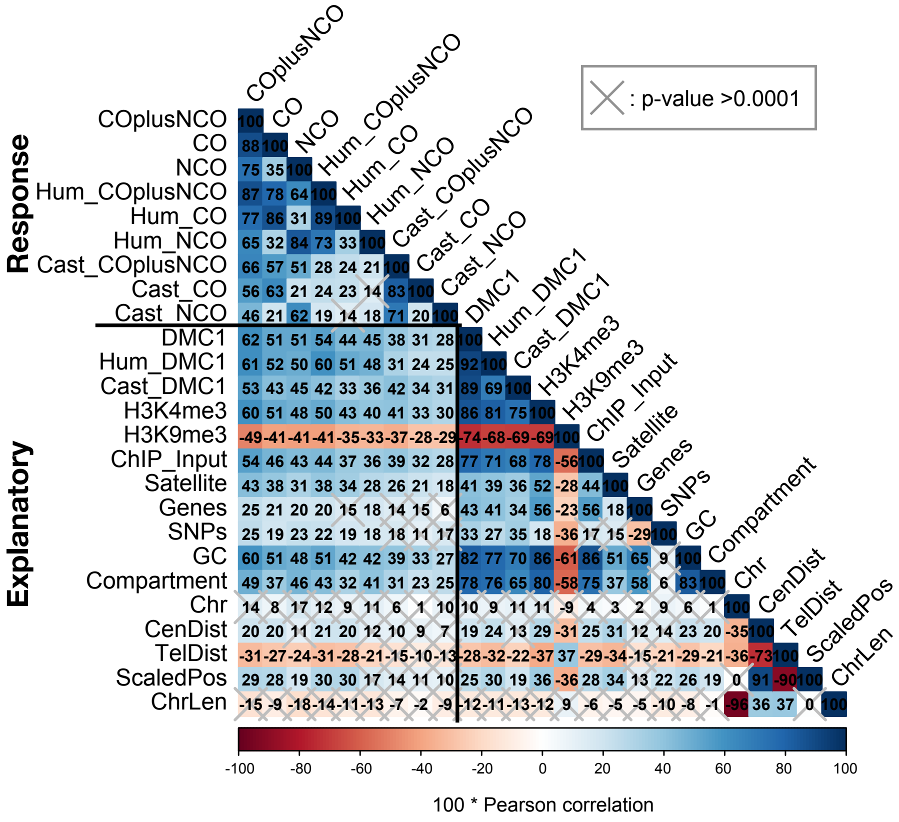

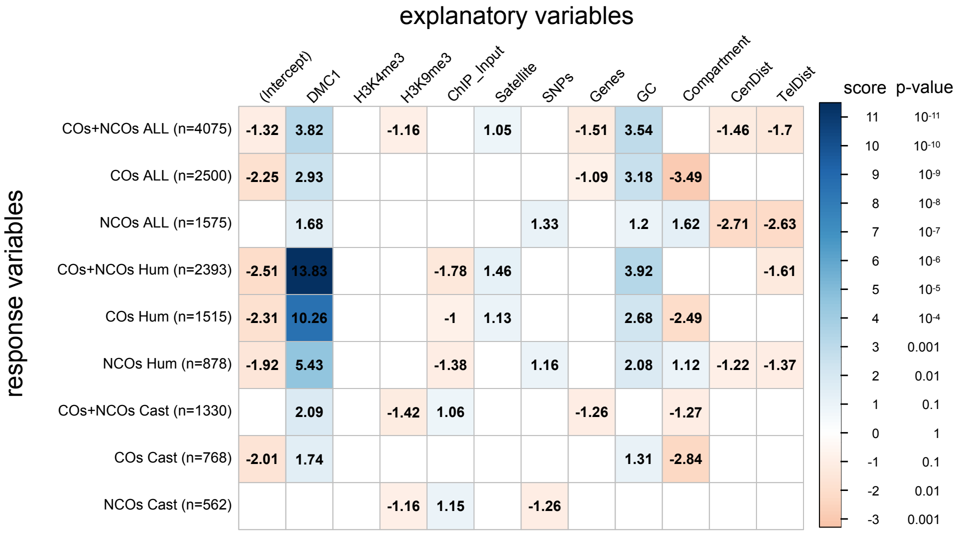

score = sign(coefficient) * -log$_{10}$(p-value)

p>0.1 not shown

Supplementary Table 3 continued. Upper table: A correlation matrix of CO and NCO counts and various explanatory variables (described in Supplementary Note 4). Correlations not significantly different than 0 (i.e. those with p>0.0001, correcting for multiple testing) are crossed out. **Lower table**: P-values from negative binomial Generalised Linear Models for each response variable using all explanatory variables (Supplementary Note 4). Shown are -$\log_{10}$ p-values for each coefficient, with the sign indicating the sign of the coefficient. For example, a value of -2 indicates a negative coefficient whose p-value is 0.01. P-values larger than 0.1 are not shown. (NB: for analyses of allele-specific events only the DMC1 peaks from the corresponding allele were used). Source data are provided as a Source Data file.

Supplementary Table 4 Summary of NCO/CO events overlapping DMC1 and/or H3K4me3 peaks.

Datasets	Total events	Overlap DMC1	Overlap H3K4me3	Overlap either	Overlap either (%)
F2 COs	295	272	271	282	95.6
F5 de-novo COs	821	646	690	728	88.7
Assigned F5 de-novo paternal COs	321	283	285	306	95.3
Assigned F5 de-novo maternal COs	382	269	310	323	84.6
F5 inherited COs	1384	1164	1196	1264	91.3
All COs	2500	2082	2157	2274	91.0
F2 NCOs	183	147	144	154	84.2
F5 de-novo NCOs	510	355	375	402	78.8
Assigned F5 de-novo paternal COs	121	98	98	103	85.1
Assigned F5 de-novo maternal COs	130	88	91	102	78.5
F5 inherited NCOs	882	730	713	771	87.4
All NCOs	1575	1232	1232	1327	84.3

Supplementary Table 5 GC-bias in NCO events.

NCOs		AT to GC	GC to AT	Probability of AT to GC	P value
Human controlled	All	554	366	0.60	6.2e-10
	F5 de-novo	261	160	0.62	1.0e-07
	F5 inherited	270	195	0.58	0.0006
CAST controlled	All	298	167	0.64	1.3e-09
	F5	218	111	0.66	3.7e-09
	F2	80	56	0.59	0.048
F5 de-novo	Paternal	71	53	0.57	0.1265
	Maternal	56	42	0.57	0.1888
	Either	127	95	0.57	0.0372

All events in this table overlap DMC1 hotspots. P-values are calculated via binomial two-sided tests.

Supplementary Table 6 P-values for the GLM analysis examining the effects of local SNP density on CO and NCO rates.

	SNP Window Size	SNP density	log(heat)	symmetry
**413 *Prdm9*^{Hum}-controlled COs**	±100bp	0.084	<2E-16	4.31E-08
	±500bp	0.137	<2E-16	6.84E-08
	±800bp	0.354	<2E-16	5.42E-08
**225 *Prdm9*^{Hum}-controlled NCOs**	±100bp	0.2898	2.64E-11	2.37E-03
	±500bp	0.54133	2.67E-11	2.61E-03
	±800bp	0.69627	2.81E-11	2.84E-03
**535 *Prdm9*^{Cst}-controlled COs**	±100bp	0.3687	<2E-16	0.0137
	±500bp	0.4026	<2E-16	0.0278
	±800bp	0.6818	<2E-16	2.78E-02
**401 *Prdm9*^{Cst}-controlled NCOs**	±100bp	0.934	<2E-16	0.165
	±500bp	0.354	<2E-16	0.215
	±800bp	0.731	<2E-16	1.75E-01

To do the analysis, we required hotspots to contain a motif in order to calculate the SNP density around motifs (6283 *Prdm9*^{Hum}-controlled hotspots and 10257 *Prdm9*^{Cast}-controlled hotspots). We used F5 de novo events that are controlled by *Prdm9*^{Hum} and all the events that are controlled by *Prdm9*^{Cast} so all the Hum events happen in the same generation (F5), just as all the Cast events happened in the same generation (F2). See Methods for details of the GLM analysis.

Supplementary Table 7 Primer sets used to validate a subset of NCO events by direct Sanger Sequencing of overlapping PCR amplicons.

PCR Amplicon (mm10 coordinates)	Size (bp)	Primer	Sequence (5'-3')
**NCOs outside hotspots**			
chr13:29183019-29183596	578	Forward	ACTGCCACCCTACAGAGCAT
		Reverse	GGCTCTCCCTGGAGAAAACT
chr17:51890148-51890704	557	Forward	GCCCAGTTCACAAGATGCAA
		Reverse	AGACCCACCTGTTCACACTT
chr5:3355452-3355968	517	Forward	ATAGCCTGAGAACTGCTTGGTC
		Reverse	GCGGTTGAGTTGGTAATGATTT
chr9:42122689-42123239	551	Forward	CTTTCTGGCTCCAGGAGTTG
		Reverse	AGGCTGGATTCTCTTCGTTG
chr18:71516706-71517158	453	Forward	CCACTGTCTCATTGCTTCCA
		Reverse	AGCTCTTGTTAAGGAGGCCA
chr6:58191294-58191839	546	Forward	TGTGTGTGTCTGAGAGAGTCA
		Reverse	GCTTTGGGGTTGATAGGTGC
chr6:62463907-62464499	593	Forward	ACAATGTGCCTTGGCTGTTT
		Reverse	CACATCAGTTAGTGGACATGGT
chr12:104530485-104531018	534	Forward	CCCAACTCCAGTGCATTCAG
		Reverse	CTGCCCTGCCTGACAAAAC
chr17:62918959-62919530	572	Forward	ACTGCTGGTAGGAATGCAAA
		Reverse	GGCAACACTGATCTTCGTCC
**NCOs within hotspots**			
chr2:179526483-179526944	462	Forward	CTTCCTAGGGCGGTCAGAAT
		Reverse	TGAGTGTCCAGAGCAGTTGT
chr12:111789470-111789979	510	Forward	GCGTAGAGTGGAATGGGGTA
		Reverse	AAAAGTCGGGAGCCCTGTC
chr14:55652534-55653053	520	Forward	CACGTAGGGTCAAAGGCTTT
		Reverse	TTCACTAGAGGGCTTGGCTT
chr19:36902497-36902984	488	Forward	AAGTGCTCCCTACTGCTGAG
		Reverse	GACCCTGCTCTGAGTCAAGT
chr11:100532613-100533209	597	Forward	CCTAGCCTCTTCTGGACAGG
		Reverse	GCCCATGGATCCAGAGGTAG
chr3:98052207-98052701	495	Forward	GCAGAGGGAAAGCAGCTAAC
		Reverse	CGCCCAGCTTTCTTGGATG
chr5:75789898-75790488	591	Forward	AGTGGGTCTTGTCTTCCAGG
		Reverse	CACAGTGTGGCAAGCTACTG
chr13:110018378-110018882	505	Forward	ACCAAATGCTCTATTGCTGTGT
		Reverse	CCCACCCTAACTCCAGACAA
chrX:16995858-16996443	586	Forward	GGAGCTGTGGGAAGATTTGC
		Reverse	GCACAGGGGAATCACAAAGG

Supplementary Note 1: Details of algorithm used to attribute *Prdm9* allelic control in hybrids

The *Prdm9* alleles in the hybrid mouse in this work are *Prdm9*^{Hum} and *Prdm9*^{Cast}. Using DSB maps from other samples (B6 [1], B6$^{B6/Hum}$, B6$^{Hum/Hum}$, B6$^{-/-}$ [1], B6xCAST, PWD, B6xCAST$^{Hum/Cast}$, (B6xCAST)F2$^{B6/Hum}$, PWDxB6, B6xPWD, PWDxB6$^{Hum/PWD}$, B6xPWD$^{Hum/PWD}$), we were able to classify DSB hotspots as being under the control of either the humanized or CAST *Prdm9* allele in the hybrids. We thank Anjali Hinch for suggesting the following procedure.

We create a superset of hotspots, which are combined across mice such that hotspots that have their centres within 600 bp of each other are considered to represent the same hotspot. For each hotspot in this superset, we then create a maximal set of *Prdm9* variants that could potentially be responsible for activating it. For example, if a hotspot overlaps hotspots in B6xCAST and B6xCAST$^{Hum/Cast}$, the potential set of alleles that could activate the hotspot is *Prdm9*^{B6}, *Prdm9*^{Cast}, and *Prdm9*^{Hum}. From the maximal set, we reduce to a minimal set of alleles that can explain all of the mice in the set. In the example above it is *Prdm9*^{Cast}. Hotspots for which the minimal set consists of a single *Prdm9* variant are inferred to be activated by it. There are two special cases: Hotspots in the B6$^{-/-}$ are said to arise from a dummy allele *Prdm9*^{KO}. Hotspots that overlap with these hotspots are assigned the allele "KO". *Prdm9*^{Cast} and *Prdm9*^{PWD} have similarities in their zinc finger arrays and a large number of overlapping hotspots. If the maximal set contains more than one of these variants, we treat them as equivalent. It is not always possible to reduce the minimal set to a single *Prdm9* variant. For example, if a hotspot is found in B6xCAST, B6xCAST$^{Hum/Cast}$ and B6^{Hum}, then no single *Prdm9* variant can explain all the hotspots. The maximal set cannot be reduced from *Prdm9*^{B6},

Prdm9^{Cast} and *Prdm9*^{Hum}. In this case, we take the following approach to assign alleles in the B6xCAST$^{Hum/Cast}$ mouse that is of interest in this work:

1. For hotspots where the minimal set contains both *Prdm9*^{Cast} and *Prdm9*^{Hum}, we say the allelic type is "*unknown*" or "*MULT*".

2. For hotspots where the minimal set contains *Prdm9*^{Cast}, but not *Prdm9*^{Hum}, the allelic type is "*CAST*".

3. For hotspots where the minimal set contains *Prdm9*^{Hum}, but not *Prdm9*^{Cast}, the allelic type is "*HUM*".

Supplementary Note 2: Details of HMM algorithm used to identify CO and NCO events

Using the information from the filtered strain-informative SNPs, we developed a Hidden Markov Model (HMM) to infer the strain origin of each broad segment of the genome. In our HMM, the three possible emitted genotype states B6/B6, B6/CAST and CAST/CAST are represented by 0, 1 and 2, respectively (i.e. the number of CAST allele copies at each strain-informative SNP site). Similarly, the hidden states representing background strain origin are encoded as 0, 1 and 2 copies of a CAST haplotype. Emitted states may be different from hidden states due to sequencing errors or real converted events (e.g. observing a homozygous CAST genotype on an otherwise heterozygous CAST/B6 background). A natural initial stationary distribution is (0.25, 0.5, 0.25) corresponding to state triple (0, 1, 2). The state transition between two sites is driven by recombination events, with the distance between two different states following an exponential distribution with a rate parameter equal to twice the

recombination rate. Here we adopted a genome-wide average constant recombination rate of r=0.625x10^{-8} per base pair per generation[2,3]. Thus, the probability of recombination from site i to site j can be written as follows:

$$P_{ij}=1-\exp(-2rD_{ij}), \qquad (1)$$

where P_{ij} and D_{ij} stand for the recombination probability and distance between site i and j, respectively. The transition probability matrix from site i to site j is as follows:

$$\mathbf{P}_{ij}=(1-P_{ij})\mathbf{I_3}+P_{ij}\mathbf{Q}, \qquad (2)$$

where I_3 is the 3×3 identity matrix and Q stands for the conditional transition matrix with the entry q_{mn} (m=0,1,2; n=0,1,2) describing the transition probability from state m to state n:

$$\mathbf{Q}=\begin{bmatrix} 0 & 1 & 0 \\ 1/2 & 0 & 1/2 \\ 0 & 1 & 0 \end{bmatrix}. \qquad (3)$$

There is no transition from state 0 to state 2, or vice versa, because it's unlikely that two independent recombination events would happen at exactly the same position with a small sample size. Conditional on there being a recombination event, state 0 or state 2 transitions to state 1 with probability 1, and state 1 transitions to either state 0 or state 2 with equal probability.

Here we defined the emission probabilities from each hidden state by using the quality metrics from GATK for states 0, 1 and 2. Given state g in each site t, GATK provides a quality score S for three states as follows:

$$s_g^t = -10\log_{10}\frac{\mathrm{p}(D|G_t=g)}{\max\limits_{k=0,1,2}\mathrm{p}(D|G_t=k)}, \qquad (4)$$

where $\mathrm{p}(D|G_t = g)$ is the probability that we observe the data D, conditional on the hidden state G_t being g. Since for each site t, the maximum score is constant, we can inversely infer the probability of observing different states with a constant scale factor:

$$\mathrm{p}(D|G_t = g) \propto 10^{-\frac{s_g}{10}}. \quad (5)$$

In our analysis, the scaling parameter was arbitrarily set to 1.

We applied the forward-backward algorithm to infer the posterior distribution of hidden states. Starting with prior state probabilities (0.25, 0.5, 0.25) at the first site, the forward probability of state j after seeing the first t sites is

$$\alpha_t(j) = \sum_{i=0}^{2} \alpha_{t-1}(i) p_{ij}(t-1) e_j(t), \quad (6)$$

where $p_{ij}(t\text{-}1)$ is the $(i,j)^{\text{th}}$ element of transition matrix **P** at site t-1, and $e_j(t)$= $\mathrm{p}(D|G_t = j)$is the emission probability conditioned on state j at site t given by equation 5. At the same time, we define a backward chain with an initialised probability (1, 1, 1) at the end of the site using the following:

$$\beta_t(j) = \sum_{k=0}^{2} \beta_{t+1}(k) p_{jk}(t) e_j(t+1), \quad (7)$$

and the probability of hidden state j, given the observed data (j=0,1,2) at site t is

$$\mathrm{p}_t(j) = \frac{\alpha_t(j)\beta_t(j)}{\sum_{i=0}^{2} \alpha_t(i)\beta_t(i)}. \quad (8)$$

Finally, we can calculate the stationary distribution of states 0, 1 and 2 for each strain-informative SNP site given the sequencing data, and for each site we choose the hidden state with maximum probability as the real strain background state at that site. Finally, because we wish to compare genotypes to this background state to identify NCO events (using additional filters), we smoothed the resulting initial background estimation, by reverting inferred changes in background spanning <50 SNPs to the broader inferred background state (such changes were tested as potential NCO events, instead). So changes spanning <50 SNPs are defined as NCOs and otherwise they are defined as two consecutive COs. Actually, the maximal number of SNPs we see from a NCO is only 9 SNPs, indicating that NCOs are quite distinguishable from double COs. A

candidate NCO can be either within 1000 bp of a CO or 20 kb away from a CO. If an NCO is near (<1000 bp) to a CO and we have evidence to show they are generated from the same generation (both of them are de-novo or always appear together if inherited), it is defined as a complex CO.

Supplementary Note 3: Details of filters to identify NCOs

For the potential de-novo NCOs that we identified from F2 and F5, we applied a series of filters to remove false positives (Supplementary Table 1). While we use many standard filters, our thresholds are necessarily chosen in a somewhat *ad hoc* way; we verify accuracy of resulting NCO events using direct Sanger sequencing of selected events, and high DMC1/H3K4me3 hotspot overlap. We aimed to construct filters to reduce the SNP genotyping error rate to very low values (of the order of 10^{-7}), necessary to call NCOs, and we estimated our resulting power to identify events via simulations incorporating these filters (Methods). Firstly, we removed sites (across our datasets) that are potentially heterozygous, or which appear heterozygous, in the founders, as this violates an underlying assumption of our event calling. Specifically we remove sites that either have read depth <20 in MGP version 4 data in B6 or CAST; have >1 alternative allele reads in B6 or >1 reference allele reads in CAST; or are genotyped as heterozygous in any of the 28 strains of mice in the MGP data.

Secondly, we identified the set of potential NCO sites (whose genotypes do not match their local background) across individual animals, and filtered out potential genotyping errors among them by setting these site genotypes to missing in the animal concerned. We filtered out sites

(i) whose genotypes are called differently by Platypus version 0.7.9.1

(ii) with fewer than 10 good reads (defined as reads whose mate pair is not mapped to other chromosomes, and with insert size ≤1000 bp. For reads containing the converted site, this site is >5 bp from any indel and >10 bp from the end of the read.)

(iii) if they were called heterozygous but showed low genotype quality GQ<30, allelic imbalance (<30% of reads from one of the alleles, potentially miscalled homozygous sites), possessed insufficient reads (<3) for either of the alleles (potentially miscalled homozygous sites), or had read coverage above the 95th percentile for the sample (which may indicate duplicated regions).

(iv) if they were called homozygous but showed low coverage (<10 reads) or have >0 good reads for the alternative allele (as potentially heterozygous sites). To avoid filtering out a site, we required read pairs overlapping the site as well as a heterozygous non-converted site, and sampling *both* alleles of the heterozygous site. (Failing this condition potentially indicates heterozygous sites where, by chance, reads from only one allele are sampled: potential allelic "dropout".)

(v) whose genotypes do not match their background in two or more F2 animals, as likely genotyping errors, reasoning that the chance of two identical NCO events occurring at the same location in only 11 animals is small (by resampling simulated events according to their DMC1 enrichment, we estimate this would only produce 3 false negatives, while removing ~100,000 false positives).

(vi) such that within the 500 bp surrounding the site, there are >28 reads whose mate-pair maps > 1 kb away, indicating an unusually large insert size,

because such regions may be explained by mismapping of reads, or polymorphic large indels near the SNP concerned. This threshold was chosen empirically to remove outliers given the distributions of coverage and insert sizes in the dataset.

After applying the above filters, we removed sites within individuals if there were >2 sites filtered within 500 bp and if the fraction of removed sites in this region against all sites (<500 bp) is >50% ("guilt by association", aiming to identify and filter regions of poor genotyping quality). Because this further increases the number of removed sites, we iterated this process until it reached stationarity.

Having applied the above filters, we called NCO sites as positions passing all these filters and not matching their local background. Following this, because we expect e.g. our coverage and mate-pair filters to filter out some genuine genotype calls through chance, we recovered sites <1000 bp from identified conversion events, even if they failed the above filters, and again iterated to stationarity. This process allows us to avoid artificially truncating NCO events, e.g. long or complex events.

Supplementary Note 4: GLM analysis of broad-scale predictors of CO and NCO rate, generating Supplementary Table 3

The mm10 reference genome was split into 472 non-overlapping 5-Mb bins, excluding centromere and telomere gaps and removing shorter bins adjacent to telomeres. Total CO and NCO counts were tallied up in each bin, and separate tallies were made for events that were attributed to the *Prdm9*^{Hum} or *Prdm9*^{Cast} alleles. Explanatory variables included DMC1 ChIP-seq peaks in each bin, and separately for peaks assigned to each

Prdm9 allele (NB: for analyses of allele-specific events only the DMC1 peaks from the corresponding allele were used). We included the total number of PRDM9-dependent H3K4me3 ChIP-seq peaks; H3K9me3 ChIP-seq peaks (using data from GSE61613)[4]; total chromatin input DNA from our H3K4me3 ChIP-seq experiment, as a measure of accessibility and sequencing bias; number of bases annotated as mouse major and minor satellites from mm10; number of Refseq genes; number of B6/CAST SNPs from this study; proportion of GC bases; the HiC chromatin compartment assignment score used in two previous studies[5,6] (positive scores for gene-rich compartment A, negative scores for gene-poor compartment B); chromosome number, which roughly reflects its rank by length (1-19); distance of the bin midpoint from the edge of the centromere gap (bp); distance of the bin midpoint from the edge of the telomere gap (bp); the position of the bin midpoint as a fraction of the chromosome length; and the chromosome length in bp. The input data are available in the Source Data file.

We fit negative binomial Generalised Linear Models with log link functions for each response variable using all explanatory variables (except ScaledPos, Chr, and Chr Length, as these are simple linear transformations of CenDist plus TelDist). The response data fit a negative binomial distribution closely, and residual deviances were close to the number of degrees of freedom, indicating a lack of substantial overdispersion. We also generated submodels and found that the coefficients for Compartment and Genes become negative when GC is added to the model, implying that the positive correlations of these variables with recombination rates are attributable primarily to their greater GC content.

Supplementary Note 5: Examining potential effects of SNP density on tract length estimation

We observe 58% more SNPs within 200 bp of PRDM9^{Cast} binding sites relative to PRDM9^{Hum} binding sites on average, likely owing to hotspot erosion, increased mutation, and gBGC on the CAST background (NB: there is a 164% increase in SNP density within binding motifs themselves, diminishing to a 4.9% increase 2kb away). Given the differences in SNP density surrounding the alleles' binding sites we did not simply take an average of minimal/maximal conversion tract lengths across all sites, which would likely yield smaller estimates for the allele with more nearby SNPs. Instead, we fit an exponential model based on the empirical observation of co-conversion of alleles conditional on their distance from each other (see Figure 2h and Methods). This conditioning should account for the difference in SNP density, with greater SNP density only improving the precision of estimates at the lower end of inter-SNP distance, but not biasing the overall trend of the data across different distances. The precision of this model fitting depends both on SNP density and the number of events used. In fact, because we were able to fit the model for the Humanized allele using both F2 and inherited/de novo F5 events, we actually had greater overall power to estimate tract length for the Humanized allele (using 815 total events overlapping hotspots), even at small length scales, compared to the Cast allele (for which we only have 409 F2 and inherited F5 events overlapping hotspots), but the Cast allele still showed a significantly shorter mean tract length.

To further confirm that tract length estimation is robust to SNP density, we performed simulations by removing 5%-30% of SNPs (with step size 5%) within 1500 bp of all NCOs overlapping *Prdm9*^{Cast}-controlled hotspots (as these have greater overall SNP

density for subsampling), and we repeated tract length estimation again. The resulting mean tract length estimates are very similar to previous estimates and do not show an obvious association with SNP density: 29.52, 30.55, 29.07, 28.42, 29.70, 31.40, respectively (vs 30 bp when using all SNPs). While *inference* of mean tract length does not appear to depend on SNP density, we wondered if SNP density might affect *actual* tract lengths by some molecular mechanism. For example, perhaps high local SNP density can limit the length of gene conversion tracts by some mismatch detecting mechanism. Alternatively, because regions with lower SNP density are expected to show more overall gBGC (as illustrated in Figure 4b) and as a result are hypothesized to have a lower gene conversion rate due to the action of the 'GC-restoring' pathway (explained in Figure 6 and in Supplementary Note 7 below), then perhaps the longer observed tract lengths at the relatively SNP-poor $Prdm9^{Hum}$-controlled hotspots reflect the depletion of single-SNP S to W conversions. That is, because short tracts are more likely to contain single S/W SNPs that fail to convert, long tracts overlapping multiple SNPs are expected to become overrepresented, with a greater effect size at relatively SNP-poor $Prdm9^{Hum}$-controlled hotspots compared to SNP-rich $Prdm9^{Cast}$-controlled hotspots. This phenomenon would be expected to be amplified by the fact that $Prdm9^{Hum}$ binding sites are more G/C rich than $Prdm9^{Cast}$ binding sites. The magnitude of these effects would likely be modest, but they could contribute to the observed difference in tract lengths.

To examine these possibilities, we separated converted SNPs from $Prdm9^{Hum}$-controlled hotspots (as these have not co-evolved with the genome) into two subsets: 349 SNPs in "low-density" hotspots with fewer than 3 SNPs in the central 200 bp surrounding the motif, and 494 SNPs in "high-density" hotspots with 3 or more SNPs.

Performing tract length estimation in each subset yielded mean estimates of 37 and 35 bp, which are not significantly different (bootstrap p-value 0.736; NB: slightly shorter estimates are obtained when using only events in motif-containing hotspots vs 41 bp when using all events). This indicates that neither hypothesis is likely to explain the large difference in tract length observed between *Prdm9*^{Hum} and *Prdm9*^{Cast}–controlled events, though we lack power to rule out smaller effects.

Supplementary Note 6: Details of algorithm used to estimate the number of autosomal DSBs in a single meiosis repairing using the homologue

We assume that the average number of DSBs per meiosis resolving as NCO events is K. Because each NCO affects only one of four chromatids, only one quarter of them will be seen in a single offspring.

We take F2 animals as an example; an identical approach was used for F5 events. Twenty-two meioses occurred, to generate 11 F2 animals. If *D* is SNP density near DSBs, *L* is average NCO tract length, and "Power" represents the power we have to detect a particular SNP within a NCO event, then if *N* is the number of converted sites observed, we have:

$$\mathrm{E}(N) = \frac{K}{4} * 22 * \mathrm{Power} * L * D \qquad (9)$$

Values for *N*, *L*, "Power" and *D* together allow estimation of *K*. We observe 0.0072 SNPs per bp within hotspots, and *N*=240 distinct converted sites in total; moreover, we estimate tract length *L*=30, and a power of 74.3% for these animals. This yields an estimate of $\hat{K} = 274$ DSBs resolving as NCO events, per meiosis.

For CO events, we have near 100% power to observe these, and half of all recombination CO events are transmitted to a particular offspring. Therefore, based on 295 observed CO events in these mice, the (sex-averaged) estimated number of CO events is 295x2/22=26.8 per meiosis.

The sum of these numbers is the total number of autosomal events repairing using the homologous chromosome, per meiosis (we neglect the X-chromosome in this calculation). To obtain CIs for the number of NCOs, COs and the total number of recombination events per meiosis and for the NCO to CO ratio, we performed bootstrapping as to estimate the tract length of NCOs. For each bootstrapped sample (of 10,000), we obtained the number of NCOs and number of COs, and used these to re-estimate the total number of recombination events and the NCO/CO ratio.

Supplementary Note 7: Further details of testing and characterizing the bias towards GC in NCO events

To test for the presence of GC-bias in NCO events, we first combined inherited and *de novo* NCO events inferred to be under *Prdm9*^{Hum} control, and occurring within (<1 kb from) DSB hotspots identified using our DMC1 data. This identified a total of 1,011 SNPs within such NCO tracts. We focus only on *Prdm9*^{Hum}-controlled events because the resulting hotspots are newly introduced, and so unlike *Prdm9*^{Cast}-controlled DSB hotspots cannot have been influenced by historical recombination, e.g. generating an excess of mutations towards G/C carried on the CAST genome.

Initially, we simply tested for an overall GC-bias by testing for an excess of SNPs converted from A/T towards G/C vs. the converse direction, yielding strong evidence

(59.9% towards GC, $p=3.7\times10^{-9}$ by 2-sided binomial test). This bias occurs despite the great majority of our detected NCO events (encompassing 99% of all converted SNPs) representing simple, contiguous converted tracts. This raises the question of whether multiple co-converted SNPs still show a GC bias.

We therefore separately tested for directional GC bias of converted SNPs where the adjacent SNPs in our set (among the 14,334,181 genome-wide) were either both non-converted, or where at least one of the adjacent SNPs was converted. These represent conversion of a single isolated SNP, vs. SNPs falling within a multiple-SNP tract, respectively. We observed a strong conversion bias for isolated SNPs (68.1% towards GC, $p=1.9\times10^{-15}$). In contrast there is no evidence of bias whatsoever for SNPs within multiple-SNP tracts (50.4% towards GC, $p=0.921$). This difference is highly significant ($p=1.1\times10^{-7}$, by Fisher's Exact Test). Thus, GC-biased gene conversion appears to effectively exclusively influence the shortest conversion tracts, of single SNPs. Below, we analyse occasional complex events that are observed.

We separated NCO events into bins according to sex, underlying hotspot heat and hotspot symmetry, and NCO position relative to the PRDM9 binding motif (Supplementary Data 5 and Supplementary Fig. 5): no differences in either the lack of bias in multiple-SNP tracts, or the quantitative GC-bias of single-SNP NCO events, were observed. For symmetry, we split single-SNP NCO events into four quartiles based on their proportion of DMC1 signal coming from the B6 chromosome, ignoring SNPs within 20 bp of the centre of an identified (humanized) PRDM9 binding motif because such SNPs might be involved in driving (a)symmetry itself. No impact of symmetry on the bias is seen, so GC-bias is not driven by DSB initiation bias.

We also tested whether humans showed similar, or different, properties to those we identified in mice genome-wide. We downloaded the Supplementary Data file from Halldorsson et al. 2016, by far the largest genome-wide NCO study yet conducted in humans[7]. Despite this, data were more limited than in our mice, due to the much lower SNP density in humans (at least 5-fold), together with the fact that only NCO events identified using sequencing can be verified as single- vs. multi-SNP events. Specifically, we analysed all SNPs occurring within verified NCO events, identified using sequencing. Individual NCO events were then separated into single- or multi-SNP events as above. For male NCO events, we analysed all such events. For female events, as noted in the main text, in human female meiosis, many complex NCO events occur, often extending >1 kb in size and occurring outside hotspots. Such events have been previously shown[7,8] to exhibit GC-bias, may not be positioned by PRDM9 binding or even reflect programmed meiotic DSBs, and are not directly comparable to the events in mice, since these complex events are not observed in our mice (see main text). We therefore (conservatively) excluded events of this type by analyzing only female NCO events overlapping DSB hotspots, and <1 kb in size. We used the distance between the first and last converted markers to define event size, and the "DSB" column within the Supplementary Data file to define whether events overlap DMC1 hotspots. The results (Supplementary Data 5) show that in both human males and females, single-SNP events show indistinguishable GC-bias to that in mice (69%, with $p<10^{-8}$ for the effect overall). Multiple-SNP NCO events show no evidence whatsoever of GC-bias ($p>0.9$ overall), and the difference is significant ($p=6x10^{-4}$). Therefore, humans appear overall to show – strikingly – near-identical properties to mice, at least at DSB hotspot sites.

To distinguish whether GC-bias is prevented by, or else prevents, multiple-SNP conversion tracts, we tested for a relationship between the strength of bias and the distance of a SNP to other SNPs in hybrid mice. SNPs nearby other heterozygous SNPs tend to be co-converted with those SNPs, meaning they will normally lie within multiple-SNP tracts, if converted. Therefore, if GC-bias is prevented by multiple-SNP conversion SNPs, such SNPs will show little or no bias. We therefore binned SNPs according to their distance to the nearest flanking SNP and plotted the strength of GC-bias for each bin (Fig. 4b). SNPs very near to other SNPs show no GC-bias, whilst those >100 bp from the nearest marker show approximately the 68% bias among all single-SNP conversion tracts. The extremely high observed rate of co-conversion of nearby marker pairs (95.1% of adjacent markers within 10 bp of a converted marker are also converted) implies that the number of SNPs in a conversion tract influences biased repair processing of DSBs towards or away from GC, rather than some process operating the other way around.

Thus, the strength of GC-bias depends on local SNP density, implying that the same SNP where one allele is A/T and the other is G/C will have different conversion rates and biases in different individuals, depending on alleles at surrounding SNPs. In humans, SNP densities are low (roughly 1 SNP per kb in Europeans) and so multiple-SNP conversion tracts (other than very long, typically complex tracts) are unusual; therefore, the similar GC-biases observed for single-SNP conversion tracts, of 68%, imply a common process might act in both species.

We reasoned that we could leverage the "non-biased" NCO events with longer tracts to understand whether the bias towards GC might depend on the invading (i.e. donor)

haplotype, or the recipient haplotype (in which the DSB occurred). There are 12 possible combinations of donor and recipient alleles: we estimated underlying (i.e. "non-biased") proportions of each from the multi-SNP NCO events (we averaged e.g. G to A and C to T conversions via obvious strand symmetry to yield 6 pooled types, in generating confidence intervals and Fig. 4c). In single-SNP observed NCO events, we then plot the fraction of each of the 6 possible types, divided (normalised) by these underlying proportions (Fig. 4c). We calculated 95% confidence intervals (CIs) for the odds of each of the 6 possibilities relative to the multi-SNP tracts (binomial test). As an alternative, we used all markers not involved in gene conversion events but immediately adjacent to converted markers to estimate the background probabilities of different SNP types within hotspots in the same way. This yielded essentially identical results (Supplementary Fig. 5b), with no significant difference in SNP composition as measured by underlying proportions between non-converted markers and markers in multi-SNP conversion tracts ($p=0.59$ by Chi-squared test with 5 d.f.).

We observed odds ratios >1 for all event types involving a recipient allele which is an A or T, and odds ratios <1 for all event types involving a recipient allele which is G or C. This suggests a bias driven by the potential recipient allele, i.e. the chromatid which the DSB occurs on. At DSB sites, it is possible in principle for nearby SNPs to be successfully converted from the homologous chromosome, or a potential conversion rejected, e.g. by resolution of heteroduplex DNA in favour of one background or the other. Given we do not observe mutations towards/away from GC bases altering the DSB rate, the observed GC-bias of NCO events is most simply explained by a tendency for the rejection of conversion of single SNPs, if the *recipient* haplotype has a G or a C at the same position, e.g. through the action of MMR, BER or NER proteins on

heteroduplex DNA. The relative odds of conversion occurring is approximately half of that if the recipient haplotype (that on which the DSB occurred) carries an A or T allele at the same position, so it is a strong effect. (We cannot determine whether the bias impacts only G or C bases, or both, because we do not observe strand for our NCO events). Importantly, this rejection does not seem to obviously depend on the type of the *donor* allele (other than it mismatching). For example, G to C conversions appear to occur at the lower rate while A to T conversions occur at the higher rate. As a caveat, there are relatively few such events, because these W to W transversion mutations are relatively rare, so the different rates observed for these events do not reach statistical significance ($p>0.05$). This means more complex models, e.g. with 3 classes of bias, remain possible.

Thus, our data imply a mechanism of GC-biased gene conversion arising through identification of mismatching bases in the donor haplotype relative to the recipient, sometimes leading to rejection of the donor allele if the recipient allele is a G/C base pair (Fig. 6). However, this "checking" process normally only occurs if there is just a single mismatching base within the potential conversion tract, so that potential multi-SNP conversion tracts show no bias.

Finally, we reasoned that rare observed complex recombination events (i.e. non-contiguous NCO and CO tracts) might be explained by every occasional ability of the same process to act within multi-SNP conversion tracts. We observed 12 SNPs not undergoing conversion but flanked by converted SNPs, i.e. within complex NCO events. We reasoned that the non-converted SNPs might be "rejected" by the above biased process, if in rare cases it is able to operate even in the context of a multi-SNP

potential conversion tract. If so, we would predict the *non-converted* markers should tend to show bias, with G/C bases on the recipient chromosome. Strikingly, we find all 12 SNPs show G/C bases on the potential recipient chromosome (and varying bases on the potential donor chromosome). The probability of observing this pattern by chance is approximately $1/2^{12}= 0.0002$. It is interesting that several complex events show >1 such SNP, so perhaps "escape" acts at the level of the entire event. Similarly, we observe 7 complex *de novo* CO events. All involve a single "missing" SNP (not uniquely identifiable in 2 cases), which may therefore be explained as a rejected conversion of one SNP. In 6/7 cases this missing SNP has a G/C base on the potential recipient chromosome. Taken together, this yields a p-value among all complex events of p= 0.00004 (Binomial test, 1-sided). A previous human study[9] found a similar bias of up to 100% in apparently converted sites within complex crossovers, which also involved single SNPs, so it appears this phenomenon may extend to humans. Therefore, complex recombination events can be reinterpreted as otherwise normal, continuous-tract events, but where a SNP is "rejected" for conversion, by a near 100% GC-biased process. Moreover, this process involves rejection of bases where the potential recipient chromosome carries a G or a C, exactly as in NCO GC-bias.

Therefore, the apparently distinct phenomena of GC-biased NCO events, and the occurrence of occasional complex NCO and CO events, might be explained by a common underlying model of biased repair, involving rejection of single "incoming" SNPs where the existing allele is a G or C base. Given it does not correlate with DSB initiation, this phenomenon most plausibly arises via biased heteroduplex repair machinery. Under this model, the bias appears to be close to 100%, but it does not

impact all NCO events, yielding a maximal observed NCO bias (identical in humans, and in mice) of around 68%.

We inferred the rate at which a strongly GC-biased repair process would have to occur to yield the observed GC-biased gene conversion rate at single-SNP sites (68% of converted sites being A/T to G/C). Given the results in complex NCO events, we assume this process has a GC bias close to 100%, which prevents conversion where the recipient chromosome is a G/C. However, that process only acts some of the time, say with probability p, while the normal strand-biased process occurs with probability 1-p. Then, given there's a single-SNP mismatch in a tract, and assuming the mismatch is GC->AT as often as AT->GC, each with probability q=0.5:

$$\text{P(conversion GC to AT)} = q(1-p) \text{ and} \quad (10)$$

$$\text{P(no conversion GC to AT)} = qp\text{, and} \quad (11)$$

$$\text{P(conversion AT to GC)} = q\text{,} \quad (12)$$

with the probability of no conversion from AT to GC being small, given estimates of the number of DSBs versus the number of CO/NCO events per meiosis.

Given our data,

$$0.68 = \text{P(conversion AT to GC | conversion observed)} = \frac{q}{q+q(1-p)} = \frac{1}{2-p}. \quad (13)$$

Solving this yields p=0.53. Thus, the data can be explained by simple model in which a distinct mismatch repair process acts to prevent gene conversion at 53% of sites where the recipient chromosome contains a G/C at a single mismatch site (or, in principle, at 100% of sites where the recipient chromosome contains a G at a single mismatch site; Fig. 6).

We reasoned that the default, non-GC biased repair pathway that operates at most sites (except 53% of single-SNP GC sites) would have to be biased towards the donor strand. Given our tract length estimates, power estimates, SNP density estimates, and number of observed NCO events, we calculated that the proposed number of unobservable GC restorations per meiosis is compatible with our estimate of ~300 DSBs per meiosis (Supplementary Fig. 8). An alternative model in which the default repair pathway chooses either strand from the heteroduplex with equal probability would require an unacceptably large number of DSBs (~465, Supplementary Fig. 8b). The proportion of NCOs overlapping 0, 1, or 2 SNPs (79%, 16.7%, 4.3%) was determined by simulation, drawing a tract length *n* from an exponential(1/36.2) distribution (36.2 bp being the mean tract length estimate when combining all events together), then computing a binomial probability of having more than *k* SNPs in *n* bases (with $p = 0.0072$). The proportion of SNPs representing W-W or S-S transversions (14%) was determined empirically by examining all SNPs within 200 bp of a DMC1 peak centre. The proportions repairing via each hypothetical pathway were fitted to yield the observed GC-bias in the data subject to the constraints of each pathway. The mean number of observable events (i.e. events overlapping >=1 converted SNP) in each meiosis was computed as (183 observed F2 NCOs / 0.74 power) * (4 gametes per meiosis / 22 F2 meioses total) = 45, so our model had to produce close to this many observable events (both yield 46). Another line of evidence supporting the donor-biased model comes from transmission of “cold” PRDM9 alleles at asymmetric hotspots, the cause of hotspot erosion. We filtered our F5 de novo CO events to those within highly (>95%) asymmetric hotspots, and further required these hotspots to contain a mutation within their PRDM9 motif. In 18 of these 19 CO events we observe transmission of the “cold”

binding site allele to offspring. Thus, heteroduplex repair at COs appears to be biased toward the donor homologue, and the same process may operate at most NCOs.

Supplementary Note 8: Rejection sampling algorithm for COs and NCOs, construction of Fig. 5 and Supplementary Fig. 7, and testing for impacts of asymmetry on event resolution

In testing for impacts of asymmetry on the number of NCO and CO events observed, relative to expectations from ChIP-seq data, we allowed for the following factors.

(i) Asymmetric hotspots have higher SNP density around binding motifs so have higher power to identify NCOs (CO events are not affected). We corrected this in our analyses of how symmetry impacts the number of observed NCO events in different hotspot types, by incorporating SNP density information to estimate power to detect events in each hotspot.

(ii) We tested for differences in the impacts of symmetry in COs vs, NCOs, males vs. females, *de-novo* versus inherited events, and for *Prdm9*^{Hum}-controlled versus *Prdm9*^{Cast}-controlled events. Because the CAST allele has co-evolved with the *castaneus* genome, some impacts of symmetry on recombination event resolution might be impacted (see below).

(iii) Strand can be identified for NCO events but not for CO events, allowing us to analyse events at asymmetric hotspots initiating on each strand separately. In particular, we can identify whether the homologue is strongly versus weakly marked by H3K4me3, for a given event.

In all analyses, we focussed on NCO and CO events occurring in the subset of hotspots containing an identified motif, and with well-defined estimated heat on each strand for both H3K4me3 and DMC1.

To correct for SNP density in our analyses of how symmetry impacts the number of observed NCO events in different hotspot types, we directly leveraged SNP density information to estimate (relative) power to detect events in each hotspot. For the hotspots that contain an identified motif, we give each SNP near the motif (<1 kb) a weight according to its location relative to the motif. The weight is defined using the distribution of NCOs around motifs (Fig. 3d), and so estimates the probability a NCO event initiating within the hotspot will incorporate this SNP (up to a constant of proportionality). Therefore, this quantifies the over-representation of this hotspot compared to the true number of DSBs resolving as NCO events occurring within it. Summing this weight over all SNPs then yields the relative power to detect bases falling within NCO events in each hotspot (so if there are no SNPs in a hotspot, the power to detect NCO events is zero, while hotspots with many SNPs near the motif itself have highest power). This yields a weight w_i for hotspot *i*. Multiplying the original hotspot heat (from DMC1 or H3K4me3) gives a power-corrected heat for the hotspot, used to define expectations for observable NCO events, and compare to actual observed NCOs.

Construction of Fig. 5a-b and Supplementary Fig. 7a-d:

For these figures, we compare CO and NCO events, for events of different types (e.g. male vs. female) and in different categories (e.g. $Prdm9^{Cast}$ versus $Prdm9^{Hum}$ controlled). To compare NCO and CO events, we used the weights w_i and rejection sampling. Within a hotspot and event category, we started with all observed events, and associated weight w_i for event *i*. Because NCO events are over-represented on average w_i-fold, relative to the ChIP-seq observations and to CO events, we retained NCO events with probability $\min\{\alpha/w_i,1\}$, and CO events/hotspots with probability min{1,

$w_i/\alpha\}$, where α is any constant. For any hotspot, the probability of retaining a NCO event is then immediately w_i/α times lower than that of a CO event, and so this perfectly reverses the over-representation of this hotspot in observed, versus initiated, NCO events (the constant α only impacts the overall number of NCO vs. CO events retained, not their spread, so does not impact the validity of this point). In practice, we used α=0.7 to retain similar numbers of CO and NCO events.

We then obtain comparable lists of hotspots with various H3K4me3 heats and symmetries, and DMC1 heats and symmetries, as well as observed NCO and CO events, in any given category. For Fig. 5a and Supplementary Fig. 7a-c, we next ordered hotspots by their H3K4me3 symmetry, defining 3 bins with equal expected number of events, according to DMC1-predicted overall heat of each hotspot. We order by H3K4me3 in order that our symmetry estimates are independent of the estimated heats; in practice, ordering by DMC1 symmetry made almost no difference to results (not shown). We compared the binned predictions to the actual number of events of different types observed – both NCO, and COs. We also obtained 95% CIs of the fraction of observed events in each category by bootstrapping events 1000 times. To obtain p-values for asymmetric hotspots, we obtained exact binomial p-values, to test the null hypothesis that the true proportion of events occurring in the asymmetric hotspots bin is 1/3.

For Fig. 5b and similar for Supplementary Fig. 7, we performed the same analysis, but now ordered hotspots by their DMC1 symmetry, defining 3 bins with equal expected number of events, according to H3K4me3-predicted overall heat of each hotspot. This tests whether H3K4me3-defined heats, which measure the extent of PRDM9 binding in

each bin, accurately predict where CO and NCO events occur. As before, though less strongly because DMC1 shows inflation in asymmetric hotspots (Supplementary Fig. 6g), we observe fewer events of all types in asymmetric hotspots, relative to expectations.

Construction of Supplementary Fig. 7e-h:

This group of panels is constructed as Supplementary Fig. 7a-d, but studies only CO events, so no rejection sampling was required. Rather than symmetry, we order hotspots based on their H3K4me3 or DMC1-estimated average homologous heat, and predict events within bins using their overall signal of DMC1 or H3K4me3. We separate hotspots depending on whether they are human-controlled or CAST-controlled. Homologous heat provides slightly stronger signals than symmetry itself, implying CO events avoid weak hotspots as well as asymmetric hotspots, i.e. all hotspots where the homologous chromosome is bound weakly.

Construction of Supplementary Fig. 7i-l:

This group of panels is constructed as for Supplementary Fig. 7a-d, but studies only NCO events. To account for power, we therefore resampled hotspots with weights proportional to w_i, and compare observed NCO events to expectations under this resampling. For NCO events, we can determine which homologue they occurred on. This allows us to test whether "homologous heat" (see "*Hotspot symmetry estimates*"), i.e. the strength of DMC1/H3K4me3 signal on the homologous chromosome, might more strongly determine whether NCO events occur than our overall single symmetry measure for a hotspot. We therefore now separated the two homologues for each hotspot, resulting in a predicted (DMC1 or H3K4me3) heat for each homologue, as

well as two complementary homologous heats. We ordered hotspots by this homologous heat (as in Fig. 5, we defined the fraction of events occurring on each homologue using the independent ChIP-seq data) and then used predicted heat to bin hotspots so that (as in Fig. 5) 1/3 of events are predicted to occur in each bin. We again separate hotspots depending on whether they are PRDM9^{Hum}-controlled or PRDM9^{Cast}-controlled, and otherwise proceed as in Fig. 5.

This revealed a strengthened signal relative to previous tests – few events are seen in hotspots with low homologous heat, i.e. where PRDM9 does not bind the homologous chromosome, implying NCO events strongly avoid occurring on both the hot allele of highly asymmetric hotspots, and at very weak hotspots. Conversely, NCO events occur preferentially on the cold allele of asymmetric hotspots, or strong hotspots more generally. Again, this occurs for both *Prdm9*^{Hum} and *Prdm9*^{Cast}.

Notably, although both differ significantly from expectations from both DMC1 and H3K4me3 data, we see somewhat stronger signals for *Prdm9*^{Hum} than *Prdm9*^{Cast}. Asymmetry in *Prdm9*^{Cast}-controlled hotspots is largely the result of evolutionary erosion, which can only occur when NCO or CO events themselves occur. Therefore, hotspots which evolve by chance to become asymmetric are preferentially sampled from those more active for these marks. This predicts that asymmetric hotspots for this allele may tend to have higher NCO and CO rates relative to PRDM9 binding strength as measured by H3K4me3, compared to these rates at random hotspots. In contrast, asymmetry at *Prdm9*^{Hum}-controlled hotspots is mainly due to SNPs occurring at random within the PRDM9 binding motifs inside these hotspots, so is immune to biases in NCO and CO rate, and this seems likely to explain our observation.

Supplementary References

1. Brick, K., Smagulova, F., Khil, P., Camerini-Otero, R. D. & Petukhova, G. V. Genetic recombination is directed away from functional genomic elements in mice. *Nature* 485, 642–645 (2012).
2. Jensen-Seaman, M. & Furey, T. Comparative recombination rates in the rat, mouse, and human genomes. *Genome Res.* 528–538 (2004).
3. Brunschwig, H. *et al.* Fine-scale maps of recombination rates and hotspots in the mouse genome. *Genetics* 191, 757–764 (2012).
4. Walker, M., Billings, T., Baker, C. L., Powers, N., Tian, H., Saxl, R. L., et al. Affinity-seq detects genome-wide PRDM9 binding sites and reveals the impact of prior chromatin modifications on mammalian recombination hotspot usage. *Epigenetics & Chromatin*, 8, 31 (2015).
5. Patel, L. et al. Dynamic reorganization of the genome shapes the recombination landscape in meiotic prophase. *Nat. Struct. Mol. Biol.* 26, 164–174 (2019).
6. Yan, J. et al. Histone H3 lysine 4 monomethylation modulates long-range chromatin interactions at enhancers. *Cell Res*. 28, 204–220 (2018).
7. Halldorsson, B. V *et al.* The rate of meiotic gene conversion varies by sex and age. *Nat. Genet.* 48, 1377–1384 (2016).
8. Williams, A. L. *et al.* Non-crossover gene conversions show strong GC bias and unexpected clustering in humans. *Elife* 4, 1–21 (2015).
9. Arbeithuber, B., Betancourt, A. J., Ebner, T. & Tiemann-Boege, I. Crossovers are associated with mutation and biased gene conversion at recombination hotspots. *Proc. Natl. Acad. Sci.* 112, 201416622 (2015).
