# Supplementary material for: A high-resolution map of non-crossover events reveals impacts of genetic diversity on mammalian meiotic recombination
Source: Nat Commun. 2019 Aug 29;10:3900. doi: 10.1038/s41467-019-11675-y (PMC6715734; doi:10.1038/s41467-019-11675-y)
Supplement: Supplementary file 3 — Description of Additional Supplementary Files [file 41467_2019_11675_MOESM3_ESM.pdf]

Description of Additional Supplementary Files

File Name: Supplementary Data 1
Description: Breeding and sequencing details for all mice (separate .csv file).
Column names are *breeding_ID, sex, generation, PRDM9_genotype, background, father_ID, mother_ID, birth_date, death_date, age_at_death, sequencing_ID, SRA_BioSample_Accession, WGS_PairedRead_Count, WGS_Coverage, Notes*. *Breeding_ID* matches the mother and father ID styles, while *sequencing_ID* matches those shown in Supplementary Figure 4 and listed in the SRA metadata. Individual accession numbers are included for each mouse (*SRA_BioSample_Accession*), as well as the number of Whole Genome Shotgun reads and rough fold coverage of the genome (*WGS_PairedRead_Count, WGS_Coverage)*. Mice used for ChIP-seq are indicated in the *Notes* column. Dates are in DD.MM.YY format.

File Name: Supplementary Data 2
Description: All DMC1 peaks on autosomes and ChrX, with force-called H3K4me3 values (separate .csv file).
Column 1: *Chromosome*
Column 2: *Start* (DSB peak centre - 500bp)
Column 3: *Stop* (DSB peak centre + 500bp)
Column 4: *DMC1_enrichment* (DMC1 ChIP-seq enrichment estimate)
Column 5: *DMC1_symmetry* (estimated fraction of DMC1 signal from the B6 chromosome)
Column 6: *H3K4me3_enrichment* (maximum likelihood estimator of force-called H3K4me3 ChIP-seq enrichment in the 1-kb surrounding the peak centre, normalised to local background coverage, minimum 0)
Column 7: *H3K4me3_symmetry* (expected fraction of H3K4me3 signal from the B6 chromosome)
Column 8: *H3K4me3_number_of_haplotype_informative_reads* (expected number of haplotype-informative H3K4me3 reads from signal; can be non-integers as it is an expected value; used to filter out unreliable symmetry estimates, typically by removing sites where this is less than 10)
Column 9: *H3K4me3_p_value* (p value from likelihood ratio test against null model with H3K4me3 enrichment = 0)
Column 10: *H3K4me3_filter_flag*: (1 = region overlaps an H3K4me3 peak likely to be PRDM9-independent)
Column 11: *Controlling_PRDM9_allele* (Prdm9 allele inferred to be controlling the DMC1 peak; either CAST, HUM, MULT, or KO)
Column 12: *H3K4me3_cov_r1* (no. of overlapping fragments from ChIP replicate 1)
Column 13: *H3K4me3_cov_r2* (no. of overlapping fragments from ChIP replicate 2)
Column 14: *H3K4me3_cov_input* (no. of overlapping fragments from ChIP Input; can also be used to filter out potential false positives)

File Name: Supplementary Data 3
Description: 2500 CO positions and properties (separate .csv file).
Column names are *Chromosome, Defining_SNP1, Defining_SNP2, Upstream_background, Downstream_background, Initiation, DMC1_peak, DMC1_enrichment, DMC1_symmetry, H3K4me3_enrichment, H3K4me3_symmetry, H3K4me3_number_of_haplotype_informative_reads, H3K4me3_pvalue, H3K4me3_filter_flag, Controlling_Prdm9_allele, CAST_motif_dis, CAST_motif_position, Human_motif_dis, Human_motif_position,Complex_Flag, Parental_origin, Sample_ID, Sample_ID_alt1, Sample_ID_alt2, Generation*, *Defining_SNP1* and *Defining_SNP2* indicate the 1-based positions of the left and right SNPs defining the CO breakpoint interval. Initiation indicates on which background the DSB occurred, when known. *DMC1_peak* lists the position of the

centre of the overlapping DMC1 peak, around which H3K4me3 enrichment was force-called in a 1-kb bin (H3K4me3 enrichment is fold over background-1). *DMC1_symmetry* and *H3K4me3_symmetry* list the proportion of reads from the B6 background (0 is all Cast, 1 is all B6). *H3K4me3_pvalue* indicates the p-value from a likelihood ratio test of whether the enrichment of H3K4me3 above background is greater than 0. *H3K4me3_number_of_haplotype_informative_reads* is used to filter out peaks where a reliable H3K4me3 symmetry call cannot be made due to low coverage (usually filtering sites where this is <10). *H3K4me3_filter_flag* is 1 if the peak is potentially PRDM9-independent, 0 otherwise. *Controlling_Prdm9_allele* indicates the *Prdm9* allele determined to be controlling the DMC1 peak. *CAST_motif** indicates the positions of the nearest $Prdm9^{Cast}$ and $Prdm9^{Hum}$ binding motifs, and their distance from the nearest edge of the CO interval (0 if inside the interval). *Complex_Flag* is 0 if the CO is simple, and 1 if it is complex (i.e. if there is a gene conversion within 1000 bp of the breakpoint from the same meiosis, which can only be certain for F2 and F5 de novo events). *Parental_origin* is listed when known (Maternal or Paternal). *Sample_ID* lists the mouse ID in the SRA, while *Sample_ID_alt1* lists the breeding ID and *Sample_ID_alt2* lists an alternative sample ID that may be present in sample BAM file headers.

File Name: Supplementary Data 4
Description: 1575 NCO positions and properties (separate .csv file).
Column names are: *Chromosome, First_converted_SNP, Last_converted_SNP, All_converted_SNPs, Number_of_converted_SNPs, Initiation, B6_allele, CAST_allele, DMC1_peak, DMC1_enrichment, DMC1_symmetry, H3K4me3_enrichment, H3K4me3_symmetry, H3K4me3_number_of_haplotype_informative_reads, H3K4me3_pvalue, H3K4me3_filter_flag, Controlling_Prdm9_allele, CAST_motif_distance, CAST_motif_position, Human_motif_distance, Human_motif_position, Nearest_Upstream_NonconvertedSNP Nearest_Downstream_NonconvertedSNP, Complex_NCO_Flag, Complex_CO_Flag, Parental_origin, Sample_ID, Sample_ID_alt1, Sample_ID_alt2, Generation*. Many column names are the same as for Supplementary Data 3. *First_converted_SNP* and *Last_converted_SNP* define the boundary positions of the minimal conversion tract. *All_converted_SNPs* lists the positions of all converted SNPs in the tract, separated by colons, equal to the *Number_of_converted_SNPs*. For each converted SNP (separated by colons): *B6_allele* indicates the allele on the B6 background, *CAST_allele* indicates the allele on the B6 background. Given *Initiation*, the donor and recipient alleles can de deduced. Motif distances are computed from the nearest converted SNP (0 if within the minimal tract). *Nearest_Upstream_Nonconverted_SNP and Nearest_Downstream_Nonconverted_SNP* indicate the positions of the nearest non-converted SNPs, which can be used to find the maximal possible tract length for each event. *Complex_NCO_Flag* is 0 if the NCO is simple, and 1 if it is complex (not a contiguous conversion tract). *Complex_CO_Flag* is 0 if the NCO does not occur within 1000 bp of a CO event in the same meiosis, and 1 if it does (these correspond to the *Complex_Flag* CO events from Supplementary Data 3).

File Name: Supplementary Data 5
Description: GC-bias for single-SNP versus multi-marker NCO events (separate .xlsx spreadsheet). As Supplementary Table 5, except NCO events are stratified according to whether they contain a single marker, or overlap multiple markers. Results show only human-controlled NCO events in F5 mice. In addition to Supplementary Table 5 categories, we stratify NCO events according to whether they occur in asymmetric vs. symmetric hotspots, strong or weak hotspots, or nearby versus distally from an identified PRDM9 binding motif. All categories show similar results, and GC-bias specific to single-SNP NCO events. We also reanalyse NCO events reported by Halldorsson *et al.* 2016 from human data and show an identical effect (last rows of the table).
