# Supplementary material for: A high-resolution map of non-crossover events reveals impacts of genetic diversity on mammalian meiotic recombination
Source: Nat Commun. 2019 Aug 29;10:3900. doi: 10.1038/s41467-019-11675-y (PMC6715734; doi:10.1038/s41467-019-11675-y)
Supplement: Supplementary file 9 — Reporting Summary [file 41467_2019_11675_MOESM9_ESM.pdf]

nature research

Corresponding author(s): Simon R. Myers

Reporting Summary

Nature Research wishes to improve the reproducibility of the work that we publish. This form provides structure for consistency and transparency in reporting. For further information on Nature Research policies, see Authors & Referees and the Editorial Policy Checklist.

Statistical parameters

When statistical analyses are reported, confirm that the following items are present in the relevant location (e.g. figure legend, table legend, main text, or Methods section).

n/a	Confirmed	
☐	☒	The exact sample size (n) for each experimental group/condition, given as a discrete number and unit of measurement
☐	☒	An indication of whether measurements were taken from distinct samples or whether the same sample was measured repeatedly
☐	☒	The statistical test(s) used AND whether they are one- or two-sided<br>*Only common tests should be described solely by name; describe more complex techniques in the Methods section.*
☐	☒	A description of all covariates tested
☐	☒	A description of any assumptions or corrections, such as tests of normality and adjustment for multiple comparisons
☐	☒	A full description of the statistics including central tendency (e.g. means) or other basic estimates (e.g. regression coefficient) AND variation (e.g. standard deviation) or associated estimates of uncertainty (e.g. confidence intervals)
☐	☒	For null hypothesis testing, the test statistic (e.g. F, t, r) with confidence intervals, effect sizes, degrees of freedom and P value noted<br>*Give P values as exact values whenever suitable.*
☐	☒	For Bayesian analysis, information on the choice of priors and Markov chain Monte Carlo settings
☒	☐	For hierarchical and complex designs, identification of the appropriate level for tests and full reporting of outcomes
☐	☒	Estimates of effect sizes (e.g. Cohen's d, Pearson's r), indicating how they were calculated
☐	☒	Clearly defined error bars<br>*State explicitly what error bars represent (e.g. SD, SE, CI)*

Our web collection on statistics for biologists may be useful.

Software and code

Policy information about availability of computer code

Data collection	Custom code was used to simulate events for power calculations (described in Methods; will be made available on Github prior to publication)
Data analysis	R (v 3.5.1), BWA (v 0.7.0), Stampy (v. 1.0.23), Picard (v 1.115), GATK (v 3.3-0), samtools (v 0.1.19), Bedtools (v2.23.0), peak calling and haplotype assignment code from Davies et al. 2016 (https://github.com/anjali-hinch/hybrid-rescue), peak calling code from Altemose et al. 2017 (https://github.com/elifesciences-publications/PRDM9-map), custom code available on Github (https://github.com/rosaranli/Detect_recombination_events_from_mouse_pedigree).

For manuscripts utilizing custom algorithms or software that are central to the research but not yet described in published literature, software must be made available to editors/reviewers upon request. We strongly encourage code deposition in a community repository (e.g. GitHub). See the Nature Research guidelines for submitting code & software for further information.

Data

Policy information about availability of data

All manuscripts must include a data availability statement. This statement should provide the following information, where applicable:

- Accession codes, unique identifiers, or web links for publicly available datasets
- A list of figures that have associated raw data
- A description of any restrictions on data availability

The mouse WGS sequencing data and variant calls are available under the SRA study accession SRP189007 [https://trace.ncbi.nlm.nih.gov/Traces/sra/sra.cgi?study=SRP189007]. The H3K4me3 ChIP-seq data are available under the GEO accession GSE119727 [https://www.ncbi.nlm.nih.gov/geo/query/acc.cgi?acc=GSE119727].

Field-specific reporting

Please select the best fit for your research. If you are not sure, read the appropriate sections before making your selection.

☒ Life sciences ☐ Behavioural & social sciences ☐ Ecological, evolutionary & environmental sciences

For a reference copy of the document with all sections, see nature.com/authors/policies/ReportingSummary-flat.pdf

Life sciences study design

All studies must disclose on these points even when the disclosure is negative.

Sample size	We aimed to detect thousands of noncrossover events to give sufficient power to describe their properties, taking into account that only events overlapping SNPs can be detected. We estimated that sequencing 18 F4/F5 families (2 parents + 4 offspring), would allow us to detect over 1000 events passing stringent filters. We also sequenced 2 F0 individuals and 11 F2 individuals to validate and extend our findings, for a total of 121 sequenced mice.
Data exclusions	data exclusions in the form of filtering are described in detail in the Methods and Supplementary info. Careful filtering of putative coverted sites was essential to distinguish them from sequencing errors; filtering of H3K4me3 peaks to exclude promoters was also essential. No other data exclusion applies.
Replication	Findings were compared between F2 and F5 mouse datasets for validation, where possible. A subset of gene conversion sites were validated by Sanger sequencing.
Randomization	samples were not allocated into experimental groups
Blinding	blinding was not relevant to the study, as samples were not allocated into experimental groups

Reporting for specific materials, systems and methods

Materials & experimental systems

n/a	Involved in the study
☐	☒ Unique biological materials
☐	☒ Antibodies
☐	☐ Eukaryotic cell lines
☐	☐ Palaeontology
☐	☒ Animals and other organisms
☐	☐ Human research participants

Methods

n/a	Involved in the study
☐	☒ ChIP-seq
☐	☐ Flow cytometry
☐	☐ MRI-based neuroimaging

Unique biological materials

Policy information about availability of materials

Obtaining unique materials	Where possible, we preserved spleen, liver, testis, and ear punch samples from each mouse in the final pedigree. These samples are available upon request.

Antibodies

Antibodies used	ChIP-grade rabbit polyclonal anti-H3K4me3 antibody (Abcam ab8580)
Validation	Antibody validated by manufacturer using ChIP for each new batch; validated by manufacturer to react with mouse antigens (https://www.abcam.com/histone-h3-tri-methyl-k4-antibody-chip-grade-ab8580.html); ChIP-seq data generated here show expected profiles at gene promoters and PRDM9 binding sites

Eukaryotic cell lines

Policy information about cell lines

Cell line source(s)	*State the source of each cell line used.*
Authentication	*Describe the authentication procedures for each cell line used OR declare that none of the cell lines used were authenticated.*
Mycoplasma contamination	*Confirm that all cell lines tested negative for mycoplasma contamination OR describe the results of the testing for mycoplasma contamination OR declare that the cell lines were not tested for mycoplasma contamination.*
Commonly misidentified lines (See ICLAC register)	*Name any commonly misidentified cell lines used in the study and provide a rationale for their use.*

Palaeontology

Specimen provenance	*Provide provenance information for specimens and describe permits that were obtained for the work (including the name of the issuing authority, the date of issue, and any identifying information).*
Specimen deposition	*Indicate where the specimens have been deposited to permit free access by other researchers.*
Dating methods	*If new dates are provided, describe how they were obtained (e.g. collection, storage, sample pretreatment and measurement), where they were obtained (i.e. lab name), the calibration program and the protocol for quality assurance OR state that no new dates are provided.*

☐ Tick this box to confirm that the raw and calibrated dates are available in the paper or in Supplementary Information.

Animals and other organisms

Policy information about studies involving animals; ARRIVE guidelines recommended for reporting animal research

Laboratory animals	Mus musculus castaneus (CAST/EIj strain), and Mus musculus domesticus (C57BL/6J strain) transgenic at the PRDM9 zinc finger domain (generated and described by Davies et al. 2016), and their cross (B6xCAST)F1 followed by 3 generations of interbreeding (F2-F4) to generate F5 mice. All mice were less than 1 year old at the time of culling, and an even balance of male and female samples were sequenced.
Wild animals	study did not involve wild animals
Field-collected samples	study did not involve wild animals

Human research participants

Policy information about studies involving human research participants

Population characteristics	*Describe the covariate-relevant population characteristics of the human research participants (e.g. age, gender, genotypic information, past and current diagnosis and treatment categories). If you filled out the behavioural & social sciences study design questions and have nothing to add here, write "See above."*
Recruitment	*Describe how participants were recruited. Outline any potential self-selection bias or other biases that may be present and how these are likely to impact results.*

ChIP-seq

Data deposition

☒ Confirm that both raw and final processed data have been deposited in a public database such as GEO.

☒ Confirm that you have deposited or provided access to graph files (e.g. BED files) for the called peaks.

Data access links *May remain private before publication.*	https://www.ncbi.nlm.nih.gov/geo/query/acc.cgi?acc=GSE119727

Files in database submission	Raw sequencing read files (3 samples multiplexed across two lanes); Intermediate bedgraph files showing filtered read pair positions; bed files showing de novo and force-called peak positions, enrichments, and p-values
Genome browser session (e.g. UCSC)	no longer applicable

Methodology

Replicates	Two ChIP replicates were generated, one testis each from two littermates, processed in parallel. One Input chromatin replicate was generated from one mouse.
Sequencing depth	ChIP and input chromatin DNA samples were sequenced in multiplexed paired-end 51-bp Illumina HiSeq2500 libraries (rapid run). Raw read pair numbers were 90466668, 72740395, and 85980827 for the Input, ChIP replicate 1, and ChIP replicate 2 samples, respectively. Filtered read pair numbers were 69956995, 63065759, and 70685675, respectively.
Antibodies	ChIP-grade rabbit polyclonal anti-H3K4me3 antibody (Abcam ab8580)
Peak calling parameters	Sequencing reads were aligned to mm10 using BWA aln2 (v. 0.7.0) followed by Stampy3 (v. 1.0.23, option bamkeepgoodreads), and reads not mapped in a proper pair with insert size smaller than 10 kb were removed. Read pairs representing likely PCR duplicates were also removed by samtools rmdup. Pairs for which neither read had a mapping quality score greater than 0 were removed. Fragment coverage was computed at each position in the genome and in 100-bp non-overlapping bins using in-house code and the samtools and bedtools packages. De novo peaks and force-called enrichment values were computed as described in Davies et al. 2016 and Altemose et al. 2017. These methods call peaks using information from both replicates plus the input control simultaneously.
Data quality	The percentage of ChIP-seq read pairs originating from signal (as opposed to background) was estimated to be 87.4% for both replicates by our peak-calling method.
Software	ChIP-seq peak calling and haplotype assignment code (from Davies et al. 2016 and Altemose et al. 2017) are available in Github (https://github.com/anjali-hinch/hybrid-rescue, https://github.com/elifesciences-publications/PRDM9-map).

Flow Cytometry

Plots

Confirm that:

☐ The axis labels state the marker and fluorochrome used (e.g. CD4-FITC).

☐ The axis scales are clearly visible. Include numbers along axes only for bottom left plot of group (a 'group' is an analysis of identical markers).

☐ All plots are contour plots with outliers or pseudocolor plots.

☐ A numerical value for number of cells or percentage (with statistics) is provided.

Methodology

Sample preparation	*Describe the sample preparation, detailing the biological source of the cells and any tissue processing steps used.*
Instrument	*Identify the instrument used for data collection, specifying make and model number.*
Software	*Describe the software used to collect and analyze the flow cytometry data. For custom code that has been deposited into a community repository, provide accession details.*
Cell population abundance	*Describe the abundance of the relevant cell populations within post-sort fractions, providing details on the purity of the samples and how it was determined.*
Gating strategy	*Describe the gating strategy used for all relevant experiments, specifying the preliminary FSC/SSC gates of the starting cell population, indicating where boundaries between "positive" and "negative" staining cell populations are defined.*

☐ Tick this box to confirm that a figure exemplifying the gating strategy is provided in the Supplementary Information.

Magnetic resonance imaging

Experimental design

Design type	*Indicate task or resting state; event-related or block design.*
Design specifications	*Specify the number of blocks, trials or experimental units per session and/or subject, and specify the length of each trial or block (if trials are blocked) and interval between trials.*
Behavioral performance measures	*State number and/or type of variables recorded (e.g. correct button press, response time) and what statistics were used to establish that the subjects were performing the task as expected (e.g. mean, range, and/or standard deviation across subjects).*

Acquisition

Imaging type(s) — *Specify: functional, structural, diffusion, perfusion.*

Field strength — *Specify in Tesla*

Sequence & imaging parameters — *Specify the pulse sequence type (gradient echo, spin echo, etc.), imaging type (EPI, spiral, etc.), field of view, matrix size, slice thickness, orientation and TE/TR/flip angle.*

Area of acquisition — *State whether a whole brain scan was used OR define the area of acquisition, describing how the region was determined.*

Diffusion MRI ☐ Used ☐ Not used

Preprocessing

Preprocessing software — *Provide detail on software version and revision number and on specific parameters (model/functions, brain extraction, segmentation, smoothing kernel size, etc.).*

Normalization — *If data were normalized/standardized, describe the approach(es): specify linear or non-linear and define image types used for transformation OR indicate that data were not normalized and explain rationale for lack of normalization.*

Normalization template — *Describe the template used for normalization/transformation, specifying subject space or group standardized space (e.g. original Talairach, MNI305, ICBM152) OR indicate that the data were not normalized.*

Noise and artifact removal — *Describe your procedure(s) for artifact and structured noise removal, specifying motion parameters, tissue signals and physiological signals (heart rate, respiration).*

Volume censoring — *Define your software and/or method and criteria for volume censoring, and state the extent of such censoring.*

Statistical modeling & inference

Model type and settings — *Specify type (mass univariate, multivariate, RSA, predictive, etc.) and describe essential details of the model at the first and second levels (e.g. fixed, random or mixed effects; drift or auto-correlation).*

Effect(s) tested — *Define precise effect in terms of the task or stimulus conditions instead of psychological concepts and indicate whether ANOVA or factorial designs were used.*

Specify type of analysis: ☐ Whole brain ☐ ROI-based ☐ Both

Statistic type for inference (See Eklund et al. 2016) — *Specify voxel-wise or cluster-wise and report all relevant parameters for cluster-wise methods.*

Correction — *Describe the type of correction and how it is obtained for multiple comparisons (e.g. FWE, FDR, permutation or Monte Carlo).*

Models & analysis

n/a	Involved in the study
☐	☐ Functional and/or effective connectivity
☐	☐ Graph analysis
☐	☐ Multivariate modeling or predictive analysis

Functional and/or effective connectivity — *Report the measures of dependence used and the model details (e.g. Pearson correlation, partial correlation, mutual information).*

Graph analysis — *Report the dependent variable and connectivity measure, specifying weighted graph or binarized graph, subject- or group-level, and the global and/or node summaries used (e.g. clustering coefficient, efficiency, etc.).*

Multivariate modeling and predictive analysis — *Specify independent variables, features extraction and dimension reduction, model, training and evaluation metrics.*
