# Supplementary material for: A high-resolution map of non-crossover events reveals impacts of genetic diversity on mammalian meiotic recombination
Source: Nat Commun. 2019 Aug 29;10:3900. doi: 10.1038/s41467-019-11675-y (PMC6715734; doi:10.1038/s41467-019-11675-y)
Supplement: Supplementary file 2 — Peer Review File [file 41467_2019_11675_MOESM2_ESM.pdf]

Reviewers' comments:

Reviewer #1 (Remarks to the Author):

Li et al. use F2 crosses and advanced intercrosses of mice generated between divergent strains to identify a large number of CO and NCO events. The high divergence between these lines allow the authors to characterize genomic features impacting CO and NCO events in unprecedented detail. This work results in a number of novel insights into recombination mechanisms including the impact of asymmetric hotpots on CO and NCO events, the impact of GC-biased gene conversion on single versus multi-SNP tracts, and PRDM9 allele-specific features of conversion tracts, among others. This paper is a tour de force and yields important insights into the mechanisms of recombination, and will be of great interest to the broad readership of Nature Communications. I have a few technical questions/comments that I outline below. My major concern is about the identification and filtering of NCO events and estimates of power to detect these.

Major comments:

Identifying NCO events and filtering - My primary technical concern with this paper is that it seemed that the way that SNPs were filtered prior to inference about NCO events was possibly ad-hoc and a large number of filters were applied. Were these filters determined by simulations or verified transmitted NCOs? There is insufficient detail about this in the text. Given this, I was concerned about the potential of false negatives from filtering to impact inferences about the nature of NCOs. The simulations the authors perform to evaluate their false negative rate are helpful but I was hoping the authors could provide more details about the specifics of how the simulations were done. When the authors note that they copy over the information from a donor mouse, are these reads re-mapped and re-called? To me this seems like a potentially large source of false negatives. I was also wondering if transmission of these events through the pedigrees between F4 and F5 animals could be a better way to measure false negative and false positive rates (i.e. inheritted NCOs in Table S2 versus candidate NCOs). It would be helpful to explore how filtering and false negatives could impact the inferred properties of NCOs.

Dominance of the Castaneus allele - The authors suggest that observed dominance of the castaneus PRDM9 allele could be due to the presence of stronger binding targets for this allele or expression differences. Regarding expression differences it would be interesting to use ASE in F1s to evaluate this but obviously this is outside of the scope of this paper. However regarding strong binding targets it seems like the authors could use the H3K4me3 and DMC1 data they have collected to evaluate this hypothesis.

Differences in conversion tract properties of the PRDM9-cast and PRDM9-human alleles - The observation that the human and castaneus PRDM9 alleles convert tracts of different lengths on average is exciting but I had a number of questions about this observation. First, is there evidence that the length of the motif recognized by the two PRDM9 alleles differs between the PRDM9-cast and PRDM9-human alleles? From the figures it looked like PRDM9-human might have a shorter motif. Are there systematic differences (i.e. due to the differences in base composition) that could explain this -- i.e. differences in surrounding SNP density that could, in combination with the impacts on NCO/CO rates reported elsewhere in the paper, impact the converted tract length? I was also wondering how power differences, particularly in the detection of NCO events, could influence the inferred length distributions for the two alleles. I.e. if the castaneus allele tends to target more SNP dense regions of the genome the delineation of these conversion tracts would be more precise.

Is there any concern that the HMM is less sensitive to complex NCO events and that this could influence the low number of complex events observed?

Minor comments:

The observations about co-converted SNPs and GC bias are very interesting and suggest some potential species-level differences that could be mentioned. For example, if this mechanism is observed broadly, as the authors allude to, species with high diversity should show less GC bias than related species with lower diversity. I

Impacts of asymmetric hotspots on NCO and CO frequency also has exciting implications for genome evolution in hybrids.

line 188 - where are the positioning impacts of CO and NCO events discussed?

line 234 - where are the human analyses described?

line 264 - is the lack of GC bias in non-complex CO conversion tracts also observed in human data?

line 368 - is this inconsistent with observations of relatively common complex conversions in yeast? i.e. https://www.ncbi.nlm.nih.gov/pmc/articles/PMC3649680/. Perhaps something different going on with PRDM9 dependent versus independent cases, would be interesting to look in a broader range of species.

line 472 - I found this sentence unclear.

line 564 - how were the NCO to be validated by Sanger sequencing chosen?

line 675 - How was tract length determined between the last converted SNP and the next non-converted SNP? I may have missed this.

line 690 - typo

The authors refer to transmitted and non-transmitted NCOs in the manuscript but I did not see where these analyses were described.

Figure 1D - what number of CO events are plotted here? Looking at this plot it seems much greater than the number expected given the number of individuals and generations.

Are there any possible impacts of reference bias on the analyses (especially in the incorporation of GATK quality scores) given a closer relationship between B6 and the mm10 reference compared to CAST?

Reviewer #2 (Remarks to the Author):

Comments to the Author
Review for manuscript entitled "A high-resolution map of non-crossover events reveals impacts of genetic diversity on mammalian meiotic recombination" by Ran Li and colleagues.

This is a unique and exciting work, in which Ran Li et al. combined a series of different measurement in a very large study of murine crosses to describe meiotic recombination. The power of this study is in the creation of a variety of high-resolution genome wide maps representing the different steps taking place during initiation and termination of meiosis. Overlapping this information such as PRDM9 binding, double strand break formation and repair into CO or NCO has allowed Li et al. to resolve in high detail differences in meiotic recombination between male and female maps and different PRDM9 alleles. Moreover, it provided important

insights in the preference of DSB formation, leading to conversions due to initiation bias, versus conversions formed due to heteroduplex repair that was shown to be GC-biased. Most importantly, this work provides further insights into the repair of asymmetric hotspots, which causes hybrid sterility. The combination of these high-resolution maps is highly unique and improves on our understanding of the complex processes taking place during meiosis.

Overall, I recommend the publication of this work with revisions. These include some rephrasing, more detailed analysis of the data, and finally some revisions in the interpretation. Specifics are described point-by point below:

Introduction
L56: and Fig.1a Could you add further information about details on how CO and NCO are identified from sequencing data of parents and offspring and how phasing is achieved (e.g. how a CO is distinguished from a long conversion tract of a NCO). Also add the number of sequenced individuals to figure 1.
L88-90: Complex events are more common in females, but still present in human males. Please add that complex events are not exclusive of females. .
L92: This is not completely true (see ref 18 and 28), both showing gBGC in NCOs.
L102: Ref 28 (Odenthal-Hesse et al.) give a good estimate about NCO and CO frequencies at six human recombination hotspots. At least this is not completely new in humans. Moreover, Ref 45 (Arbeithuber et al.) showed the location of COs in two human hotspots in relation to the DSB sites (ChIP-Seq data) and to the PRDM9 motifs. Also, Ref 13 and Ref 30 (Cole at al.) and Ref 39 (Boer et al.) made comprehensive studies in mice showing CO and NCO frequencies and their location. Maybe you want to reference these studies, with the caveat that unlike your study these are not genome wide analyses.

Results
L120: Copy-paste error: CAST is derived from Mus musculus castaneus.
L132-133: There are about 3x more DMC1 than H3K4me3 hotspots. Do you have an explanation for this? Are all of these H3K4me3 hotspots PRDM9 dependent (contain a PRDM9 motif)? Is there a preference of which H3K4me3 (or PRDM9) site is chosen for a DSB? What is the fraction of hotspots with both an H3K4me3 and DMC1 mark?
L134: Supplementary notes: it would be easier to make sections in the notes to clearly assign them to the main part (Supplementary Note 1, 2, 3,).
L138: Abbreviations (HMM) should be spelled out for the first time also in the main text.
L144: In Fig 1c, do the blue lines represent the CO centers (breakpoints)?
L145: Mention here also the number of sequenced F5 mice (it is stated somewhere else, I believe 72 F5 mice. Can you also add information of how many CO come from female or male F2 or F5s?
L157: Is the enrichment of recombination at telomeres similar in both sexes? Usually, male genetic maps are steeper at the telomeres (with a higher CO concentration). Can you plot in Fig1e male CO, male NCO, female CO and female NCO?
L176: referring also to Figure 2: Are there any regions with a DMC1 mark, but absent H3K4me3 which resolve in recombination? What is the fraction of H3K4me3 that does not overlap with CO/NCO?
Figure 2b) and c) What does real events mean, CO/NCO? Are all these only F2 events, or combined F2 and F5? I guess with “increasing heat” you mean sequence reads? Would combined events: DMC1 sites overlapping with H3K4me3 marks resulting in and COs and NCOs show the same trends?
Do you see differences in overlapping H3k4me3+DSB resulting in more CO vs NCO? In other words, is there a difference between CO vs NCO in panel b and c? How does this distribution look like for unknown controlled + KO hotspots? In other words, do you see PRDM9 independent CO/NCO events?
Can a site with a DMC1 mark but no H3K4me3 or vice versa (H3k4me3 but no DSB) result in CO or NCO?
How many of the hotspots have all characteristics of a hotspot (DMC1 mark, H3K4me3 mark, CO

or NCO and a PRDM9 binding motif)?
L177-178: How does the data look like for F5 mice or individually for males and females? Are these 4000 hottest hotspots more telomeric (in the case of males) and is there a difference in the use of these 4000 hotspots between males and females (sex bias). Can you briefly explain how you assess the heat of a hotspot?
L175-189: Fig. 2a is not referenced in the text.
L179: Figure 2d) and 2e) Which data is used here? We assume F2 since there is no PRDM9 cast in F5, but could you clarify this? Could you also provide this figure for F2 and F5 independently? Please specify also in the figure legend the sample size and the source of the data (F2, F5, males and females combined etc.).
Are hotspots presented in Fig 2 panel d and e and Supplementary Fig. 2a and 2b have all both DMC1 and H3K4me3 marks? If so, please specify in the figure legend.
L186: Are you referring with “recombination rates” to CO and NCOs or DSBs? In Fig 2f and 2g both are plotted and in the text only “recombination rates” is mentioned. Do you mean “by correlation (Fig 2f)” overlap of male versus female DSB intensities? Is DSB intensity the same as “DSB rate” used in the text? At a fine scale, do males have stronger or more DSBs?
L187: Could you please be more descriptive when stating “sex differences “here?
L192: Add in a short bracket the SNP density to help the reader (1 SNP for about every 170 bp stated somewhere else hidden in the text).
L196-200: It is hard to interpret the data of Fig 2h. Is it possible that the measured conversion tracts just reflect your SNP density (see Cole et al. 2010 which observed NCOs with a minimal conversion tract of 1 bp) instead of conversion tract length?
What proportion of NCO has 1 converted SNP vs > 1 SNP (co-conversions)? Can you conceive a measurement distinguishing simple conversions from co-conversions (these can also be stratified with 2, 3, 4, etc. converted SNPs). Are there differences between PRDM9cst vs PRDM9hum controlled hotspots or males vs females, strong vs weak DSB hotspots?
Humans lack the SNP resolution as you have in your mice. So, the NCO tract length is often overestimated. For example, if you detect a NCO event with a single converted SNP via pooled sperm typing, you calculate the tract length from the flanking unconverted SNPs of this NCO event. But if the next SNPs are 100 bp up- and downstream apart, the NCO tract length is 200 bp, although in reality it could be only 50 bp long.
Line 195: referring to Figure 2h.) L971: What do you mean by “conditional SNP being converted”? Do you mean SNP density?
L208: From Fig 2g, the ratio in your data seems to fluctuate around 70-80% (not 90%). Can you include a statement about this?. Is it possible that these NCO/CO ratios change between sexes or symmetric hotspots, or asymmetric hotspots in males vs asymmetric hotspots in females? Could you provide these analyses? These data could provide important insights into differences of DSB repair between males and females (see also comment to Fig 5).
L212-213: Are the NCO and CO plotted in Fig 3a-d and SM 3a-d the 183 (F2) + 1392 (F5) NCOs and 295 (F2) + 2205 (F5) CO detected? Somehow they seem less in the figures. In any case, can you add the sample size to the legend of the Figures 3a-d and SM 3a-d. Please also specify in the legend of Fig 3 that these are F2 events, correct? Would you be able to distinguish in the CO if they go from Cast to B6 or from B6 to Cast ◊ “reciprocal CO”?
Could you plot in a separate figure than Fig. 3a only co-conversions (more >1 SNP) and complex NCO? Do they behave differently than simple NCO? The same for complex CO?
Line 213: referring to Figure 3: Please specify what data was used in these plots (e.g. CO and NCO from F2 generation overlapping DMC1 and H3K4me3 marks). Can you plot also the COs or NCOs that did not have a PRDM9 motif, but instead use the DMC1 signal (approx. 15% of the data)? Do these behave different than the PRDM9 controlled NCO? The same for CO.
L226: spelling mistake: focused, and PRDM9 should be italicized
L231-237: You observed that co-conversions do not show gBGC, are these events different (e.g. PRDM9 independent recombination)? Also see comments to Figure 3.
DSB formation should be independent of GC bias, versus heteroduplex repair should be related to gBGC. See also ref 45.
L237: It was shown in Lesecque et al. 2013 (GC-biased gene conversion in yeast is specifically

associated with crossovers: molecular mechanisms and evolutionary significance) and Ref 45 (Arbeithuber et al.) that BER is likely the main cause for gBGC during the repair of heteroduplexes, and not DSB formation (also called initiation bias or meiotic drive) caused by differences in Prdm9 binding. Moreover, BER favors GC alleles or strong (S) alleles by excising thymines at DNA mismatches. As such, W>S transitions should be favored in gBGC.L239-241: Do more co-conversions occur within the PRDM9 motif versus outside? Multiple SNPs could lead to a more asymmetric hotspot and thus initiation bias.

L239-243: Referring to Figure 4b: Probably plot 4b as a function of the distance to the PRDM9 motif, not as a function of the distance to the nearest SNP. It could be that SNP density is a function of HS activity since SNPs are enriched within hotspots. Moreover, SNPs at the center of the HS can potentially disrupt PRDM9 binding and will be more likely be asymmetric or show an initiation bias

When you describe your observations, consider that initiation biases (one homologue is targeted for DSBs, but not the other due to differences in PRDM9 binding) and should NOT be GC biased ; however, in the flanking regions, conversion events are more likely the result of heteroduplex resolution, associated with MMR or BER and are likely GC biased.

It is possible that you are throwing together two different types of events in your NCO: one is SDSA (synthesis dependent strand annealing) resulting in NCOs initiated at the center of the hotspot (no branch migration). This type of event should not be biased to GC, unless a higher GC content results in a better binding of PRDM9 causing DSB asymmetry. The second type of conversions are more likely the result of heteroduplex repair flanking HS centers, and are associated with MMR or BER and are likely GC biased. Can you show this in an analysis in Fig 4, stratifying NCOs by “within binding motif” vs “outside binding motif”? This might also solve the mystery of why 1SNP NCO show gBGC, but not >1SNP NCOs. Also revise your model in Fig. 6 based on this information (see comments further on).

Next, you could distinguish which central NCOs are associated with hotspot asymmetry, also known as initiation bias (one homologue is targeted for DSBs but not the other, due to differences in PRDM9 binding). You can also assess if flanking NCO outside the binding motif are asymmetric or symmetric. The parameter of (a)symmetry is linked to DSB formation and should not be GC biased; whereas, events linked to heteroduplex repair are likely to be biased gBGC. Can you make these distinctions in your analysis of GC-bias?

L254: Do you mean Supplementary Figure 4b instead of 3b?

L254: Referring to Figure 4c: it would be more intuitive to categorize the changes into the following categories: S>W transitions, S>W transversions, W>S transitions and W>S transversion. This would allow the reader to better assess gBGC happening for W>S transitions and W>S transversion (W= weak AT and S = strong GC). Other changes do not lead to GC bias. Can you also please add the sample size of each category and the total number of events?

Why are S>S transversions underrepresented and W>W overrepresented in NCOs? Does this have to do with disruptive changes in the binding motif? Do you see differences if stratifying the data by PRDM9 allele?

Is there a reason for using “the relative proportion to the corresponding proportion of the nearest un-converted markers” instead of the relative proportion of all NCOs?

L265: The 500 bp in the Cole paper does not refer to GC bias, but to initiation bias between homologs. There are publications showing gBGC in CO like Lesecque et al. 2013, Ref 45 Arbeithuber 2015. In these datasets, GC-bias in CO was detected given on information of both reciprocals.

L277: Here is a citation error. Exchange no 43 (Tiemann-Boege et al) against 45 (Arbeithuber et al. 2015). In ref 43 there is no data about gBGC in complex events.

L275-283: Can you rephrase this paragraph? I am not sure what the message is.

Please rephrase the statement “GC-biased process which normally only operates within single-SNP conversion tracts”, since this is not completely established yet.

L291-310: You claim that asymmetric binding is conserved between F2 and F5 animals-can you specify what data you use get to this conclusion? Please add information to the figure legend on the animals you are analyzing in Supp Fig 5a, F2 and F5s?). Do you have data for the symmetry analysis of F5s? Can you also add sample sizes?

L312-315: We are confused of why you see asymmetric hotspots for PRDM9hum (Supp Fig5g). This seems to contradict your statement in L292-294 that the "PRDM9hum binds and initiates recombination equally well on both backgrounds". Can you verify that the asymmetric hotspots are dependent on PRDM9 binding? Specifically, that your proxy of H3K4me3 or DMC1 truly comes from PRDM9 activity, or that the asymmetry comes from differences in the hum motif (see also next comments).
L327: The next comments refer also to Figure 5 also for supplementary figure for F2 and F5. Could you please further subdivide CO and NCO into female and male CO or NCOs? There seems to be a lower number of DSB resolved as CO or NCO in male asymmetric hotspots (this is only the case for PRDMhum in F2 but not F5, and I wonder why). Such a plot would be informative as to differences between males and females in the ratio of CO/NCO events resolved in asymmetric hotspots. How do the unknown and Prdm9 KO (6% of the hotspots) behave in terms of DSB and H3k4me3 events resolved into CO or NCO?
Are the hotspots you are plotting (Fig 5) verified to be PRDM9-dependent (see Fig 2d and e)? If so, in those few captured CO and NCO events in asymmetric hotspots; how far is the NCO or CO from the PRDM9 binding motif?
Please define "intermediate".
L1007: Please elaborate how the DMC1-predicted fraction was estimated or refer to the appropriate SM Note or Methods and Materials.
L330: Please specify which Supp Note. Can you also specify the difference in the data between Fig 5a and Supp Fig 6a? For consistency purposes, can you make the same plot as Fig 5a for PRDM9Cst hotspots in Supp Fig 6?
L332: "reflect chance genetic variation" is a difficult expression. Could you re-phrase it?
L336-338: The binding of PRDM9 to a homolog is defined by the motif. If the motif is interrupted by an indel or a SNP, the binding affinity of PRDM9 changes. Can you remind the reader here again what proxies you are using to measure PRDM9 binding (H3k4me3 or SNPs in binding motif or both?) and which proxy for DSBs?
L338: You claim: "DSB occurring on the less bound chromosome of asymmetric hotspots" behave like symmetric hotspots in terms of NCO. How do CO behave in these cases? Do you have a plot or Figure showing this? It is very bizarre that the DSB happens at the chromosome not bound by PRDM9. How is this possible? Is something else introducing DSBs or is the high H3K4me3 not always a good proxy for PRDM9 binding.
L345: H3K4me3 might not always reflect the level of PRDM9 binding, especially in non-B DNA regions with low H3K4me3 that might have a good PRDM9 binding due to the open chromatin structure. Note that symmetric open chromatin structure in both homologues might help repair DSB via NCO or CO. This was hypothesized in the context of methylation in Ref 43.
L350: Inter-sister repair is quite plausible, but it still does not explain the lack of H3K4me3 signal upstream of the DSB formation.

Discussion
To make it easier on the reader, could you add a heading or one or two sentences to recapitulate your main findings starting each major points.
L366-371: Please move this to the result section, since it is not fitting as your first paragraph in the discussion. Can you add a few details on how many complex NCO or complex CO you observed? In which animals, what hotspots, etc.?
L366: They also occur in human males, but they are probably more frequent in females. See Ref 19 Halldorsson et al.
L367: grammar: nearly absent
L370: The ability to repair heteroduplexes decreases with female age and was recently reported in BioRxiv. The finding of more complex events could be related to this lack of heteroduplex repair, and not necessarily only by non-programmed DSBs.
L373: Is there a CpG bias in these complex NCO, where lesions are 5-meC dependent?
L376-378: The number of DSB resolved as a CO is also evolutionary constrained. Your data fits well the study of Segura et al. 2013. Proc. Biol. Sci. reporting a ratio of NCO/CO of 10:1 in mice versus 7:1 in primates. What controls this ratio is not fully understood, but factors like CO

interference, chromosome packaging, and fundamental number of chromosomes play an important role. These points should be mentioned in the discussion.
L393: use the word SNPs instead of mutations
L408: A depletion of CO+NCO events can also indicate a higher rate of inter-sister repair.
L413: Can you hypothesize how PRDM9 could assists with homologue search? Remember that PRDM9 is removed from the targeted homologue, once Spo11 cleaves the DNA exactly at the binding site of PRDM9.
L426: Can you reference a figure and/or other studies reporting this slower DSB repair in males? Could the higher overall methylation of DNA in males versus females during meiosis I play a role in this delay? See also reference 43 for a discussion on these sex differences in methylation during meiosis I.
L437-439: Which of your data shows unequivocally that gBGC operates downstream of DSB? Can you add 1-2 sentences summarizing these findings? Could you also use the original terminology gBGC and not gcBGC
L446-448: In your model, you are mixing DSB formation with DSB repair. You state that gBGC is the result of heteroduplex repair (L438) and occurs downstream of DSB formation (L438). Yet, in the first model explaining gBGC (Fig 6) you claim that this happens during DSB favoring one strand over the other. This does not make sense the way it is presented.
Also see Figure 6: Left panel: this seems DSB preference, known also as initiation bias or meiotic drive (see and cite Jeffreys work who described this first) explained now by the preferential PRDM9 binding.
L450: complex NCO or CO cannot be explained by an initiation bias; complex CO are likely the result of conversions during heteroduplex repair or template switching.
L454: gBGC was also shown in COs not only in complex CO in ref 45.
L459-467: This section is very confusing. Models of DSB repair using the unbroken homologue as a template are well established during strand invasion. Strand invasion is independent of MMR of heteroduplex repair acting downstream.
L478: BGC (misspelling)

Methods
L532-533: Add an additional sentence stating why it is important to remove potential hidden heterozygous sites in the F0 individuals?
L539: Please number your SM notes. So, it is much easier to find the correct one.
L541: refer to the specific SM about CO/NCO calling.
L544-546: What is the threshold? Add proportion of removed data.
L548: Is this the total number of identified NCOs in F2 (including co-conversions)?
L551-556: Can you add a sentence about the total number and proportion of inherited COs and NCOs versus de novo?
L559: what is the average tract length of these NCO events? Add also the length of your co-conversions? Do you see a difference in male vs female conversion tract lengths?
L560-561: How many CO and NCO events overlap with PRDM9 binding sites? (see also previous comments).
L572-576: Why is there a decreasing CO/NCO overlap with increasing generation time?
L579-599: Is there a different power for de novo vs. inherited tract length?
L651-667: Is this motif caller accessible online?
L710-712: How many SNPs or indels came from symmetric or asymmetric hotspot? Is there a difference in SNP or indel variant density? Can you distinguish in your hotspot initiation biases? Do asymmetric HS repair differentiate from symmetric cases?
L716: PWD acronym is not explained.
L736: In symmetric HS, do you observe a drift in homologous heat (e.g. DMC1)?

Supplementary Notes
L8: Please provide details how recombination events are classified into CO or NCO. For example, how do you differentiate a CO versus a NCO with a long conversion tract or a complex CO from a NCO?

L83: How many HS fall in this category?

Reviewer #3 (Remarks to the Author):

This is an impressive study that reports a large data set of meiotic recombination events in mouse, derived from sequencing recombinant populations. These data will be a valuable resource for the community. In addition the authors analyse the properties of these events and reveal some unexpected features of repair relating to mismatches. This is important, as the effects of heterozygosity/interhomolog polymorphism on meiotic recombination are relatively poorly understood. A further interesting dimension to these experiments is that the cross used contains two alleles of PRDM9 (the major protein driving mouse crossover locations), providing a means to test models of PDRM9 binding 'symmetry'.

The authors intercrossed two mouse subspecies (B6 with a human Prdm9 B allele sequence in the zinc finger array crossed with CAST) over 5 generations and sequenced 119 offspring (they sequenced 11 F2, 72 F5 and 36 F4 mice). These strains show a sequence divergence of ~0.7%. In total the authors identify ~1500 NCOs and ~2500 COs.
One interesting dimension is that polymorphism in PRDM9 binding sites causes differences in DSB hotspot activity. Previously shown that asymmetric binding associates with reduced fertility in hybrid mice. Asymmetric hotspots have greater DMC1 compared to H3K4me3 - consistent with longer repair. They also report that hotspots with high polymorphism and asymmetric binding, show stronger DMC1. Previous work in mammals has reported gene conversion events, which may be 1->1 kb and simple, or complex. Human NCO repair also show a ~68% GC bias - although as noted by the authors not all SNPs within a given hotspot show GC bias.

The authors compare CO and NCOs to H3K4me3 and DMC1 ChIP-seq and see a strong overlap, as expected. On page 8 line 177 the authors refer to '4,000 hottest hotspots' - it would be useful if the authors mentioned the total number of hotspots here, ie what proportion of the totalhotspots are these 4,000? Interestingly they see dominance of the Cast PRDM9 allele in terms of overlap with COs and NCOs. The authors explain this as being due to either differences in binding site strength, or a difference in expression level. The latter hypothesis should probably be tested using meiotic immunostaining. Interestingly, also detect a slight difference in NCO tract length associated with each allele.

The NCO events were associated with GC bias (60-64%). As the humanized PRDM9 allele has not co-evolved with the cis sequences this provides a particularly interesting opportunity to investigate GC bias in a naïve/non-evolved situation. Only GC bias was observed for single SNP GCs, and not for multiple SNP, and interestingly this also related to local SNP desnity, with high SNPs associating with greater mismatches and no GC bias. For single site NCOs, versus the longer events, which differ in GC bias, do these groups differ in other respects - for example, overlap with gene or transposon annotations, or chromatin state (nucleosome occupancy might be interesting to see)?
Previous work is relevant to effects of local heterozygosity on CO/NCO rates - for example at budding yeast URA3 hotspot greater mismtaches increased NCOs at the expense of COs (Borts and Haber 1986), with the dominant model being that this is mediated via MSH2 MutS MMR anticrossover effects. The situation in mice is more complicated due to the activity of PRDM9, but I feel like these previous studies in yeast should be discussed. I think it would be valuable to discuss the idea that heterozygosity may have an effect via formation of mismatches following interhomolog strand invasion also in the Introduction.

One general comment I have is that the data are analysed at fine-scale in detail, but I would value to see the recombination data plotted at larger scale along the chromosomes. For example, with a sliding window along the chromosomes how to NCO and CO frequency look? How do they relate to

(i) historical recombination (ie LD based) estimates, (ii) AT:GC/isochore structure, (iii) gene density, (iv) polymorphism density, (v) heterochromatin eg H3K9me3, and (vi) alpha satellite density. Some of these patterns are mentioned in the text (page 9 lines 185-189) but it would be interesting to see them plotted.

Minor points:

Line 85 - please explain why are gene conversions difficult to detect more clearly.
Lines 133-136. My reading of this is that you have just performed DSB analysis in males? How can you work out what is happening in female from these experiments?
Lines 141. Please explain ‘background changes’ more clearly.
Line 159 - perhaps modify to ‘telomeric enrichment’ to make this clear?
Line 166 - ChIP-seq peaks of what?

We thank all three reviewers for their comments and careful reading of our work. The manuscript has improved considerably as a result of their suggestions. In addition to addressing the reviewers' concerns as described below, we have done the following:

1) Finished uploading all sequencing data to the SRA (project accession PRJNA528086), and we are currently adding VCF files to accompany them
2) Added Supplementary Data 1 listing detailed information for all mice in the full breeding pedigree, and added Supplementary Figure 1 illustrating this full pedigree
3) Added Supplementary Data 2 containing detailed information on all DMC1 peaks and their force-called H3K4me3 values
4) Added Supplementary Data 3 and 4 listing detailed information on all 2500 COs and 1575 NCOs used in the study
5) Added Supplementary Table 2 examining the joint distribution of COs and NCOs across chromosomes in F2 mice
6) Added Supplementary Table 3 containing the results of a new GLM analysis of broad-scale effects on CO and NCO rates, and included the input data in a Source Data file
7) Added Supplementary Table 7 containing the results of the GLM analysis comparing the effects of local heterozygosity and hotspot asymmetry on CO and NCO rates
8) Added Supplementary Figure 8 containing flowcharts comparing different possible models of DSB repair decisions in meiosis
9) Added Figure 5c,d to better illustrate the point that the depletion of COs and NCOs at asymmetric hotspots only applies to events initiated on the more-bound homologue

All changes made are highlighted in yellow in the revised files.

Reviewers' comments:

Reviewer #1 (Remarks to the Author):

Li et al. use F2 crosses and advanced intercrosses of mice generated between divergent strains to identify a large number of CO and NCO events. The high divergence between these lines allow the authors to characterize genomic features impacting CO and NCO events in unprecedented detail. This work results in a number of novel insights into recombination mechanisms including the impact of asymmetric hotpots on CO and NCO events, the impact of GC-biased gene conversion on single versus multi-SNP tracts, and PRDM9 allele-specific features of conversion tracts, among others. This paper is a tour de force and yields important insights into the mechanisms of recombination, and will be of great interest to the broad readership of Nature Communications. I have a few technical questions/comments that I outline below. My major concern is about the identification and filtering of NCO events and estimates of power to detect these.

We thank the reviewer for their kind words.

Major comments:

Identifying NCO events and filtering - My primary technical concern with this paper is that it seemed that the way that SNPs were filtered prior to inference about NCO events was possibly ad-hoc and a large number of filters were applied. Were these filters determined by simulations or verified transmitted NCOs? There is insufficient detail about this in the text. Given this, I was concerned about the potential of false negatives from filtering to impact inferences about the nature of NCOs. The simulations the authors perform to evaluate their false negative rate are helpful but I was hoping the authors could provide more details about the specifics of how the simulations were done. When the authors note that they copy over the information from a donor mouse, are these reads re-mapped and re-called? To me this seems like a potentially large source of false negatives. I was also wondering if transmission of these events through the pedigrees between F4 and F5 animals could be a better way to measure false negative and false positive rates (i.e. inheritted NCOs in Table S2 versus candidate NCOs). It would be helpful to explore how filtering and false negatives could impact the inferred properties of NCOs.

The reviewer raises important concerns about the filters and false negative rate of detecting NCOs. The details of the filters are summarized in the Supplementary Table 1, and we have added more information about the filters in Supplementary Note 3. The filters are designed ad hoc in an unbiased way to remove: (1) false positive sites; (2) cryptic heterozygous sites from the B6 and CAST parental mice, which mimic NCOs; (3) SNPs with

low quality; and (4) potential deletions and duplications that mimic NCOs. We also try to recover some NCOs in which a subset of the converted SNPs are filtered by chance. For some filters (e.g. 14, 5, 10 and 11 thresholds) we used genome browser plots to visually identify suspect events, mainly in the F2 (rather than F5) mice. We think it is difficult to avoid such a process given that sequencing data properties are to an extent unique and we require a very low error rate. Therefore, we estimated the properties of our filters (false negative and false positive rate) after they were decided upon.

Specifically, all the filters were determined before we carried out any experimental validation of NCOs, so the resulting false positive rate estimates should be unbiased. Moreover, the filters were determined prior to evaluating our power to identify NCO events, which should therefore accurately reflect the false negative rate (because we simulate NCO events using the real data, but these do not match real events we are aiming to detect, but instead sample random regions to "convert").

We also have added more information to the section "Estimating power to identify NCOs" in the main text Methods section. To estimate what proportion of true NCOs are filtered by chance, we did the simulations using real data, in the same mice as we identified NCOs, which we believe is the best we can do to estimate power/false negatives. We shared the same concerns that re-mapping and re-calling could yield false negatives. Accordingly, our simulations use the variant call information from real mice, rather than reads themselves, so the information simulated is downstream of mapping and calling. For example, if we assume SNP S is converted from mouse A whose genetic background is B6/CAST to the mouse B whose genetic background is B6/B6 at this site, if there are 2 good reference reads and 3 alternative reads that cover SNP S in mouse A, then this site will be filtered. We will use information from mouse A for the filters 6-11, 14, 15, mouse B for the filters 5 and other sources for filters 1-4.

We considered the reviewer's suggestion to use the pedigree information from F4 and F5 mice to estimate false positive and false negative rates, but in order to estimate false positives and false negatives this way, we would have to reliably identify NCOs from both F4 and F5 mice. However, we only sequenced F4 mice to half the depth of F5 mice, so it remains far more difficult to confidently identify NCOs from F4 mice, and thus this would not yield a transferable measure of our power.

Dominance of the Castaneus allele - The authors suggest that observed dominance of the castaneus PRDM9 allele could be due to the presence of stronger binding targets for this allele or expression differences. Regarding expression differences it would be interesting to use ASE in F1s to evaluate this but obviously this is outside of the scope of this paper. However regarding strong binding targets it seems like the authors could use the H3K4me3 and DMC1 data they have collected to evaluate this hypothesis.

We have modified the relevant paragraph in the main text (lines 218-231) and added Supplementary Fig. 3e to provide further information, including another possible explanation for the observed dominance, we hope addressing the points raised:

> "Others have recently provided evidence that higher expression of *Prdm9*^{Cast} than *Prdm9*^{Hum} is also unlikely as an explanation[34], although we cannot rule out that the dominance of the *Prdm9*^{Cast} allele over *Prdm9*^{Hum} is due to the presence of greater levels of (or more stable) PRDM9^{Cast} protein.
>
> Alternatively, or in conjunction, stronger binding affinity of the CAST protein for its motif may underlie the observed dominance. Indeed, both a smaller number and weaker average intensity of PRDM9 (and mirroring H3K4me3) ChIP-seq peaks was recently reported in testes from B6 compared with CAST mice[41]. Similarly, we observe weaker H3K4me3 enrichment at *Prdm9*^{Hum}-controlled hotspots compared to *Prdm9*^{Cast}-controlled hotspots (Supplementary Fig. 3e). This suggests a stronger, longer-lasting association of the PRDM9^{Cast} protein with its binding sites compared to the PRDM9^{B6} protein. A related contributor to the dominance might be the propensity of *Prdm9*^{Hum} to bind to promoters, which appear resistant to either DSBs or recombination in humans[42], and which are removed from H3K4me3 analyses because promoters often contain PRDM9-independent H3K4me3. If PRDM9^{Hum} does bind promoters in mice, these sites would be nearly invisible to our analyses, making the *Prdm9*^{Cast} allele appear even more dominant."

Differences in conversion tract properties of the PRDM9-cast and PRDM9-human alleles - The observation that the human and castaneus PRDM9 alleles convert tracts of different lengths on average is exciting but I had a number of questions about this observation. First, is there evidence that the length of the motif recognized by

the two PRDM9 alleles differs between the PRDM9-cast and PRDM9-human alleles? From the figures it looked like PRDM9-human might have a shorter motif. Are there systematic differences (i.e. due to the differences in base composition) that could explain this -- i.e. differences in surrounding SNP density that could, in combination with the impacts on NCO/CO rates reported elsewhere in the paper, impact the converted tract length? I was also wondering how power differences, particularly in the detection of NCO events, could influence the inferred length distributions for the two alleles. I.e. if the castaneus allele tends to target more SNP dense regions of the genome the delineation of these conversion tracts would be more precise.

We thank the reviewer for these points. We present the observation of different tract lengths without having a conclusive explanation for its origin, though we have tried to rule out some hypotheses. We do not expect that a difference in PRDM9 alleles' binding footprints can explain the difference in tract length. The humanized allele has 12 zinc fingers, while the CAST allele has 11 (so they are expected to bind a 36-bp motif and a 33-bp motif, respectively, if all zinc fingers participate in binding). Prior work by our group has shown that the human B allele can bind several motifs with different lengths (Altemose et al. 2017), and their alignment implies differential use of one or two zinc fingers in the middle of the array. The twelfth zinc finger does not appear to confer much binding specificity, with the majority of hotspots containing motifs 32 bp or smaller. However, we caution that some zinc fingers may still be important for stabilizing binding even if they lack sequence specificity. Experimental results by others showed that the CAST and DOM2 PRDM9 alleles bind across their entire expected footprints, even at zinc finger positions with low sequence specificity; specifically, the CAST allele was shown to have a minimal binding footprint of 31 bp (Billings et al. 2013). We expect the minimal binding footprint for the human allele to be similar, given the likely differential use of internal zinc fingers. At worst, the 3-bp difference in expected binding footprint could not directly explain the 11-bp difference in mean conversion tract length between the two alleles, and the mechanism by which binding footprint would affect tract length is unclear.

We were also concerned that some kind of ascertainment bias could explain this observation, given that the CAST allele binds more AT-rich motifs and has caused meiotic drive in the CAST background, while the humanized allele binds GC-rich motifs and has not evolved with either genetic background. Indeed, we observe 58% more SNPs within 200 bp of CAST binding sites relative to HUM binding sites on average, likely owing to hotspot erosion, increased mutation (Arbeithuber 2015 and Halldorsson 2019), and gBGC on the CAST background (NB: there is a 164% increase in SNP density within binding motifs themselves, diminishing to a 4.9% increase 2kb away). Given the differences in SNP density surrounding the alleles' binding sites we did not simply take an average of minimal/maximal conversion tract lengths across all sites, which would likely yield smaller estimates for the allele with more nearby SNPs. Instead, we fit an exponential model based on the empirical observation of co-conversion of alleles *conditional on* their distance from each other (see Figure 2h). This conditioning should account for the difference in SNP density, with greater SNP density only improving the precision of estimates at the lower end of inter-SNP distance, but not biasing the overall trend of the data across different distances. The precision of this model fitting depends both on SNP density and the number of events used. In fact, because we were able to fit the model for the Humanized allele using both F2 and inherited/de novo F5 events, we actually had greater overall power to estimate tract length for the Humanized allele (using 815 total events overlapping hotspots), even at small length scales, compared to the Cast allele (for which we only have 409 F2 and inherited F5 events overlapping hotspots), but the Cast allele still showed a significantly shorter mean tract length. We have added these details to the relevant Methods subsection (lines 892-895):

> "Specifically, we used events that overlap a DMC1/H3K4me3 peak to avoid using any false positives. For *Prdm9*^{Hum}, we used de novo F5 NCOs along with F2 and inherited F5 NCOs controlled by *Prdm9*^{Hum} (815 events total). For *Prdm9*^{Cast}, we used F2 and inherited F5 NCOs controlled by *Prdm9*^{Cast} (409 events total)."

To further confirm that tract length estimation is robust to SNP density, we performed simulations by removing 5%-30% of SNPs (with step size 5%) within 1500 bp of all NCOs overlapping *Prdm9*^{Cast}-controlled hotspots (as these have greater overall SNP density for subsampling), and we repeated tract length estimation again. The resulting mean tract length estimates are very similar to previous estimates and do not show an obvious association with SNP density: 29.52, 30.55, 29.07, 28.42, 29.70, 31.40, respectively (vs 30 bp when using all SNPs). It is interesting to consider the reverse: whether greater local SNP density itself could limit the length of gene conversion tracts by some mismatch detecting mechanism, or somehow related to the greater conversion rate we predict in regions with SNPs in close proximity. To examine this, we separated converted SNPs from *Prdm9*^{Hum}-controlled hotspots (as these have not co-evolved with either genome) into two subsets: 349 SNPs in "low-density" hotspots with fewer than 3 SNPs in the central 200 bp surrounding the motif, and 494 SNPs in

“high-density” hotspots with 3 or more SNPs. Performing tract length estimation in each subset yielded mean estimates of 37 and 35 bp, which are not significantly different (bootstrap p-value 0.736; NB: slightly shorter estimates are obtained when using only events in motif-containing hotspots vs 40 bp when using all events). This indicates that neither hypothesis is likely to explain the large difference in tract length observed between *Prdm9*^{Hum} and *Prdm9*^{Cast}–controlled events. We have added this information in Supplementary Note 5.

Is there any concern that the HMM is less sensitive to complex NCO events and that this could influence the low number of complex events observed?

The HMM algorithm was run first and we smoothed the resulting initial background estimation by reverting inferred changes in background spanning <50 SNPs to the broader inferred background state. Such changes were tested as potential NCO events instead (Please refer to Figure 1b and Supplementary Note 2). Both simple and complex NCOs would be reverted to the background and flagged for testing, meaning that the HMM should have the same sensitivity for both simple NCOs and complex NCOs. We were concerned that our filters might bias against complex NCOs, so we aimed to recover complex NCOs that might have been filtered out. The last filter we use recovers potential converted sites <1000 bp from conversion events passing filters (to avoid removal of genuine long or complex events by accidentally failing filters), and we iterate until we do not recover any additional sites. This ensures that complex events will be recovered as long as there is at least one good converted site, so we should have the same sensitivity to detect complex NCOs as we do to detect simple NCOs.

Minor comments:

The observations about co-converted SNPs and GC bias are very interesting and suggest some potential species-level differences that could be mentioned. For example, if this mechanism is observed broadly, as the authors allude to, species with high diversity should show less GC bias than related species with lower diversity. I

Impacts of asymmetric hotspots on NCO and CO frequency also has exciting implications for genome evolution in hybrids.

line 188 - where are the positioning impacts of CO and NCO events discussed?

We have added a reference to Figure 1e (now updated to include centromere-proximal differences) at this point in the text and added additional details about broad-scale positioning to Supplementary Figure 2 and to the paragraph that follows (lines 170-181):

> “NCO and CO events, as well as DMC1 and H3K4me3, show enrichment nearer to telomeres, especially male COs (Fig. 1e and Supplementary Fig. 2c,d). This is broadly similar to patterns observed in other mice[36–38] and humans[15], although COs show somewhat stronger telomeric enrichment than NCOs. NCOs are also enriched near centromeres, especially on smaller chromosomes (Fig. 1e and Supplementary Fig. 2e). Interestingly, the telomere effect appears less pronounced among events controlled by the *Prdm9*^{Cast} allele (Supplementary Fig. 2f). By examining the joint distribution of COs and NCOs in F2 mice, we confirm that NCOs can occur on both sister chromatids within each pair, regardless of whether each sister also has a CO (Supplementary Table 2). We also found that broad-scale CO and NCO rates are positively associated with GC content, and after controlling for GC content, measures of localization with chromatin compartment A[39] *negatively* associate with CO outcomes (Supplementary Table 3 and Supplementary Note 4).”

line 234 - where are the human analyses described?

The human analyses are described in the last row in Supplementary Table 6, and we have added a slightly longer description in Supplementary Note 7.

line 264 - is the lack of GC bias in non-complex CO conversion tracts also observed in human data?

This does not seem completely clear. Two large genome-wide human studies (Halldorsson et al. 2016 and 2019) examined complex CO gene conversions but not simple CO gene conversions. This analysis is a little complex,

because gene conversion can only be observed indirectly at CO sites. Moreover if CO events and NCO events behave similarly, the long length of CO tracts (~300 bp) might mean given higher mouse SNP density, we do not observe GC-bias, while in humans (SNP density 1 per 1 kb vs. 1 per 170 bp in mice) a GC-bias might be expected. That is, human CO tracts may be likely to contain only one SNP and thus repair by a GC-biased mechanism (shown in Figure 6 for NCOs). There is one study of two human hotspots (Arbeithuber et al. 2015) which finds such a GC-bias might occur, although several other prior studies, of multiple human hotspots, from Alec Jeffreys's group did *not* find a consistent GC-bias in human hotspots for human CO events (but do find some bias due to the PRDM9 motif being disrupted). Thus, the picture for humans remains quite unclear, and it seems impossible to definitively answer this question at present.

line 368 - is this inconsistent with observations of relatively common complex conversions in yeast? i.e. https://www.ncbi.nlm.nih.gov/pmc/articles/PMC3649680/. Perhaps something different going on with PRDM9 dependent versus independent cases, would be interesting to look in a broader range of species.

Complex events do appear to be much more common in yeast, but it appears that somewhat different mechanisms operate in mammals. Humans have low rates of complex crossovers compared to yeast (see Halldorsson et al. 2019), and yeast NCOs are much longer than most mammalian NCOs, and lack GC-bias. We agree it would be interesting to look at complex CO and NCO rates in other mammalian species with different ages of sexual maturity, since complex NCOs and complex COs have both been shown to have a maternal age effect in humans (Halldorsson et al. 2016 and 2019, respectively). These complex events are also less likely to overlap recombination hotspots, suggesting they result from non-programmed DSBs. It would be interesting to examine complex events in mammals that lack PRDM9 but do have programmed DSBs and gBGC, like dogs (Berglund et al. 2014).

line 472 - I found this sentence unclear.

We have rewritten this entire subsection of the Discussion to improve clarity (lines 569-651).

line 564 - how were the NCO to be validated by Sanger sequencing chosen?

The NCO events that we validated were chosen from F2 de-novo events because F2 events were the first batch of events that we identified. We tested identified events both overlapping, and not overlapping, DMC1/H3K4me3 peaks. All 9 NCOs identified in F2 mice and located outside of DMC1/H3K4me3 peaks were tested. Of 79 NCOs initially identified in F2 mice and located within such a peak, a subset of 30 were randomly chosen for testing, excluding those contained within repetitive regions, and prioritizing those located within a single 500 bp amplification region to maximise the validation process. Of these, 19 primer sets gave a unique PCR product from DNA of all F0 and F2 mice tested, in quantities enabling Sanger sequencing, to form the validation set. Additional detail on the selection process is now given in the relevant Methods section (lines 743-749):

> "NCO validation by Sanger sequencing
> Of the 88 NCO events initially detected in F2 mice, we selected a subset for validation including 19/79 events located within a hotspot (prioritizing events located within a single 500 bp region and excluding those located within repetitive regions), as well as all 9 events located outside a hotspot. We PCR-amplified short regions (around 500 bp) overlapping the identified NCO sites using genomic DNA from the 2 F0 mice, the F2 mouse carrying the NCO, and up to 3 other related and/or unrelated F2 mice, using standard conditions (cycling conditions and primer sequences available upon request)."

line 675 - How was tract length determined between the last converted SNP and the next non-converted SNP? I may have missed this.

As the reviewer mentions, the tract must end somewhere between the last converted SNP and the next non-converted SNP. To estimate tract length we did not simply take an average of minimal/maximal conversion tract lengths across all sites. Instead, we fit an exponential model based on the empirical observation of co-conversion of alleles *conditional on* their distance from each other (see Figure 2h and reviewer responses above/below). This conditioning should account for the difference in SNP density at different sites, or equivalently, allow us to estimate underlying tract length properties without precisely knowing conversion tract beginning/end positions. We assume that the converted tract follows an exponential distribution (Figure 2h)

with rate parameter λ, where 1/λ is the mean tract length. While exponential tract lengths are not a fully accurate model, we can view this as a summary of tract properties, estimating the probability of co-conversion of pairs of markers as the distance between them increases. We computed a composite likelihood function for our NCOs and estimated λ via maximal likelihood. Specifically, for each converted site, viewing this site as a "focal" site, we examined the SNPs nearby and recorded for each SNP its distance from the focal SNP, and whether that SNP was also converted. If the SNP was also converted, then it was still in the gene conversion tract, otherwise it was not. Using this approach allowed our approach to be independent of SNP density, because we conditioned on SNP positions in our analysis. The probability that a SNP nearby a converted site is also converted is

$$\Pr(\text{SNP nearby converted}) = \Pr(\text{in}) = e^{-\lambda d},$$

where d is the distance from the nearby SNP to the converted site. The probability that a SNP nearby a converted site is not in the tract is 1-Pr(in). All the NCOs are independent so we can multiply these probabilities for each SNP in the windows to get the (composite) likelihood of the data:

$$\Pr(D) = \prod_{\text{all\_pairs}} \Pr(\text{in})^{x} \, (1 - \Pr(\text{in}))^{1-x}.$$

Here x=1 if the SNP nearby is also converted and x=0 otherwise. By maximising the likelihood using grid search for 1/λ from 1 to 1000 with step 0.1, we gained an estimate of tract length. Because pairs of SNPs are not in fact independent, this is not a true likelihood (though the resulting estimator is statistically consistent as the number of independent conversion events increases), and so to appropriately estimate uncertainty in the resulting estimates, we utilised bootstrapping of NCO events. Please refer to the Methods section "Estimation of NCO tract length for human-controlled and CAST-controlled events" (lines 861-895) for more information.

line 690 - typo

Thank you. We have corrected it in the text (line 876).

The authors refer to transmitted and non-transmitted NCOs in the manuscript but I did not see where these analyses were described.

We believe the reviewer is referring to the transmission of cold PRDM9 alleles in COs from asymmetric hotspots, described in the Discussion section. We have added this analysis to Supplementary Note 7.

Figure 1D - what number of CO events are plotted here? Looking at this plot it seems much greater than the number expected given the number of individuals and generations.

The number of CO events plotted here are 821 F5 de-novo COs plus 1384 distinct F5 inherited COs. We have made this clearer in the figure legend. The numbers are listed in the Supplementary Table 4. We combined *de-novo* and inherited COs for this analysis.

Are there any possible impacts of reference bias on the analyses (especially in the incorporation of GATK quality scores) given a closer relationship between B6 and the mm10 reference compared to CAST?

This is a good thing to check. We checked for reference bias, and did not find evidence of any obvious large effect. We would expect to have detected such a bias in F5 NCOs, which are controlled by the humanized allele, which by obvious symmetry binds equally well overall to the B6 and CAST backgrounds. If there were reference bias, we might expect to see systematically fewer events detected involving conversion from the CAST background compared to the B6 background. However, among the F5 de-novo NCOs that are controlled by *Prdm9*^{Hum}, in the 402 events that overlap a hotspot, we saw that 210 of them are initiated on the B6 chromosome and 192 of them are initiated on the CAST chromosome, which is non-significant, and not in the expected direction for reference bias (p= 0.3965). By simulation, we also see similar power to detect B6->CAST NCOs and CAST->B6 NCOs.

Reviewer #2 (Remarks to the Author):

Comments to the Author
Review for manuscript entitled "A high-resolution map of non-crossover events reveals impacts of genetic diversity on mammalian meiotic recombination" by Ran Li and colleagues.

This is a unique and exciting work, in which Ran Li et al. combined a series of different measurement in a very large study of murine crosses to describe meiotic recombination. The power of this study is in the creation of a variety of high-resolution genome wide maps representing the different steps taking place during initiation and termination of meiosis. Overlapping this information such as PRDM9 binding, double strand break formation and repair into CO or NCO has allowed Li et al. to resolve in high detail differences in meiotic recombination between male and female maps and different PRDM9 alleles. Moreover, it provided important insights in the preference of DSB formation, leading to conversions due to initiation bias, versus conversions formed due to heteroduplex repair that was shown to be GC-biased. Most importantly, this work provides further insights into the repair of asymmetric hotspots, which causes hybrid sterility. The combination of these high-resolution maps is highly unique and improves on our understanding of the complex processes taking place during meiosis.

Overall, I recommend the publication of this work with revisions. These include some rephrasing, more detailed analysis of the data, and finally some revisions in the interpretation. Specifics are described point-by point below:

We thank the reviewer for their assessment of our work and for their time in very carefully reviewing our manuscript.

Introduction
L56: and Fig.1a Could you add further information about details on how CO and NCO are identified from sequencing data of parents and offspring and how phasing is achieved (e.g. how a CO is distinguished from a long conversion tract of a NCO). Also add the number of sequenced individuals to figure 1.

We have modified the section Supplementary Note 2 to provide more clarity. To identify CO and NCO events, we do not have to phase the sequencing data. The HMM algorithm was run first and we smoothed the resulting initial background estimation by reverting inferred background changes spanning <50 SNPs to the broader inferred background state. These small background switches were tested as potential NCO events (Figure 1b and Supplementary Note 2). CO events are identified as background changes after this smoothing step. By this method, NCO tracts would be shorter than 50 SNPs and two consecutive COs would have to be at least 51 SNPs apart. Empirically, our longest detected NCO tracts span fewer than 10 SNPs, so the distinction is very clear.

We have added the number of sequenced individuals to the Figure 1 legend for clarity and corrected the number of families in Figure 1a.

L88-90: Complex events are more common in females, but still present in human males. Please add that complex events are not exclusive of females.

We thank the reviewer and have rephrased this statement (lines 92-93).

L92: This is not completely true (see ref 18 and 28), both showing gBGC in NCOs.

We are unsure which specific point the reviewer is referring to here, but we have added in a reference to Odenthal-Hesse et al. here (line 95). We mean to emphasise that the *molecular* mechanism of gBGC is still unresolved and have clarified the text accordingly.

L102: Ref 28 (Odenthal-Hesse et al.) give a good estimate about NCO and CO frequencies at six human recombination hotspots. At least this is not completely new in humans. Moreover, Ref 45 (Arbeithuber et al.) showed the location of COs in two human hotspots in relation to the DSB sites (ChIP-Seq data) and to the PRDM9 motifs. Also, Ref 13 and Ref 30 (Cole at al.) and Ref 39 (Boer et al.) made comprehensive studies in mice showing CO and NCO frequencies and their location. Maybe you want to reference these studies, with the caveat that unlike your study these are not genome wide analyses.

We thank the reviewer and have added these citations. We also emphasize that unlike these studies, our analysis is genome-wide and better powered to answer certain questions, such as estimating tract lengths, genome-wide NCO rates, and distributions of events around PRDM9 binding sites.

Results
L120: Copy-paste error: CAST is derived from Mus musculus castaneus.

We thank the reviewer and we have corrected it in the main text (line 125).

L132-133: There are about 3x more DMC1 than H3K4me3 hotspots. Do you have an explanation for this? Are all of these H3K4me3 hotspots PRDM9 dependent (contain a PRDM9 motif)? Is there a preference of which H3K4me3 (or PRDM9) site is chosen for a DSB? What is the fraction of hotspots with both an H3K4me3 and DMC1 mark?

These are very interesting questions to us. We think the reviewer means, "there are about 3x more H3K4me3 hotspots than DMC1 hotspots" (63050 H3K4me3 vs 23748 DMC1 autosomal peaks). This is after filtering out H3K4me3 regions that are likely to be PRDM9-independent (found in mice with other PRDM9 alleles or in other cell types—see Davies et al. 2016 for exact filtering method). We expect that the remaining H3K4me3 peaks are PRDM9 dependent, as they shift in mice with different PRDM9 alleles, although we have not tried (and would not expect) to find a strong PRDM9 motif match at every H3K4me3 peak, especially for the humanized allele, which appears to have some general affinity for GC-rich sequences (Davies et al. 2016 and Altemose et al. 2017). We expect there are a greater number of H3K4me3 peaks for several reasons. One is that some PRDM9 binding sites are less likely to get DSBs after binding and H3K4me3 deposition, likely owing to local epigenetic factors. We have not examined this phenomenon in detail here, as it has already been studied by several others (including e.g. Altemose et al. 2017, showing that nearby binding sites for ZNF proteins can suppress DSBs without affecting PRDM9 binding). Another explanation is that there are differences in experimental sensitivity between the two target proteins. PRDM9 likely marks several thousand H3K4me3 sites in each cell (Baker et al. 2014a), but only ~300 of them are chosen for DSB formation in each cell (Baudat 2007, Cole 2012, Paigen 2012), and DMC1 only remains present at each site while it is being repaired. Thus, PRDM9-dependent H3K4me3 proteins are much more abundant in testes than hotspot-associated DMC1 proteins, and are thus easier to pull down by immunoprecipitation. The specific protocols also differ in many other ways, including the antibody used and the use of a ssDNA enrichment step for DMC1. Furthermore, the patterns of DMC1 peaks and H3K4me3 peaks are quite different. They are necessarily called by different pipelines, which may lead to a different number of peaks, which can depend strongly on parameters like the p-value thresholds used. A more meaningful comparison is obtained by force-calling H3K4me3 enrichment in the 1-kb region surrounding DMC1 hotspot centres. By this method, we see that DMC1 and H3K4me3 enrichments are fairly strongly correlated (r = 0.67), and that the estimated fractions of H3K4me3 and DMC1 reads from each background (B6 or CAST) at each hotspot are very highly correlated (r = 0.88). Of the 22209/23748 autosomal DMC1 hotspots not overlapping potential PRDM9-independent H3K4me3 peaks (NB: this is a very stringent filter likely to exclude many truly PRDM9-dependent peaks and should *not* be taken to imply that >1500 DMC1 peaks occur at PRDM9-independent sites like promoters), 21748 of them (98%) show evidence of H3K4me3 enrichment at $p<0.05$ (96% at $p<0.001$). Furthermore, 99.4% of DMC1 peaks have an H3K4me3 enrichment estimate above 0, even if not significant. This supports the idea that the H3K4me3 peak set is a superset of the DMC1 peak set, for the reasons given above. We have added a line to the main text Results to clarify this (lines 139-140):

> "Essentially all DMC1 peaks show evidence of H3K4me3 enrichment (98% at $p<0.05$ by likelihood ratio testing; Supplementary Data 2)."

L134: Supplementary notes: it would be easier to make sections in the notes to clearly assign them to the main part (Supplementary Note 1, 2, 3,).

We thank the reviewer for the suggestion and have added numbers accordingly to the Supplementary Notes.

L138: Abbreviations (HMM) should be spelled out for the first time also in the main text.

We have corrected this in the main text (line 145).

L144: In Fig 1c, do the blue lines represent the CO centers (breakpoints)?

Essentially yes. The starting point of the blue lines on the left-hand side represent the position of the upstream SNP that defines the COs and each blue line has the same width to make it more visible (at this resolution, the line width is expected to be much larger than the breakpoint interval width). We have clarified the Figure 1 legend accordingly (line 188).

L145: Mention here also the number of sequenced F5 mice (it is stated somewhere else, I believe 72 F5 mice. Can you also add information of how many CO come from female or male F2 or F5s?

We have mentioned the number of F5 mice in the main text (line 155). For COs from F2s, from our data we are unable to assign parental origin as both parents have the same genotypes. For inherited COs from F5s, we are unable to assign parental origin either as we lack information about the genotypes when the COs were initiated. For the 821 de-novo COs from F5, we assigned 321 parental events, 382 maternal events and 118 unknown events. We have added this information to the main text (lines 163-164).

L157: Is the enrichment of recombination at telomeres similar in both sexes? Usually, male genetic maps are steeper at the telomeres (with a higher CO concentration). Can you plot in Fig1e male CO, male NCO, female CO and female NCO?

We have generated new supplementary figures with these plots (Supplementary Fig. 2d). We observe a strong increase in COs near telomeres among paternal crossovers, but not maternal crossovers (consistent with crossover data from Liu et al. 2014). We also find that paternal crossovers are more depleted near centromeres than are maternal crossovers (also consistent with crossover data from Liu et al. 2014). Given the small number of NCOs of known parental origin (251 total), we lack power to detect significant differences in broad-scale NCO patterns between the sexes. However, we do find that sex-averaged NCOs are also enriched at telomeres, similar to humans (Halldorsson et al. 2016), and we show that the NCO:CO rate appears to be elevated near centromeres, especially on smaller chromosomes (Supplementary Fig. 2e).

L176: referring also to Figure 2: Are there any regions with a DMC1 mark, but absent H3K4me3 which resolve in recombination? What is the fraction of H3K4me3 that does not overlap with CO/NCO?

As mentioned above, 98% of DMC1 peaks have significant H3K4me3 enrichment (at $p<0.05$), and 99.4% of DMC1 peaks have an H3K4me3 enrichment estimate above 0, even if not significant. We did not detect any CO or NCO events in the remaining 0.6% of peaks that show no evidence of H3K4me3 enrichment, so these are likely either false positives or very weak hotspots. The second question is impossible to address without having a number of detected CO/NCO events much greater than the number of H3K4me3 peaks. With our current set of ~4k NCO/CO events versus the ~60k H3K4me3 peaks, we simply cannot answer this question meaningfully. We can speculate based on previous work comparing H3K4me3 peaks to DMC1 peaks or SPO11 oligo peaks (Pratto et al. 2014, Lange et al. 2016, Davies et al. 2016, and others) that as we continue to sample a greater number of recombination events, the fraction of overlapped H3K4me3 peaks will likely saturate at a proportion less than 1, with the subset of H3K4me3 peaks that overlap DMC1 peaks likely to saturate first. We would expect the remaining DMC1 and H3K4me3 peaks that fail to overlap CO/NCO events to correspond to binding sites where either DSBs or downstream recombination events are suppressed by epigenetic factors (e.g. those described by Halldorsson et al. 2019).

Figure 2b) and c) What does real events mean, CO/NCO? Are all these only F2 events, or combined F2 and F5? I guess with "increasing heat" you mean sequence reads?

We have clarified these points in the figure legend (line 263). For Figure 2b) and c), real events mean combined CO and NCO events from F2 samples. The reason for this is that we can use all the ChIP-seq peaks for this comparison, since the DMC1 and H3K4me3 peaks reflect recombination happening in F1 meiotic cells, and the F2 NCO and CO events also arise from recombination events in F1 meiotic cells. For de-novo events in F5, as they are controlled by *Prdm9*^{Hum} only, we cannot use all the ChIP-seq peaks to do the analysis. By "heat" we mean ChIP-seq enrichment, which is computed by normalizing ChIP read coverage to input read coverage. We show here, for example, that the hottest sextile of DMC1 ChIP-seq peaks (~4k peaks) contain ~55% of the total DMC1 ChIP-seq signal within peaks, and correspondingly ~55% of recombination events (NCOs plus COs) within hotspots overlap this subset of peaks. We note that both panels B and C only look at COs/NCOs that overlap DMC1 peaks. That is, the H3K4me3 heat in panel c is computed by force-calling only at DMC1 peaks.

Would combined events: DMC1 sites overlapping with H3K4me3 marks resulting in and COs and NCOs show the same trends? Do you see differences in overlapping H3k4me3+DSB resulting in more CO vs NCO? In other words, is there a difference between CO vs NCO in panel b and c?

As mentioned above, essentially all DMC1 peaks have significant H3K4me3 enrichment. Furthermore, in panel c, the H3K4me3 heats are computed only at DMC1 peaks. We did not see a systematic difference between COs and NCOs in panel b and c so we decided to combine them to draw the main figure plots. We have provided the plots for COs and NCOs separately in Supplementary Fig. 3f.

How does this distribution look like for unknown controlled + KO hotspots? In other words, do you see PRDM9 independent CO/NCO events

In the F2 events, there is only 1 NCO that overlaps a KO hotspot and 3 NCOs that overlap "unknown" hotspots; 6 COs overlap KO and 15 COs overlap "unknown" hotspots. For "unknown" hotspots, each could be *Prdm9*^{Hum} or *Prdm9*^{Cast} controlled, and so these are not necessarily PRDM9-independent events; even the 7 events overlapping KO hotspots might occur within PRDM9-dependent hotspots at coincident sites, and so there are not enough of these events to generate the same type of plot. What is clear is that there are very few, or no, events independent of PRDM9.

Can a site with a DMC1 mark but no H3K4me3 or vice versa (H3K4me3 but no DSB) result in CO or NCO? How many of the hotspots have all characteristics of a hotspot (DMC1 mark, H3K4me3 mark, CO or NCO and a PRDM9 binding motif)?

As described above, since we rarely see a DMC1 peak with no H3K4me3 enrichment, we did not observe any COs or NCOs overlapping such peaks. Among the ~39,000 H3K4me3 peaks not overlapping a DMC1 peak, we see 275 CO or NCO events. In the entire set of 4075 COs and NCOs, 2517 overlap a DMC1 peak (with H3K4me3 enrichment) and occur near a PRDM9 binding motif. They overlap 1,898 unique DMC1 peaks. The most constraining requirement here, which reduces this number from 4075, is that of PRDM9 motif match overlap (especially for the humanized allele, which can bind GC-rich sequences with some affinity and so does not always bind a clearly recognizable single motif; in over 20% of cases). Supplementary Table 4 summarizes event overlaps with ChIP-seq peaks.

L177-178: How does the data look like for F5 mice or individually for males and females? Are these 4000 hottest hotspots more telomeric (in the case of males) and is there a difference in the use of these 4000 hotspots between males and females (sex bias). Can you briefly explain how you assess the heat of a hotspot?

Hotspot heat is approximated by DMC1 ChIP-seq enrichment—we have now clarified this in the text (lines 215-216). 39% of F5 de novo NCOs and 34% of F5 de novo COs occur in the hottest sixth of *Hum*-controlled hotspots (~4000 hottest peaks, determined by DMC1 enrichment from F1 testes heterozygous for *Prdm9*). This is likely lower than observed in F2s because of the lack of the dominant *Cast* allele in F5 mice, allowing events to distribute to a greater number of weaker *Hum*-controlled hotspots. We have added Supplementary Fig. 2d to show the distribution of DMC1 and H3K4me3 peaks averaged across the chromosomes, split into sextiles by their ChIP-seq enrichment values. Both H3K4me3 and DMC1 peaks increase in density near the telomere, and the hottest sixth of DMC1 peaks are especially telomere-enriched (until reaching the 5-Mb bin closest to the telomere, interestingly—it seems this telomere dip is also reflected in the CO distribution data seen in Figure 1e and is not attributable to sequencing or mapping bias). We do observe a greater proportion of male crossovers in the top 4000 peaks compared to female crossovers (92/321 male crossovers vs 75/382 female crossovers, Fisher's exact p=0.005788), consistent with sex bias. However, we do not observe a significant difference between male and female non-crossover placement in these 4000 peaks, probably owing to lack of power (33/121 male NCOs, 38/130 female NCOs, p= 0.7799).

L175-189: Fig. 2a is not referenced in the text.

Thanks, we have now referenced it (line 213).

L179: Figure 2d) and 2e) Which data is used here? We assume F2 since there is no PRDM9 cast in F5, but could you clarify this? Could you also provide this figure for F2 and F5 independently? Please specify also in the figure legend the sample size and the source of the data (F2, F5, males and females combined etc.).

For Figure 2d) and 2e) we used F2 events only as it allows us to compare *Prdm9*^{Cast} and *Prdm9*^{Hum} controlled events directly. We have clarified this in the legend. We cannot provide this figure for F5 mice because as the reviewer mentioned we do not have *Prdm9*^{Cast} in the F5 mice. Although there are some *Prdm9*^{Cast}-controlled events inherited from F2, they are not compatible directly with *Prdm9*^{Hum} controlled events. The number of events from F2 have been specified in the legend for 2d) and 2e). Because we cannot assign parental origin for F2 events as their F1 parents have exactly the same genotypes, we used total number of events only.

Are hotspots presented in Fig 2 panel d and e and Supplementary Fig. 2a and 2b have all both DMC1 and H3K4me3 marks? If so, please specify in the figure legend.

The hotspots we used here are DMC1 hotspots. For each DMC1 hotspot, we forced-called H3K4me3 intensity. Nearly all DMC1 hotspots have H3K4me3 enrichment. We have specified this in the figure legend (line 264).

L186: Are you referring with "recombination rates" to CO and NCOs or DSBs? In Fig 2f and 2g both are plotted and in the text only "recombination rates" is mentioned. Do you mean "by correlation (Fig 2f)" overlap of male versus female DSB intensities? Is DSB intensity the same as "DSB rate" used in the text? At a fine scale, do males have stronger or more DSBs?

Figure 2f shows underlying estimated correlations between the male and female DSB rate. This is defined as the total number of CO+NCO events leaving some observed trace in offspring. The legend has been edited to clarify this point (line 266). Figure 2g compares the NCO and CO rate, adding up (i.e. averaging) events across males and females. These plots use the method described in Methods to test correlations at different chosen scales. We edited this section of the main text (lines 234-241) to now read:

> "After accounting for sampling variation (Methods), we estimated correlation between recombination rates at different scales (Fig. 2f,g and Supplementary Fig. 3c,d). This revealed sex differences in recombination rates (combining COs and NCOs to gain power), with 100% correlation excluded, and decreasing correlations at broader scales (Fig. 2f). We observe strong (>70%) correlation between sex-averaged NCO and CO rates, although we also find very strong evidence that these events differ in their positioning along the chromosome, especially at broad scales (Fig. 1e and Supplementary Fig. 2), and the NCO rate is much higher than the CO rate at all scales."

Regarding the second point, total detected events in each sex are now tabulated in Supplementary Table 4; we find similar numbers of NCO event in each sex, and as expected, slightly more CO events in females.

L187: Could you please be more descriptive when stating "sex differences "here?

This is now explained more fully (lines 234-241); see response to previous point.

L192: Add in a short bracket the SNP density to help the reader (1 SNP for about every 170 bp stated somewhere else hidden in the text).

We have modified the main text accordingly (line 244):

> "We leveraged the high SNP density (~1 SNP per 170 bp) in our system…"

L196-200: It is hard to interpret the data of Fig 2h. Is it possible that the measured conversion tracts just reflect your SNP density (see Cole et al. 2010 which observed NCOs with a minimal conversion tract of 1 bp) instead of conversion tract length?

We account for SNP density alone by looking at co-conversion of marker pairs, so e.g. lower SNP density results in more uncertainty but not a bias. However it is possible because SNP density impacts e.g. GC-bias that this in turn alters the length of observed events. If so, the difference of observed tracts is still real (and we think interesting). Please see a more detailed response to this concern above and in Supplementary Note 5.

What proportion of NCO has 1 converted SNP vs > 1 SNP (co-conversions)? Can you conceive a measurement distinguishing simple conversions from co-conversions (these can also be stratified with 2, 3, 4, etc. converted SNPs). Are there differences between PRDM9cst vs PRDM9hum controlled hotspots or males vs females, strong vs weak DSB hotspots?

Among the 1575 NCOs detected, 1226 of them have a single converted SNP and 349 of them (22.2%) have more than one converted SNP. See Supplementary Data 4 for the subset that are Human-controlled NCOs in F5 mice, used for the comparative analysis of single- and multi-SNP NCOs.

Following the reviewer's suggestion, we computed the proportion of NCOs that are single-SNP conversions (as opposed to co-conversions) and compared this proportion between *Prdm9*^{Cast} vs *Prdm9*^{Hum} controlled hotspots, males vs females, and strong vs weak DSB hotspots. We did not detect a difference between *Prdm9*^{Cast} vs *Prdm9*^{Hum} controlled hotspots (p= 0.1092, two-sided Wilcoxon test), nor between males and females (p= 0.3864, two-sided Wilcoxon test). We also used various thresholds to define strong and weak DSB hotspots and did not detect a difference (see Supplementary Table 6 for an example).

Humans lack the SNP resolution as you have in your mice. So, the NCO tract length is often overestimated. For example, if you detect a NCO event with a single converted SNP via pooled sperm typing, you calculate the tract length from the flanking unconverted SNPs of this NCO event. But if the next SNPs are 100 bp up- and downstream apart, the NCO tract length is 200 bp, although in reality it could be only 50 bp long.

The reviewer raises an important point. We were also concerned with the way that SNP density affects our ability to estimate tract length in our mice, especially when comparing *Prdm9*^{Cast} vs *Prdm9*^{Hum} controlled hotspots (*Prdm9*^{Cast} hotspots have 58% more SNPs on average in the surrounding 200-bp window owing to historical mutations and meiotic drive effects). Because of this, we did not simply take an average of minimal/maximal conversion tract lengths across all sites, which would likely yield smaller estimates for events with more nearby SNPs. Instead, we fit an exponential model based on the empirical observation of co-conversion of alleles *conditional on* their distance from each other (see Figure 2h). This conditioning should account for the difference in SNP density, with greater SNP density only improving the *precision* of estimates at the lower end of inter-SNP distance, but not biasing the overall trend of the data across different distances. Previous studies have not modelled tract length in the same way, often just reporting the minimal and maximal tract lengths observed, without any statistical inference of the underlying distribution of tract lengths. The precision of this model fitting at different distances depends both on SNP density and the number of events used. If this exponential model fitting approach were applied to human data with the same number of detected events, the model fit would likely be poor, with enormous uncertainty at smaller inter-SNP distances, which are infrequently observed in humans. However, by greatly increasing the number of events detected one could begin to fit a similar model in human data (though this is still likely to yield very wide CI's even given the large set of deeply sequenced human pedigrees described by Halldorsson et al. 2019), aided by the fact that there is greater SNP density inside human recombination hotspots vs outside.

Please see responses above and Supplementary Note 5 for more information about how our tract length estimates are robust to SNP density.

Line 195: referring to Figure 2h.) L971: What do you mean by "conditional SNP being converted"? Do you mean SNP density?

We mean "given a SNP is converted, what is the probability that a nearby SNP is also converted?" For example, in Figure 2h, if you look at the results for an inter-SNP distance of 50 bp on the x axis, you can see that for *Prdm9*^{Cast}-controlled NCOs, the probability of co-conversion (y-axis) is about 0.2. That means if a given SNP is converted, then if a nearby SNP is 50 bp away, the probability that it's also in the NCO tract is 0.2. The larger the distance, the smaller the probability that a SNP is still in the NCO tract.

L208: From Fig 2g, the ratio in your data seems to fluctuate around 70-80% (not 90%). Can you include a statement about this?. Is it possible that these NCO/CO ratios change between sexes or symmetric hotspots, or asymmetric hotspots in males vs asymmetric hotspots in females? Could you provide these analyses? These data could provide important insights into differences of DSB repair between males and females (see also comment to Fig 5).

Fig 2g reports the *correlation* between CO and NCO rates, not the proportion of events that are NCOs vs COs, which hovers around 90% on average at all scales. Looking at COs and NCOs at broad chromosomal scales, we do observe an increase in the sex-averaged NCO:CO rate near centromeres, especially on smaller chromosomes (Fig. 1e and Supplementary Fig. 2e). although we lack power to detect whether this NCO enrichment is also subject to sex differences (Supplementary Fig. 2d). We do observe a much stronger depletion of COs near

centromeres in males compared to females (Supplementary Fig. 2d), so we might speculate that the centromere-proximal NCO:CO ratio is also likely to be greater in males compared to females.

The accurate estimation of the overall NCO:CO ratio depends on the number of NCOs, the number of COs, the power to detect NCOs, tract length, and SNP density around hotspots. If we assume the last three parameters are the same between males and females (NB: we show in another response that there is no evidence of tract length differences between males and females), then we can test whether the NCO:CO ratio is different between the sexes by comparing the number of detected NCOs and COs. Among F5 de-novo events, there are 321 paternal COs, 382 maternal COs, 121 paternal NCOs, and 130 maternal NCOs that have been assigned. Fisher's exact test shows these detected NCO:CO ratios are not significantly different ($p=0.5$).

If we further divide events into those occurring in symmetric and asymmetric hotspots, we can also test for differences between males and females in their observed NCO:CO ratios. For asymmetric hotspots (<0.1 or >0.9), these numbers are 51 paternal COs, 83 maternal COs, 24 paternal NCOs, 30 maternal NCOs, which shows no significant difference in NCO:CO between the sexes (Fisher's exact test $p=0.51$). For symmetric hotspots (>0.4 and <0.6), these numbers are 120, 131, 37, 43, which also show no significant difference ($p=0.90$).

To compare rates between symmetric and asymmetric hotspots, we have to perform rejection sampling to correct for the fact that asymmetric hotspots have greater SNP density and thus increased power to detect NCOs. Using *Prdm9*^{Hum}-controlled events and the threshold as in Figure 5b to separate asymmetric ($r*(1-r)<=0.112$) and symmetric hotspots ($r*(1-r)>=0.239$), the number of COs in asymmetric hotspots is 99, and the number of NCOs in asymmetric hotspots is 71, the number of COs in symmetric hotspots is 204, and the number of NCOs in symmetric hotspots is 194. There is no significant difference ($p=0.1419$, Fisher's exact test). This is consistent with our expectation, given our finding that the depletion of COs and NCOs is very similar in asymmetric hotspots.

L212-213: Are the NCO and CO plotted in Fig 3a-d and SM 3a-d the 183 (F2) + 1392 (F5) NCOs and 295 (F2) + 2205 (F5) CO detected? Somehow they seem less in the figures. In any case, can you add the sample size to the legend of the Figures 3a-d and SM 3a-d. Please also specify in the legend of Fig 3 that these are F2 events, correct? Would you be able to distinguish in the CO if they go from Cast to B6 or from B6 to Cast ◊ "reciprocal CO"?

NCOs and COs plotted in Fig. 3a-d are from F2 only and we have added this information, along with the number of events, to the figure legend (lines 298-301). We plotted NCO and COs in this plot as long as they overlap a DMC1 ChIP-seq peak containing a PRDM9 binding motif. For COs, for drawing purposes, both of the SNPs defining the COs have to be within 1000 bp around the motifs. In total these filters yielded 114 *Prdm9*^{Cast}-controlled NCOs, 17 *Prdm9*^{Hum}-controlled NCOs, 141 *Prdm9*^{Cast}-controlled COs, and 52 *Prdm9*^{Hum}-controlled COs plotted in Fig. 3. The same criteria were applied when we drew COs and NCOs from F5 mice in Supplementary Fig. 4.
For COs, if we assume that motifs are converted, and the motif overlaps a SNP, then sometimes we can tell with certainty if the initiating DSB occurs on the CAST or B6 background. However, if a motif is in the interval between the two SNPs defining a CO, then we are not able to determine on which background the DSB occurred (note: the COs in Fig. 3 do not specify the initiation background of each event). For example, if a CO goes from states CAST/CAST at SNP1 to B6/CAST at SNP2 and the motif falls on the CAST/CAST side (distal to SNP1), then the CO must have been initiated on the B6 background. In our data, we can only distinguish the origin of around 50% of COs by this method, even by making our assumption regarding motif conversion.

Could you plot in a separate figure than Fig. 3a only co-conversions (more >1 SNP) and complex NCO? Do they behave differently than simple NCO? The same for complex CO?

We have provided additional figures below to address the reviewer's questions. These show only co-conversions and complex NCOs surrounding the Cast (left) or Hum motif (right) from all generations. In terms of distance from the centre of the NCO tract to the motif, we did not detect any difference between the complex and simple NCOs (Wilcoxon two-sided test, $p=0.55$ for *Prdm9*^{Hum} controlled NCOs and $p=0.34$ for *Prdm9*^{Cast} controlled NCOs). For seven complex COs, while most overlap DMC1 hotspots, only 2 of them possess a clear PRDM9 binding motif so it is not possible to easily compare them with simple COs.

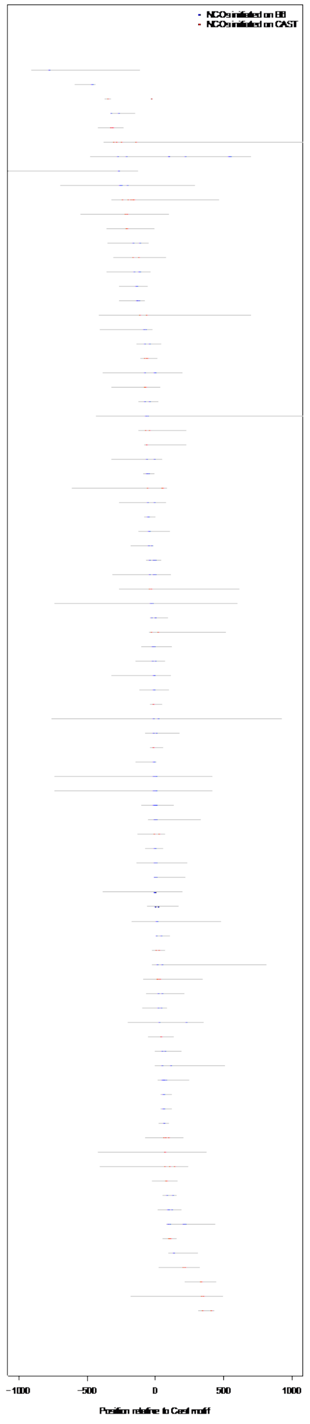

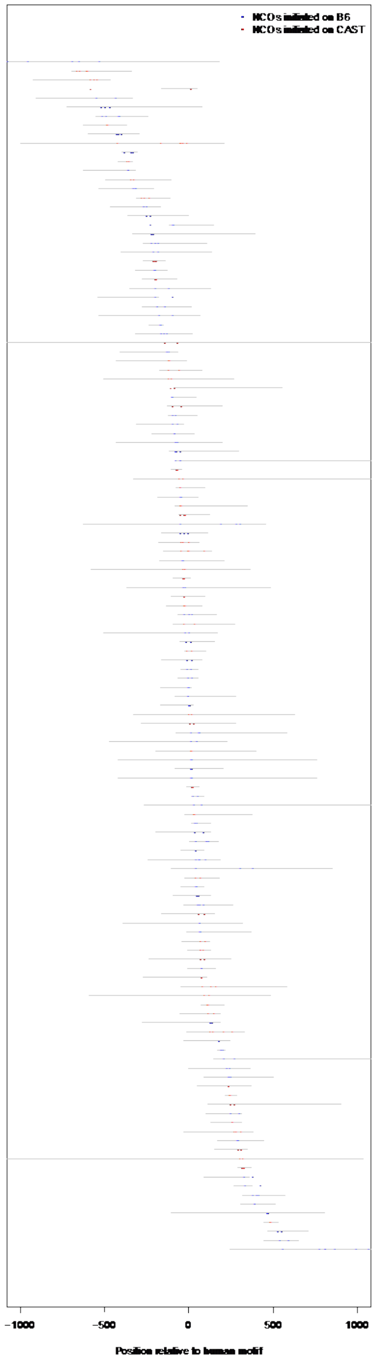

Line 213: referring to Figure 3: Please specify what data was used in these plots (e.g. CO and NCO from F2 generation overlapping DMC1 and H3K4me3 marks). Can you plot also the COs or NCOs that did not have a PRDM9 motif, but instead use the DMC1 signal (approx. 15% of the data)? Do these behave different than the PRDM9 controlled NCO? The same for CO.

Thanks, we have now specified this in the figure legend (lines 298-301). We plotted the COs and NCOs (all generations) that do not have a PRDM9 motif but do overlap a DMC1 hotspot and we found that these events also centre around the DMC1 peak centre (see plots below: COs on left, NCOs on right; DMC1 peak centres highlighted in yellow). The distances from the centres of COs and NCOs to the centres of the DMC1 peaks are not significantly different than the distances from PRDM9 motifs (two-sided Wilcoxon test, p=0.259 for NCOs and p=0.415 for COs).

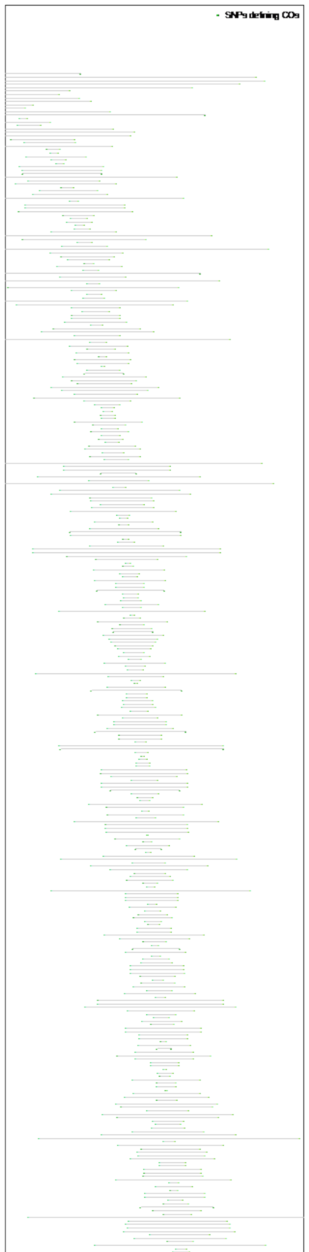
SNPs defining COs
COs do not overlap motif
−1000
−500
0
500
1000
Position relative to DMC1 center

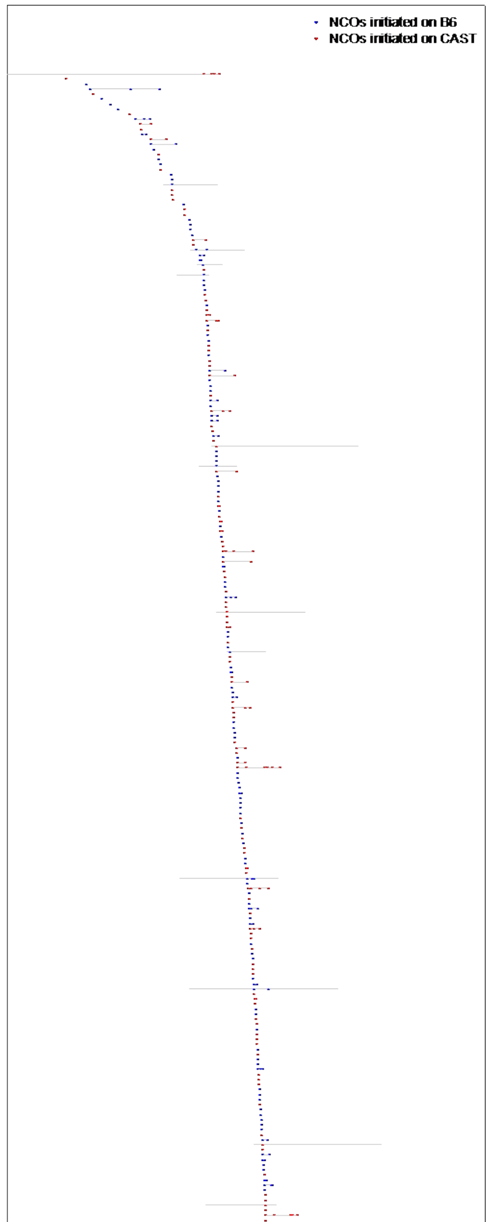
NCOs initiated on B6
NCOs initiated on CAST
−1000
−500
0
500
1000
Position relative to DMC1 center

L226: spelling mistake: focused, and PRDM9 should be italicized

Thank you. We have italicized PRDM9 (line 318). We note that "focussed" is the UK spelling of the word, in keeping with the rest of the document being in UK English.

L231-237: You observed that co-conversions do not show gBGC, are these events different (e.g. PRDM9 independent recombination)? Also see comments to Figure 3.
DSB formation should be independent of GC bias, versus heteroduplex repair should be related to gBGC. See also ref 45.

As mentioned above, we have not detected any systematic differences between single-SNP and multi-SNP conversion tracts in terms of PRDM9 allele, DMC1 enrichment, or sex differences. The difference in GC bias (reported in Supplementary Table 6) is evident even when conditioning on events being controlled by the Humanized allele, being de novo vs inherited, being maternal vs paternal, being symmetric vs asymmetric, having weak vs strong DMC1 enrichment, or being close or far from the PRDM9 motif.
We have added a citation to Arbeithuber et al. 2015 here (line 329).

L237: It was shown in Lesecque et al. 2013 (GC-biased gene conversion in yeast is specifically associated with crossovers: molecular mechanisms and evolutionary significance) and Ref 45 (Arbeithuber et al.) that BER is likely the main cause for gBGC during the repair of heteroduplexes, and not DSB formation (also called initiation bias or meiotic drive) caused by differences in Prdm9 binding. Moreover, BER favors GC alleles or strong (S) alleles by excising thymines at DNA mismatches. As such, W>S transitions should be favored in gBGC.

We note that Leseque et al. 2013 conclude the opposite regarding BER specifically: "We, therefore, conclude that in *S. cerevisiae*, gBGC occurs in conversion events associated with a long-patch repair machinery and that the contribution of BER to the gBGC process, if any, is at most very minor." We expect there, though, to be substantial differences in gBGC mechanisms between yeast and mammals given that gBGC is not observed at NCOs or complex COs in yeast. We have added a reference to Arbeithuber et al. 2015 here. We do not present the initiation-independence of gBGC as a novel finding, but simply confirm that the *difference* in gBGC that we observe between single- and multi-SNP tracts, which is novel, cannot be explained by another phenomenon like initiation bias.

L239-241: Do more co-conversions occur within the PRDM9 motif versus outside? Multiple SNPs could lead to a more asymmetric hotspot and thus initiation bias.

It is rare to detect NCOs overlapping a PRDM9 motif containing more than one SNP. That is, most co-conversion events contributing to the GC-bias difference that we observe occur outside of the PRDM9 motif. We also showed that when we condition on initiation bias (hotspot asymmetry), the GC-bias difference still holds (Supplementary Table 6 and Supplementary Fig. 5a). To more directly answer this question, we compared co-conversions occurring within the PRDM9 motif versus outside and did not detect a difference from simple NCOs for $Prdm9^{Cast}$-controlled events (31 out of 87 co-conversions occur within the PRDM9 motif versus 80 out of 314 simple NCOs occur within the PRDM9 motif; Fisher's exact test p=0.2024) or for $Prdm9^{Hum}$-controlled events (31 out of 132 co-conversions occur within PRDM9 motif versus 63 out of 386 simple NCOs; Fisher's exact test p=0.1304).

L239-243: Referring to Figure 4b: Probably plot 4b as a function of the distance to the PRDM9 motif, not as a function of the distance to the nearest SNP. It could be that SNP density is a function of HS activity since SNPs are enriched within hotspots. Moreover, SNPs at the center of the HS can potentially disrupt PRDM9 binding and will be more likely be asymmetric or show an initiation bias.

In Supplementary Table 6, we show the results highlighted in this figure hold for both $Prdm9^{Cast}$ and $Prdm9^{Hum}$ hotspots. For the latter, SNP density cannot be evolutionarily influenced by hotspot activity, so this cannot explain the results shown. It is critical for Figure 4 to plot distance to the nearest SNP because this alone (and completely) explains the signal, rather than positioning of events relative to the PRDM9 motif. There is no evidence whatsoever of GC-bias for SNPs in multi-SNP conversion tracts (in contrast to single SNP tracts), while the impacts suggested by the reviewer might weaken, but would not be expected to eliminate, such a signal. Indeed, in Supplementary Table 6 we consider whether NCO events positioned nearby, versus relatively distantly, from PRDM9 binding motifs behave differently, finding they do not (see next response point). Again, there is no evidence whatsoever of GC-bias for SNPs in multi-SNP conversion tracts (in contrast to single SNP

tracts), regardless of whether these overlap the PRDM9 motif or not, eliminating this as a possible explanation. By focussing on distance to the nearest SNP, we are testing whether our observations could be explained by e.g. tracts prematurely terminating near certain SNPs, so that GC-bias impacts whether NCOs contain multiple SNPs, inducing a relationship. However we see clearly that SNPs far from other SNPs show the strongest bias and SNPs very near other SNPs show no bias, meaning GC-bias is a varying property of particular SNPs. That is, GC-bias cannot be causing the difference in the number SNPs overlapped; rather it must be the other way around: the number of SNPs in NCO tracts influences the GC-biased repair/resolution process.

When you describe your observations, consider that initiation biases (one homologue is targeted for DSBs, but not the other due to differences in PRDM9 binding) and should NOT be GC biased ; however, in the flanking regions, conversion events are more likely the result of heteroduplex resolution, associated with MMR or BER and are likely GC biased.
It is possible that you are throwing together two different types of events in your NCO: one is SDSA (synthesis dependent strand annealing) resulting in NCOs initiated at the center of the hotspot (no branch migration). This type of event should not be biased to GC, unless a higher GC content results in a better binding of PRDM9 causing DSB asymmetry. The second type of conversions are more likely the result of heteroduplex repair flanking HS centers, and are associated with MMR or BER and are likely GC biased. Can you show this in an analysis in Fig 4, stratifying NCOs by “within binding motif” vs “outside binding motif”? This might also solve the mystery of why 1SNP NCO show gBGC, but not >1SNP NCOs. Also revise your model in Fig. 6 based on this information (see comments further on).
Next, you could distinguish which central NCOs are associated with hotspot asymmetry, also known as initiation bias (one homologue is targeted for DSBs but not the other, due to differences in PRDM9 binding). You can also assess if flanking NCO outside the binding motif are asymmetric or symmetric. The parameter of (a)symmetry is linked to DSB formation and should not be GC biased; whereas, events linked to heteroduplex repair are likely to be biased gBGC. Can you make these distinctions in your analysis of GC-bias?

We thank the reviewer for these comments. As shown in Supplementary Table 6, we did condition on NCO positioning and initiation bias (rows 13-14 and 7-8, respectively, with the latter also displayed in Supplementary Figure 5a), and we still observe GC-bias at single-SNP conversion tracts. For example (from row 13), we looked only at F5, Human-controlled events overlapping DMC1 peaks containing PRDM9 motifs, and we further took only the quartile of events closest to the PRDM9 motif, and we showed that these events are also GC-biased when overlapping single markers (GC-bias = 0.638 [0.513-0.750]) but not when overlapping multiple markers (GC bias = 0.436 [0.303-0.577]). This does not strictly look at mutations only within the PRDM9 binding motif, as suggested by the reviewer, but there would be too few such events to have statistical power to observe GC-bias. Lange et al. 2016 showed that most DSB breakpoints occur within about ~100 bp of the PRDM9 motif, so we would still expect to see some spread of any NCO repair mechanism around the PRDM9 binding motif. Our data do not support the possibility that a GC-neutral NCO mechanism operates on tracts close to the motif.

We also note that we observe a much tighter distribution of NCOs around PRDM9 binding motifs compared to COs, H4K4me3, and DMC1 (Fig. 3c,d), which is more consistent with predominantly SDSA-based repair (as suggested by Cole et al. 2014 and Lange et al. 2016 in mice). Indeed, we show that NCOs potentially overlap the PRDM9 binding site 70% of the time. This narrow distribution around the hotspot centre suggests that DHJ dissolution by branch migration is unlikely to be a dominant mechanism of forming NCOs. Martini et al. 2011 do show in yeast that a substantial minority of NCOs can be explained by DHJ dissolution, and they note this pattern is indistinguishable from SDSA occurring on both resected strands, though they suggest this may be less likely. These results in yeast should be interpreted in light of the fact that yeast NCOs are very different from mammalian NCOs: they tend to be much longer (~1 kb) and lack GC bias (Leseque et al. 2013). Crown et al. 2014 suggest that mechanisms involving both resected strands engaging with the homologue (like double-SDSA) are more likely than DHJ dissolution to explain patterns observed in Drosophila (although again Drosophila have very different NCOs than mammals). DHJ dissolution, or some other non-SDSA mechanism, could possibly play a larger role at some specific mammalian hotspots like the A3 hotspot studied by Cole et al. 2014, which had a wider distribution of NCO events around the hotspot centre than we observed on average genome-wide. Given these results, and the fact that SDSA-based repair does generate heteroduplexes near the DSB breakpoint, we think gBGC does result primarily from repair of heteroduplexes formed by SDSA. We have modified Figure 6 for clarity.

L254: Do you mean Supplementary Figure 4b instead of 3b?

Yes, thank you. We have corrected it.

L254: Referring to Figure 4c: it would be more intuitive to categorize the changes into the following categories: S>W transitions, S>W transversions, W>S transitions and W>S transversion. This would allow the reader to better assess gBGC happening for W>S transitions and W>S transversion (W= weak AT and S = strong GC). Other changes do not lead to GC bias. Can you also please add the sample size of each category and the total number of events?
Why are S>S transversions underrepresented and W>W overrepresented in NCOs? Does this have to do with disruptive changes in the binding motif? Do you see differences if stratifying the data by PRDM9 allele?
Is there a reason for using "the relative proportion to the corresponding proportion of the nearest un-converted markers" instead of the relative proportion of all NCOs?

We initially separated mutational categories in an attempt to ease visual understanding, but in hindsight think the reviewers' suggestion is a good one. We have pooled strand-equivalent conversion types.

To the question of why S>S transversions are underrepresented relative to W>W transversions (though we note this difference is suggestive, but not statistically significant), this is exactly the feature that our two-mechanism framework aims to explain (illustrated in Figure 6 and Supplementary Fig. 8). This pattern can be explained if S alleles on the donor strand are restored (or equivalently "conversion is blocked") the majority of the time after heteroduplex formation, by a GC-biased mechanism that acts on single SNPs, when otherwise a strand-biased mechanism would almost always act to favour conversion to the donor allele. Please see the response to questions about L446-448 for more details. We show similar patterns when using a background model trained on nearby SNPs (Fig. 4c), or on SNPs within multi-SNP conversion tracts (Supplementary Fig. 5b). These approaches more faithfully represent the spectrum of SNPs encountered by NCO tracts (sampling SNPs close to the hotspot centre at a higher rate). Here we focus only on *Prdm9*^{Hum}-controlled events as they do not favour one background or the other on average across hotspots.

L265: The 500 bp in the Cole paper does not refer to GC bias, but to initiation bias between homologs. There are publications showing gBGC in CO like Lesecque et al. 2013, Ref 45 Arbeithuber 2015. In these datasets, GC-bias in CO was detected given on information of both reciprocals.

We have now added the correct reference in the text, citing Cole for the 500-bp estimate (line 368). Please see our response above regarding the question of gBGC at human COs.

L277: Here is a citation error. Exchange no 43 (Tiemann-Boege et al) against 45 (Arbeithuber et al. 2015). In ref 43 there is no data about gBGC in complex events.

Thank you for pointing out this error. We have corrected it in the main text (line 380).

L275-283: Can you rephrase this paragraph? I am not sure what the message is.
Please rephrase the statement "GC-biased process which normally only operates within single-SNP conversion tracts", since this is not completely established yet.

We have rephrased this section for clarity and removed this statement (lines 367-391).

L291-310: You claim that asymmetric binding is conserved between F2 and F5 animals-can you specify what data you use get to this conclusion? Please add information to the figure legend on the animals you are analyzing in Supp Fig 5a, F2 and F5s?). Do you have data for the symmetry analysis of F5s? Can you also add sample sizes?

We showed that asymmetric hotspots almost always contain a sequence variant in the PRDM9 motif (Supplementary Fig. 6f), indicating that asymmetry is a consequence of sequence variation that abolishes or weakens PRDM9 binding. The causal direction of this association is clear and well established (and we have also shown that variants outside the motif are not associated with binding symmetry). These same binding-altering sequence variants exist in both sexes and across generations, and they are expected to cause asymmetric PRDM9 binding in a similar manner in F5 mice and (though perhaps not always identically, at least typically in the same direction in) female mice. Prior work (Davies et al. 2016) also shows that hotspot symmetry and relative hotspot heats are highly correlated between different F1/F2 mice. In our Supplementary Fig 6c-e we also show a strong correlation between sex-averaged F5 event initiation and male F1 hotspot symmetry. We have added animals and sample sizes to the figure legends. Supplementary Fig. 6a shows DMC1 and H3K4me3 data from an F1 male, which does not involve CO/NCO events.

L312-315: We are confused of why you see asymmetric hotspots for PRDM9hum (Supp Fig5g). This seems to contradict your statement in L292-294 that the "PRDM9hum binds and initiates recombination equally well on both backgrounds". Can you verify that the asymmetric hotspots are dependent on PRDM9 binding? Specifically, that your proxy of H3K4me3 or DMC1 truly comes from PRDM9 activity, or that the asymmetry comes from differences in the hum motif (see also next comments).

By "PRDM9^{Hum} binds and initiates recombination equally well on both backgrounds", we mean when you sum across all *Prdm9^{Hum}*-controlled hotspots, the overall fraction of DMC1 or H3K4me3 reads from the B6 chromosome is similar to that of reads from the CAST chromosome (in contrast to the *Cast* allele, which primarily binds the B6 chromosome), but this is not necessarily true for individual hotspots. Sequence variants can still disrupt a PRDM9^{Hum} motif on one background but not the other at a particular site, yielding an asymmetric hotspot. However, in contrast to variants at binding sites for the *Cast* allele, these PRDM9^{Hum}-disrupting variants will not have been affected by historical recombination (resulting in mutations, gBGC, and hotspot drive). So, unlike PRDM9^{Cast} motif-disrupting variants, PRDM9^{Hum} motif-disrupting variants are equally likely to occur on the B6 and CAST backgrounds. We have clarified this in the text (lines 443-445). Please refer to Supplementary Figure 6a for more information. From this figure, you can see that most of the *Prdm9^{Hum}* allele's hotspots are symmetric but there are still some hotspots that are asymmetric; but notice that the total heat of the asymmetric hotspots that are initiated on B6 is similar to the total heat of asymmetric hotspots that are initiated on CAST. As mentioned above, we showed that asymmetric hotspots almost always contain a sequence variant in the PRDM9 motif (Supplementary Fig. 6f), confirming that differential PRDM9 binding is the cause of hotspot asymmetry.

L327: The next comments refer also to Figure 5 also for supplementary figure for F2 and F5. Could you please further subdivide CO and NCO into female and male CO or NCOs? There seems to be a lower number of DSB resolved as CO or NCO in male asymmetric hotspots (this is only the case for PRDM9hum in F2 but not F5, and I wonder why). Such a plot would be informative as to differences between males and females in the ratio of CO/NCO events resolved in asymmetric hotspots.

There might be a slight misunderstanding here. Figure 5 is about *Prdm9^{Hum}*-controlled F5 events only. We can only assign parental origin to F5 de novo events, so we cannot do the female and male analysis for F2 events or for F5 inherited events. For F5 de novo COs and NCOs, they behave near-identically as shown in Figure 5a, so we combined them for female and male analysis to increase sample size (note: the number of sex-resolved NCOs is very small: only 251 total, making the suggested analysis underpowered). Although from the plot it appears visually like there might be a lower number of DSBs resolved as CO or NCO in male asymmetric hotspots, this difference is not significant ($p=0.1$ from 1000 bootstrap samples).

How do the unknown and Prdm9 KO (6% of the hotspots) behave in terms of DSB and H3k4me3 events resolved into CO or NCO

As described in a response to another reviewer, it is very difficult to do the same analysis for "unknown" and *Prdm9* KO hotspots because we only observe 4 NCOs and 21 COs, in total, in these classes. There are a few reasons: Firstly, there are few events that overlap unknown and PRDM9 KO hotspots so we lack power. For example, for F5 de novo events, 5 of them overlap *Prdm9* KO hotspots and 25 of them overlap unknown hotspots. Secondly, to do the same analysis, we need to do rejection sampling, which requires PRDM9 binding motif position information. *Prdm9* KO hotspots do not have known binding motifs and it is difficult to determine what the binding motifs are for "unknown" hotspots.

There is only 1 NCO that overlaps a KO hotspot and 3 NCOs that overlap "unknown" hotspots; 6 COs overlap KO and 15 COs overlap "unknown" hotspots. For "unknown" hotspots, each could be *Prdm9^{Hum}* or *Prdm9^{Cast}* controlled, and so these are not necessarily PRDM9-independent events; even the 7 events overlapping KO hotspots might occur within PRDM9-dependent hotspots at coincident sites, and so there are not enough of these events to generate the same type of plot. What is clear is that there are very few, or no, events independent of PRDM9.

Are the hotspots you are plotting (Fig 5) verified to be PRDM9-dependent (see Fig 2d and e)? If so, in those few captured CO and NCO events in asymmetric hotspots; how far is the NCO or CO from the PRDM9 binding motif?

The hotspots we are plotting in Fig5 are *Prdm9*^{Hum}-controlled hotspots determined to be PRDM9-dependent (because they are not present in other animals, and most of them have a *Prdm9*^{Hum} binding motif). Since the way we sample events here requires motif information (please refer to Supplementary Note 8), all the events that are shown in Fig. 5 are near identified PRDM9 binding motifs (<1000 bp). We compared the distance-to-motif from CO and NCO events in asymmetric hotspots to all the CO and NCO events, and we did not detect a significant difference.

Please define "intermediate".

We ordered the hotspots by symmetry—either DMC1 (Figure 5b) or H3K4me3 (Figure 5a) symmetry—and binned the hotspots so each bin has the same number of expected events, either according to total DMC1 enrichment (Figure 5a) or total H3K4me3 enrichment (Figure 5b). Please refer to the responses to the next question about how we calculate the expected fraction (number) of events in each bin. By using 33% quantiles, we do not use a fixed symmetry threshold here to bin hotspots (but consistent boundaries for each plot are used), and the different measures of enrichment (DMC1 or H3K4me3) will have different exact cut-offs for "intermediate". That's why we used "intermediate" rather than a specific symmetry threshold to define the bin. Our motivation here is that there is no prior reason to expect the same threshold to be appropriate for both DMC1 and H3K4me3, given differences in repair timing, and background noise, for these features, so binning by quantiles seems a prudent thing to do.

L1007: Please elaborate how the DMC1-predicted fraction was estimated or refer to the appropriate SM Note or Methods and Materials.

We have added a reference to the supplementary note, as suggested. The DMC1 and H3K4me3 ChIP-seq enrichment at hotspots predict well how often recombination happens across all hotspots on average (Figure 2b-c and Supplementary Fig. 3f). So if we were to randomly separate hotspots into any three bins with equal total summed DMC1 or H3K4me3 enrichment in each bin, we would expect the same number of CO/NCO events to occur in each bin. However, we have uncovered previously hidden structure in the data: what we show in Figure 5 is that if you separate the three bins according to increasing hotspot symmetry while keeping the total DMC1/H3K4me3 enrichment the same in each bin, you no longer get an equal number of events in each bin.

L330: Please specify which Supp Note. Can you also specify the difference in the data between Fig 5a and Supp Fig 6a? For consistency purposes, can you make the same plot as Fig 5a for PRDM9Cst hotspots in Supp Fig 6?

Thanks and we specified Supplementary Note 8 in the main text (line 465). Fig 5a is for de novo events only and we also separate maternal and paternal events to make a comparison. It shows that de novo COs and NCOs have similar patterns and combined maternal and paternal events also show similar patterns. Supplementary Fig. 6a is for both de novo and inherited events. It shows that de novo and inherited events have similar patterns. We cannot make the same plot as Fig 5a for *Prdm9*^{Cast} hotspots, as we do not have de novo events that are controlled by *Prdm9*^{Cast} and cannot assign parental origin to *Prdm9*^{Cast}-controlled events either.

L332: "reflect chance genetic variation" is a difficult expression. Could you re-phrase it?

We have rephrased this sentence for clarity (lines 443-445):

> "Because the *Prdm9*^{Hum} allele in particular did not co-evolve alongside the mouse genome, asymmetric hotspots controlled by this allele arise from sequence variants that overlap and disrupt PRDM9^{Hum} binding sites on one homologue or the other by chance (i.e. not due to historical hotspot drive)."

L336-338: The binding of PRDM9 to a homolog is defined by the motif. If the motif is interrupted by an indel or a SNP, the binding affinity of PRDM9 changes. Can you remind the reader here again what proxies you are using to measure PRDM9 binding (H3k4me3 or SNPs in binding motif or both?) and which proxy for DSBs?

We have modified the text accordingly (lines 448-453):

> "Importantly, we found that this homologous recombination deficiency is driven by PRDM9 binding asymmetry alone (measured by haplotype-specific H3K4me3 enrichment), rather than SNP diversity elsewhere within hotspots (Supplementary Table 7). Furthermore, for DSBs occurring on the less-bound chromosome of asymmetric hotspots (measured by haplotype-specific DMC1 enrichment), we

found that NCO events occur at the expected rate for symmetric hotspots (Supplementary Fig. 7i-l and Supplementary Note 8).”

L338: You claim: “DSB occurring on the less bound chromosome of asymmetric hotspots” behave like symmetric hotspots in terms of NCO. How do CO behave in these cases? Do you have a plot or Figure showing this? It is very bizarre that the DSB happens at the chromosome not bound by PRDM9. How is this possible? Is something else introducing DSBs or is the high H3K4me3 not always a good proxy for PRDM9 binding.

We thank the reviewer for raising this point of confusion. Asymmetry is not a binary property; this can be explained e.g. if some sequence variants do not completely abolish PRDM9 binding but only weaken it. If a set of sites have 85% of H3K4me3 reads from the B6 background, that implies that PRDM9 still binds to the CAST background 15% of the time. The analysis of homologous heat described here and in Supplementary Note 8 would focus in on those 15%. Across many such sites, we find that NCOs arise from DSBs on the less-bound homolog in line with expectations from H3K4me3 enrichment specific to that homolog. But this would not be true for NCOs arising from DSBs on the more-bound homologue, where we see a strong depletion of NCOs. We show a similar pattern for COs in Supplementary Fig. 7e-h. As a result of this depletion, we see an elevation in the *fraction* of NCOs or COs initiating on the less-bound homolog compared to the expected fraction from H3K4me3 enrichment. We now illustrate this point in Figure 5c-d. We also see a depletion of DMC1 on the less-bound homologue relative to expectation from H3K4me3, consistent with the more-bound homologue taking longer to repair. Together these findings suggest that PRDM9 binding to the homolog is key for successful homologous recombination outcomes.

L345: H3K4me3 might not always reflect the level of PRDM9 binding, especially in non-B DNA regions with low H3K4me3 that might have a good PRDM9 binding due to the open chromatin structure. Note that symmetric open chromatin structure in both homologues might help repair DSB via NCO or CO. This was hypothesized in the context of methylation in Ref 43.

We agree that H3K4me3 is not a perfect measure of PRDM9 binding, but it does correlate well (see Altemose et al. 2017). Force-called H3K4me3 enrichment within DMC1 hotspots has also been shown to be a strong correlate of Spo11 oligo counts, which measure DSB frequency (r=0.83; Hinch et al. 2019). We show in this study that H3K4me3 correlates strongly (r=0.67) with DMC1 enrichment at individual hotspots, and hotspot symmetry estimates from the two measurements are very strongly correlated (r=0.88). Furthermore, we show that H3K4me3 enrichment predicts CO/NCO outcomes well (Figure 2b-c). Because we analyse a large number of sites across the entire genome to reach our conclusions, we do not expect any of the phenomena described by the reviewer to systematically bias our results.

L350: Inter-sister repair is quite plausible, but it still does not explain the lack of H3K4me3 signal upstream of the DSB formation.

We build a case in the following paragraph (line 489) in the text and in the Discussion (line 556) that the excess DMC1 signal relative to H3K4me3 signal at asymmetric sites can be explained by delayed DSB repair at these sites (see also Davies et al. 2016, Lange et al. 2016, and Hinch et al. 2019, which reach similar conclusions).

Discussion
To make it easier on the reader, could you add a heading or one or two sentences to recapitulate your main findings starting each major points.

We thank the reviewer for this suggestion, and we have added subheaders accordingly (e.g line 497).

L366-371: Please move this to the result section, since it is not fitting as your first paragraph in the discussion.

We have changed the ordering of the Discussion section to avoid this being at the very beginning. However, we prefer to keep this section in the Discussion, as it is highlights one of our major findings in the context of previous work then speculates, somewhat, about what may cause this difference in complex event frequency between mice and humans. Lines 498-515

Can you add a few details on how many complex NCO or complex CO you observed? In which animals, what hotspots, etc.?

We have added more information about complex recombination events to the Methods section “Identifying unique NCO and CO events” (lines 736-740). Of 1,575 observed NCO events, only 8 were “complex” and involved background switching within the event. Among these 8 NCOs, 2 of them are F5 de-novo NCOs and 6 of them are inherited NCOs detected from F5 mice. 7 of them overlap a hotspot. Of 1,116 observed de novo CO events from F2 and F5 animals, 7 were complex e.g. a CO accompanied by a NCO event. 6 of them are from F5 de novo events and 1 of them is from an F2 mouse. Among these 7 events, 6 of them overlap a hotspot.

L366: They also occur in human males, but they are probably more frequent in females. See Ref 19 Halldorsson et al.

We have clarified this in the text (line 507).

L367: grammar: nearly absent

Thanks and we have corrected it (line 508).

L370: The ability to repair heteroduplexes decreases with female age and was recently reported in BioRxiv. The finding of more complex events could be related to this lack of heteroduplex repair, and not necessarily only by non-programmed DSBs.

We think the reviewer is referring to this preprint https://www.biorxiv.org/content/biorxiv/early/2018/10/11/327098.full.pdf . In our Discussion we stated “These findings support the hypothesis that complex NCO events in humans might reflect the repair of non-programmed DNA damage occurring over time”. We believe the term “non-programmed DNA damage” (lines 512-513) encompasses the various mutational mechanisms described in the preprint, not just DSBs. We agree that repair might be compromised with age; because many of the complex human events occur outside PRDM9-controlled hotspots, it also suggests non-programmed DSB formation (or at least DNA damage) is also a factor.

L373: Is there a CpG bias in these complex NCO, where lesions are 5-meC dependent?

We detected very few complex events, so we cannot assess this with adequate power.

L376-378: The number of DSB resolved as a CO is also evolutionary constrained. Your data fits well the study of Segura et al. 2013. Proc. Biol. Sci. reporting a ratio of NCO/CO of 10:1 in mice versus 7:1 in primates. What controls this ratio is not fully understood, but factors like CO interference, chromosome packaging, and fundamental number of chromosomes play an important role. These points should be mentioned in the discussion.

After receiving correspondence from Dr Bjarni Halldorsson, we have removed speculation about the NCO:CO ratio in mice vs. humans because we now believe these ratios require further study. We cannot rule out the effects of long, complex gene conversions in increasing the overall human conversion rate per base (although it does seem clear that the human conversion rate per base is higher than that in mouse). (lines 504-505)

L393: use the word SNPs instead of mutations

We have corrected it (line 524).

L408: A depletion of CO+NCO events can also indicate a higher rate of inter-sister repair.

We agree. This is what we are trying to reason about in the next paragraph (lines 556-567). If the depletion of COs is not explained by genetic diversity alone, or DMC1 elevation alone, or an increase in NCO recombination, then inter-sister repair is the most appealing hypothesis that remains. It is an extremely difficult hypothesis to test though, given it leaves no genetic signature, so it remains a “diagnosis of exclusion” at present.

L413: Can you hypothesize how PRDM9 could assists with homologue search? Remember that PRDM9 is removed from the targeted homologue, once Spo11 cleaves the DNA exactly at the binding site of PRDM9.

We tried to simply remain cautious about ruling in/out different mechanisms here. It could be that PRDM9, in complex with other proteins, moves its binding sites to the SNC axis (as has been suggested by others) *before* DSB formation, so if it were to bind both homologues at a particular site, it would bring them into close

proximity prior to DSB formation and thereby reduce the physical search space of homology search if one of them gets a DSB. This could potentially happen during or after DSB formation as well. It could also be that homology search is limited only to regions marked by H3K4me3/H3K36me3, or that these chromatin marks remodel the local chromatin environment to make it more permissive to invading DNA strands, or some combination of the above explanations. We agree that it is not necessary for PRDM9 to be physically present for its action of binding to aid homology search.

L426: Can you reference a figure and/or other studies reporting this slower DSB repair in males? Could the higher overall methylation of DNA in males versus females during meiosis I play a role in this delay? See also reference 43 for a discussion on these sex differences in methylation during meiosis I.

We only include the parenthetical "(at least in males)" to highlight that our ChIP-seq data come only from testes and not fetal ovaries, not to highlight any difference between the sexes. We would expect to see elevated DMC1:H3K4me3 ratios at asymmetric hotspots in females as well, but we have not been able to examine this (and unfortunately the data from Brick et al. 2018 were not generated in hybrid female mice).

L437-439: Which of your data shows unequivocally that gBGC operates downstream of DSB? Can you add 1-2 sentences summarizing these findings? Could you also use the original terminology gBGC and not gcBGC

Please see above for a more detailed response. In brief, we show in Supplementary Table 6 and Supplementary Fig. 5a that gBGC occurs at single-SNP sites regardless of their DMC1 enrichment levels or hotspot symmetry. This implies that gBGC does not result from initiation biases in some way and so must result from a process downstream of DSB formation, in agreement with previous studies. We have corrected the gBGC terminology throughout the text and thank the reviewer for pointing this out.

L446-448: In your model, you are mixing DSB formation with DSB repair. You state that gBGC is the result of heteroduplex repair (L438) and occurs downstream of DSB formation (L438). Yet, in the first model explaining gBGC (Fig 6) you claim that this happens during DSB favoring one strand over the other. This does not make sense the way it is presented.
Also see Figure 6: Left panel: this seems DSB preference, known also as initiation bias or meiotic drive (see and cite Jeffreys work who described this first) explained now by the preferential PRDM9 binding.

We think there may be confusion about the possible model we have proposed, and we have edited the text for greater clarity in this respect (lines 586-641). We apologize for this confusion. We do not assume DSBs favour one strand over the other to explain gBGC—on the contrary, we have shown this is irrelevant for the single-SNP gBGC phenomenon we observe (Supplementary Table 6 and Supplementary Fig. 5a). When we say "block gene conversion" we mean blocking heteroduplex repair from selecting the donor strand's A/T allele when the recipient strand has a G/C allele--downstream of DSB formation, strand invasion/synthesis, and heteroduplex formation (as illustrated in Fig. 6). In other words, that site fails to gene convert and instead *restores* to the recipient homologue's allele. Because restored NCOs cannot be observed, we expect to the spectrum of observed events to be skewed, which is what we see in Figure 4c. From the results in this figure, we expect that we almost always observe conversion of a site when the recipient strand contains an A or T (regardless of whether the donor strand contains an A/T or G/C), but we observe conversion less frequently when the recipient strand contains a G or a C (regardless of whether the donor strand contains an A/T or G/C). Rather, our framework model suggests that there is a default "donor-biased" mechanism that favours the donor strand in most scenarios: in all multi-SNP tracts, in single-SNP tracts with an A/T on the recipient strand, and in ~47% of single-SNP tracts with G/C on the recipient strand. The remaining 53% of the latter case are repaired by a second, "GC-restoring" mechanism that always restores the site to the recipient's G/C allele. This GC-biased mechanism can very rarely also operate at individual SNPs within multi-SNP tracts, yielding complex events. Our new Supplementary Fig. 8 illustrates this possible model in greater detail, along with an alternative possible model.

NB: In Figure 6, we show the DSB occurring only on the blue chromosome in order to distinguish which chromosome is the donor or the recipient, not to imply that the hotspot here is asymmetric and has initiation bias. In this figure, we show three different outcomes of heteroduplex repair, depending on the identity of the mismatching bases (which for simplicity we assume do not disrupt PRDM9 binding). We also do not mean for the upper panel of Figure 6 to imply that SDSA is the *only* mechanism of NCO repair—it is only to illustrate which strand is the donor and which is the recipient.

L450: complex NCO or CO cannot be explained by an initiation bias; complex CO are likely the result of conversions during heteroduplex repair or template switching.

We agree. As described above for simple NCOs, we have not tried to imply that complex events are explained by initiation bias. When we say "block gene conversion" we mean restoring the recipient strand's G/C allele--downstream of DSB formation, strand invasion/synthesis, and heteroduplex formation (as illustrated in Fig. 6). In other words, in the case of a complex event containing a G/C allele on the recipient strand at one SNP, that site will fail to gene convert (i.e. it restores to the recipient homologue's allele after heteroduplex formation), even though nearby SNPs have converted to the donor homologue's allele.

L454: gBGC was also shown in COs not only in complex CO in ref 45.

We do not intend for the language to imply exclusivity; here we are only discussing complex events.

L459-467: This section is very confusing. Models of DSB repair using the unbroken homologue as a template are well established during strand invasion. Strand invasion is independent of MMR of heteroduplex repair acting downstream.

We thank the reviewer for raising this point of confusion. We have rewritten this section to improve clarity.

L478: BGC (misspelling)

Thanks, corrected (line 643).

Methods

L532-533: Add an additional sentence stating why it is important to remove potential hidden heterozygous sites in the F0 individuals?

We have added an additional sentence as suggested (line 702):

> "Heterozygous sites within the F0 individuals will mimic observed NCOs in the F2 mice."

L539: Please number your SM notes. So, it is much easier to find the correct one.

Thanks for this suggestion, now incorporated.

L541: refer to the specific SM about CO/NCO calling.

We now refer specifically to Supplementary Note 2 (line 712).

L544-546: What is the threshold? Add proportion of removed data.

The thresholds of all the filters are given in Supplementary Table 1. The proportion of removed potential NCOs (from the raw genotype calls in which genotyping errors initially predominate) is about 99.97%, and we have added it in the main text (line 720).

L548: Is this the total number of identified NCOs in F2 (including co-conversions)?

Yes, that refers to the total number of identified NCOs in F2 including co-conversions. Clusters of converted sites in the same animal are considered a single event and counted once.

L551-556: Can you add a sentence about the total number and proportion of inherited COs and NCOs versus de novo?

We have added the suggested sentence (lines 726-729):

> "We identified 821 de novo COs, 1384 inherited COs, 510 de novo NCOs, and 882 inherited NCOs; thus about 37% of the events are de novo for both COs and NCOs."

L559: what is the average tract length of these NCO events? Add also the length of your co-conversions? Do you see a difference in male vs female conversion tract lengths?

If we understand the question correctly, the reviewer is asking about the average tract length of *all* the NCO events. We estimated the tract length of *Prdm9*^{Cast}-controlled NCOs and *Prdm9*^{Hum}-controlled NCOs and showed in the main text (Figure 2h) that they differ. If we estimate the average tract length for all events combined, it is 36.2 bp (used for modelling in Supplementary Fig. 8). See the response to Reviewer 1 for more details on this procedure, which allows for uncertainty in the length of specific conversion tracts within the statistical analysis. By the same estimation method, the estimated paternal and maternal tract lengths are 43 bp and 35 bp respectively. These estimates are similar; we do not have evidence of statistically significant different tract lengths between the sexes (p=0.464). However we note that the numbers of sex-resolved NCOs are very small (121 and 130 paternal and maternal NCOs).

L560-561: How many CO and NCO events overlap with PRDM9 binding sites? (see also previous comments).

Among 4075 total COs and NCOs, 2517 of them overlap a PRDM9 binding motif (please also see previous responses).

L572-576: Why is there a decreasing CO/NCO overlap with increasing generation time?

This is expected because the DMC1 and H3K4me3 ChIP-seq data were gathered in testes from a male (B6xCAST)F1-*Prdm9*$^{Hum/Cast}$ mouse. These provide a measure of DSBs and PRDM9 binding happening in F1 meiotic cells, which directly yields the COs/NCOs detected in the genomes of F2 mice. Without competition with *Prdm9*^{Cast}, some weaker *Prdm9*^{Hum}-controlled hotspots, which are not detected in F1 ChIP-seq data, likely become stronger in the *Prdm9*$^{Hum/Hum}$ background of generations F2-F5. That would lead to a decreasing CO/NCO overlap in these later generations.

L579-599: Is there a different power for de novo vs. inherited tract length?

We estimated tract length for both de novo and inherited *Prdm9*^{Hum}-controlled NCOs and we did not detect a difference between the two. Per animal, we do not believe we expect different power to see short vs. long tracts for these different events – we might have slightly greater power to identify inherited events overall because they can be seen in multiple offspring, but we still must call such events in some individual offspring mouse for our pipeline to detect them.

L651-667: Is this motif caller accessible online?

Yes, it is accessible and has been published along with another paper from our group (Altemose et al. 2017, with the code available at: https://github.com/altemose/PRDM9-map).

L710-712: How many SNPs or indels came from symmetric or asymmetric hotspot? Is there a difference in SNP or indel variant density? Can you distinguish in your hotspot initiation biases? Do asymmetric HS repair differentiate from symmetric cases?

Yes, there is a difference in SNP or indel variant density between symmetric and asymmetric hotspots, especially within the motif (Supplementary Fig. 6f). Nearly all strongly asymmetric hotspots have SNPs or indels at the PRDM9 binding motif while only about 20% of the most symmetric hotspots possess SNPs or indels in the motif. In other words, the presence of a SNP or indel within the PRDM9 binding motif is a strong predictor of hotspot asymmetry. We also show that when COs/NCOs overlap asymmetric hotspots, their initiation bias is in the expected direction (although we also show they are less likely to occur at asymmetric hotspots overall—Fig. 5). We also show that asymmetric hotspots show signs of delayed repair (elevated DMC1 relative to H3K4me3, as described in Davies 2016 and Lange 2016) as well as signs of failed homologue-templated repair (depleted CO/NCO rates, shown in Fig. 5). In case the reviewer is asking if we compared SNPs *vs* indels, we did not detect enough indels to do any meaningful comparison of the two.

L716: PWD acronym is not explained.

Thanks, it is now explained in the text (line 906).

L736: In symmetric HS, do you observe a drift in homologous heat (e.g. DMC1)?

As we understand it, the reviewer is asking how much variation in symmetry there is around symmetric sites (e.g. sites with no mutations in the PRDM9 motifs). We have generated two plots to help address this. The left histogram shows the distribution of DMC1 symmetry estimates at hotspots with no detected mutations in the PRDM9 motif (*Prdm9*^{Hum} *and Prdm9*^{Cast}-controlled sites combined). The right histogram shows the distribution of DMC1 symmetry estimates at 642 hotspots with near-perfect 50% H3K4me3 symmetry estimates. Thus, some sites appear to be asymmetric even if they lack an identified variant in the motif, perhaps in part owing to epigenetic differences.

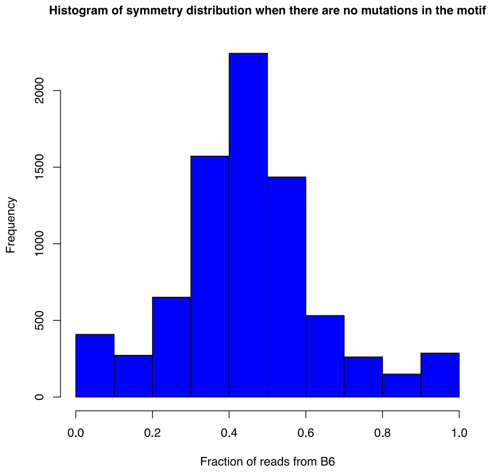

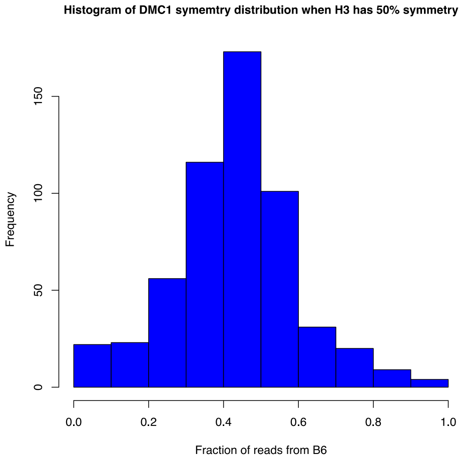

Supplementary Notes
L8: Please provide details how recombination events are classified into CO or NCO. For example, how do you differentiate a CO versus a NCO with a long conversion tract or a complex CO from a NCO?

Thanks for the suggestion, we have added more information to Supplementary Note 2.

L83: How many HS fall in this category?

Among the 23748 DMC1 hotspots, 2943 of them (12.4%) of them fall in “unknown” or “MULT” category.

Reviewer #3 (Remarks to the Author):

This is an impressive study that reports a large data set of meiotic recombination events in mouse, derived from sequencing recombinant populations. These data will be a valuable resource for the community. In addition the authors analyse the properties of these events and reveal some unexpected features of repair relating to mismatches. This is important, as the effects of heterozygosity/interhomolog polymorphism on meiotic recombination are relatively poorly understood. A further interesting dimension to these experiments is that the cross used contains two alleles of PRDM9 (the major protein driving mouse crossover locations), providing a means to test models of PDRM9 binding 'symmetry'.

We thank the reviewer for these kind comments and we do hope that our data and results will provide a useful resource for the meiotic recombination community.

The authors intercrossed two mouse subspecies (B6 with a human Prdm9 B allele sequence in the zinc finger array crossed with CAST) over 5 generations and sequenced 119 offspring (they sequenced 11 F2, 72 F5 and 36 F4 mice). These strains show a sequence divergence of ~0.7%. In total the authors identify ~1500 NCOs and ~2500 COs.
One interesting dimension is that polymorphism in PRDM9 binding sites causes differences in DSB hotspot activity. Previously shown that asymmetric binding associates with reduced fertility in hybrid mice. Asymmetric hotspots have greater DMC1 compared to H3K4me3 - consistent with longer repair. They also report that hotspots with high polymorphism and asymmetric binding, show stronger DMC1. Previous work in mammals has reported gene conversion events, which may be 1->1 kb and simple, or complex. Human NCO repair also show a ~68% GC bias - although as noted by the authors not all SNPs within a given hotspot show GC bias.

The authors compare CO and NCOs to H3K4me3 and DMC1 ChIP-seq and see a strong overlap, as expected. On page 8 line 177 the authors refer to '4,000 hottest hotspots' - it would be useful if the authors mentioned the total number of hotspots here, ie what proportion of the totalhotspots are these 4,000?

We have added the proportion in the main text (line 214):

> "…over 50% of all hotspot-associated F2 NCO or CO events occur in only the 4,000 hottest hotspots (ranked by DMC1 enrichment), around one sixth of the total number of hotspots."

Interestingly they see dominance of the Cast PRDM9 allele in terms of overlap with COs and NCOs. The authors explain this as being due to either differences in binding site strength, or a difference in expression level. The latter hypothesis should probably be tested using meiotic immunostaining.

We have included a citation (lines 218-221) of new evidence from qPCR that the total *Prdm9* expression level from both alleles does not change in heterozygotes relative to homozygotes, so it is unlikely that *Prdm9*^{Cast} is expressed at higher levels than *Prdm9*^{Hum}, although we still cannot formally rule out differences in actual protein levels. We also include a new hypothesis that dominance could be somewhat inflated due to PRDM9^{Hum} binding to promoters, where recombination does not occur.

Interestingly, also detect a slight difference in NCO tract length associated with each allele.

The NCO events were associated with GC bias (60-64%). As the humanized PRDM9 allele has not co-evolved with the cis sequences this provides a particularly interesting opportunity to investigate GC bias in a naïve/non-evolved situation. Only GC bias was observed for single SNP GCs, and not for multiple SNP, and interestingly this also related to local SNP desnity, with high SNPs associating with greater mismatches and no GC bias. For single site NCOs, versus the longer events, which differ in GC bias, do these groups differ in other respects - for example, overlap with gene or transposon annotations, or chromatin state (nucleosome occupancy might be interesting to see)?

We thank the reviewer for raising these points. We caution that we do not find evidence that the multi-SNP tracts are drawn from a distinct tract length distribution compared to the single-SNP tracts: we predict from our exponential tract length models that 20.5% of all observable tracts should contain multiple SNPs, and we observe 22.2%, with the slight increase likely owing in part to slightly increased power to detect multi-SNP events. Thus, it is unlikely that the length of multi-SNP tracts, when longer than average, is systematically related to their sequence or epigenetic contexts. We think the difference in GC-bias is attributable to the number

of SNPs in the heteroduplex, as opposed to the length or positioning of the heteroduplex (demonstrated by the analysis illustrated in Figure 4b). It's also unlikely that systematic variation in SNP density would yield the strength of effect that we observe. Given the high heterozygosity of these mice, NCO tracts can overlap multiple SNPs in all manner of different contexts across the genome.

Previous work is relevant to effects of local heterozygosity on CO/NCO rates - for example at budding yeast URA3 hotspot greater mismtaches increased NCOs at the expense of COs (Borts and Haber 1986), with the dominant model being that this is mediated via MSH2 MutS MMR anticrossover effects. The situation in mice is more complicated due to the activity of PRDM9, but I feel like these previous studies in yeast should be discussed.

We expect that NCOs in yeast repair by somewhat different mechanisms from mammals, given that they are much longer (~1 kb) and show no clear evidence of GC bias (Lesecque et al. 2013). Based on our model, we would speculate that the *observed* NCO:CO rate (after conditioning on power) should increase at hotspots with more local heterozygosity, similar to yeast, but only by virtue of invisible G/C 'restorations' at NCOs being less common at these sites (not that NCOs increase at the expense of COs).

I think it would be valuable to discuss the idea that heterozygosity may have an effect via formation of mismatches following interhomolog strand invasion also in the Introduction.

We have added this idea in the Introduction, as suggested (lines 76-80):

> "One study in mice (Smagulova et al. 2016) reported that hotspots with high polymorphism rates, particularly those with asymmetry, show a stronger DMC1 signal compared to observed numbers of overlapping crossovers. This raises the question of whether polymorphism itself can influence crossover outcomes; in yeast it has been shown that increased mismatches in heteroduplex recombination intermediates can suppress CO formation (Chambers et al. 1996)."

One general comment I have is that the data are analysed at fine-scale in detail, but I would value to see the recombination data plotted at larger scale along the chromosomes. For example, with a sliding window along the chromosomes how to NCO and CO frequency look? How do they relate to (i) historical recombination (ie LD based) estimates, (ii) AT:GC/isochore structure, (iii) gene density, (iv) polymorphism density, (v) heterochromatin eg H3K9me3, and (vi) alpha satellite density. Some of these patterns are mentioned in the text (page 9 lines 185-189) but it would be interesting to see them plotted.

We thank the reviewer for these suggestions. We have generated more broad-scale plots now shown in Supplementary Fig. 2 and noticed that NCOs, but not COs, increase in frequency near the centromere, and this effect is strongest on the shortest chromosomes. Interestingly, the telomere effect appears weaker for the *Prdm9*^{Cast} allele. We have also performed a Generalised Linear Model analysis to try to predict broad-scale CO and NCO rates for each allele given several other datasets and genome annotations: DMC1, H3K4me3, H3K9me3, H3K4me3 ChIP-seq Input Coverage (a measure of accessibility and 'sequenceability'), Mouse Satellite DNA Density, Gene Density, B6/CAST SNP number, GC content, distance from the centromere/telomere, and location in chromosomal compartment A vs B (crossover rate was shown to be higher in the gene-rich compartment A by Patel et al. 2018). We include the results of this analysis in Supplementary Table 3 and added a brief description to the main text (lines 170-181), with a full description of the method in Supplementary Note 4. GC content was found to be highly significantly predictive of broad-scale CO and NCO rates, and it seems to explain the overall correlation between recombination rates and location within chromatin compartment A. Interestingly, after controlling for GC content, location within compartment A becomes a *negative* predictor of recombination rate.

Minor points:

Line 85 - please explain why are gene conversions difficult to detect more clearly.

Thanks and we have explained more in the main text. NCO tracts are usually very short and if they do not cover any SNPs, there is no way to detect them by WGS sequencing (lines 87-89):

> "as NCOs are very short and the ability to detect them relies on the conversion of nearby SNPs, which is less likely to occur in individuals with low heterozygosity."

Lines 133-136. My reading of this is that you have just performed DSB analysis in males? How can you work out what is happening in female from these experiments?

We agree with the reviewer that it would be better to have DMC1 data from females but it is very difficult to generate. The first/only female DSB map was generated very recently, published in September 2018 (Brick et al. 2018). In female mice, meiotic DSBs form in the fetal ovary and each ovary contains approximately 100 times fewer meiocytes than such cells in adult testes. Thousands of females ovaries may be needed to generate a good DSB map. That study did not use hybrid mice or the same *Prdm9* alleles, so we cannot use their data to answer questions in our system. In our study, our CO/NCO events are from both females and males, and we see a very high overlap with recombination hotspots that are generated from males only. Because of this, we can conclude that there are few, if any, hotspots that are unique to females, consistent with findings from the recent ovary DSB map study (see below). This means, at least for hotspots, PRDM9 binds overwhelmingly to matching sites in males and females (though sometimes with different intensities), and implies that SNPs within PRDM9 binding motifs will almost certainly have similar impacts in both sexes. We therefore reason that key properties like hotspot symmetry are likely to be consistent between the sexes on average. According to our analysis, SNPs in the PRDM9 binding motif are the strongest predictors of hotspot symmetry, likely because they abolish or weaken PRDM9 binding. Because males and females share both these SNPs and PRDM9 binding sites, it is almost certain that at most sites, PRDM9 binding will be altered similarly in females and males, yielding similar hotspot symmetry measures.

The Brick et al. 2018 study concluded that there are few, if any, sex-specific DSB hotspots in mice:

> "Most DSB hotspots are found in both sexes (Extended Data Fig. 2a); 88% of hotspots from the better ovary DSB map are found in males, and this increases to 97% of hotspots common to both ovary maps. Hotspots unique to either sex are weak (Extended Data Fig. 2b–d) and contribute less than 2% of the SSDS signal. Given that strength estimates at weak hotspots are noisy and that ChIP–seq provides the relative rather than absolute estimates of hotspot use, it is likely that these hotspots are also used in the other sex, but with a frequency below our detection threshold." (Brick et al. 2018)

We now cite these findings in the main text (lines 405-408).

Lines 141. Please explain 'background changes' more clearly.

We have modified the main text to make it clearer, as suggested (lines 147-150). There are 3 background states: B6/B6, B6/CAST and CAST/CAST across the genome. We used an HMM algorithm as described in the manuscript (and Supplementary Note 2) to infer background states of each SNP. If the background changes between SNP one, which has a background B6/B6 and SNP two, which has a background B6/CAST, then we would identify a CO event between these two SNPs.

Line 159 - perhaps modify to 'telomeric enrichment' to make this clear?

Thanks, we have made this change.

Line 166 - ChIP-seq peaks of what?

Sorry, we should have made it clearer in the main text. This refers to either DMC1 or H3K4me3 ChIP-seq peaks. We have modified the main text accordingly (line 202).

References

Altemose, N., Noor, N., Bitoun, E., Tumian, A., Imbeault, M., Chapman, J. R., et al. (2017). A map of human PRDM9 binding provides evidence for novel behaviors of PRDM9 and other zinc-finger proteins in meiosis. *eLife*, *6*, e28383.

Arbeithuber, B., Betancourt, A. J., Ebner, T., & Tiemann-Boege, I. (2015). Crossovers are associated with mutation and biased gene conversion at recombination hotspots. *Proceedings of the National Academy of Sciences of the United States of America*, *112*(7), 2109–2114. http://doi.org/10.1073/pnas.1416622112

Baker, C. L., Walker, M., Kajita, S., Petkov, P. M., & Paigen, K. (2014). PRDM9 binding organizes hotspot nucleosomes and limits Holliday junction migration., *24*(5), 724–732. http://doi.org/10.1101/gr.170167.113

Berglund, J., Quilez, J., Arndt, P. F., & Webster, M. T. (2014). Germline methylation patterns determine the distribution of recombination events in the dog genome. *Genome Biology and Evolution*, *7*(2), 522–530. http://doi.org/10.1093/gbe/evu282

Billings, T., Parvanov, E. D., Baker, C. L., Walker, M., Paigen, K., & Petkov, P. M. (2013). DNA binding specificities of the long zinc finger recombination protein PRDM9. *Genome Biology*, *14*(4), R35–R35. http://doi.org/10.1186/gb-2013-14-4-r35

Brick, K., Thibault-Sennett, S., Smagulova, F., Lam, K.-W. G., Pu, Y., Pratto, F., et al. (2018). Extensive sex differences at the initiation of genetic recombination. *Nature*, *561*(7723), 338–342. http://doi.org/10.1038/s41586-018-0492-5

Chambers, S. R., Hunter, N., Louis, E. J., & Borts, R. H. (1996). The mismatch repair system reduces meiotic homeologous recombination and stimulates recombination-dependent chromosome loss. *Molecular and Cellular Biology*, *16*(11), 6110–6120. http://doi.org/10.1128/MCB.16.11.6110

Cole, F., Baudat, F. E. D. E. R., Grey, C., Keeney, S., de Massy, B., & Jasin, M. (2014). Mouse tetrad analysis provides insights into recombination mechanisms and hotspot evolutionary dynamics. *Nature Genetics*, 1–11. http://doi.org/10.1038/ng.3068

Crown, K. N., McMahan, S., & Sekelsky, J. (2014). Eliminating Both Canonical and Short-Patch Mismatch Repair in Drosophila melanogaster Suggests a New Meiotic Recombination Model. *PLoS Genetics*, *10*(9), e1004583. http://doi.org/10.1371/journal.pgen.1004583

Davies, B., Hatton, E., Altemose, N., Hussin, J. G., Pratto, F., Zhang, G., et al. (2016). Re-engineering the zinc fingers of PRDM9 reverses hybrid sterility in mice. *Nature*, 1–19. http://doi.org/10.1038/nature16931

Halldórsson, B. V., Hardarson, M. T., Kehr, B., Styrkarsdottir, U., Gylfason, A., Thorleifsson, G., et al. (2016). The rate of meiotic gene conversion varies by sex and age. *Nature Publishing Group*, *48*(11), 1377. http://doi.org/10.1038/ng.3669

Halldórsson, B. V., Palsson, G., Stefansson, O. A., Jonsson, H., Hardarson, M. T., Eggertsson, H. P., et al. (2019). Characterizing mutagenic effects of recombination through a sequence-level genetic map. *Science (New York, NY)*, *363*(6425), eaau1043–12. http://doi.org/10.1126/science.aau1043

Hinch, A. G., Zhang, G., Becker, P. W., Moralli, D., Hinch, R., Davies, B., et al. (2019). Factors influencing meiotic recombination revealed by whole-genome sequencing of single sperm. *Science (New York, NY)*, *363*(6433), eaau8861–12. http://doi.org/10.1126/science.aau8861

Lange, J., Yamada, S., Tischfield, S. E., Pan, J., Kim, S., Zhu, X., et al. (2016). The Landscape of Mouse Meiotic Double-Strand Break Formation, Processing, and Repair. *Cell*, *167*(3), 695–708.e16. http://doi.org/10.1016/j.cell.2016.09.035

Lesecque, Y., Mouchiroud, D., & Duret, L. (2013). GC-Biased Gene Conversion in Yeast Is Specifically Associated with Crossovers: Molecular Mechanisms and Evolutionary Significance. *Molecular Biology and Evolution*, *30*(6), 1409–1419. http://doi.org/10.1093/molbev/mst056

Liu, E. Y., Morgan, A. P., Chesler, E. J., Wang, W., Churchill, G. A., & de Villena, F. P.-M. (2014). High-Resolution Sex-Specific Linkage Maps of the Mouse Reveal Polarized Distribution of Crossovers in Male Germline. *Genetics*, *197*(1), 91–106. http://doi.org/10.1534/genetics.114.161653

Martini, E., Borde, V., Legendre, M., Audic, S., Regnault, B., Soubigou, G., et al. (2011). Genome-Wide Analysis of Heteroduplex DNA in Mismatch Repair–Deficient Yeast Cells Reveals Novel Properties of Meiotic Recombination Pathways. *PLoS Genetics*, *7*(9), e1002305. http://doi.org/10.1371/journal.pgen.1002305

Odenthal-Hesse, L., Berg, I. L., Veselis, A., Jeffreys, A. J., & May, C. A. (n.d.). Transmission Distortion Affecting Human Noncrossover but Not Crossover Recombination: A Hidden Source of Meiotic Drive. *PLoS Genetics*, *10*(2), e1004106. http://doi.org/10.1371/journal.pgen.1004106

Patel, L., Kang, R., Rosenberg, S. C., Qiu, Y., Raviram, R., Chee, S., et al. (2019). Dynamic reorganization of the genome shapes the recombination landscape in meiotic prophase. *Nature Structural & Molecular Biology*, *26*(3), 164. http://doi.org/10.1038/s41594-019-0187-0

Pratto, F., Brick, K., Khil, P., Smagulova, F., Petukhova, G. V., & Camerini-Otero, R. D. (2014). DNA recombination. Recombination initiation maps of individual human genomes. *Science (New York, NY)*, *346*(6211), 1256442. http://doi.org/10.1126/science.1256442

Smagulova, F., Brick, K., Pu, Y., Camerini-Otero, R. D., & Petukhova, G. V. (2016). The evolutionary turnover of recombination hot spots contributes to speciation in mice. *Genes & Development*, *30*(3), 266–280. http://doi.org/10.1101/gad.270009.115

REVIEWERS' COMMENTS:

Reviewer #1 (Remarks to the Author):

Li et al. use hybrid crosses to study the dynamics of recombination in mice. High divergence between these strains allows the authors to address several outstanding questions in the field which studies within species have been underpowered to resolve. I reviewed a previous version of this paper and feel that the authors took the comments from reviewers very seriously and have sufficiently addressed all of my concerns. I believe this paper will be extremely interesting to the broad audience that Nature Communications attracts. I have a few minor comments on this revised version:

Could the authors comment on recent results from Hinch et al. 2019 that suggest different conclusions about the stage at which asymmetric PRDM9 binding is important?

line 173 - lots of results from other species too (with and without PRDM9) so could be informative as to mechanism

line 181 - I am not familiar with this term "chromatin compartment A"

lines 224-232 - fascinating!

line 231 - is the expected effect size here consistent with the dominance of PRDM9cast?

line 585 - 640 - This is a very interesting model but I would recommend tempering the language a bit. i.e. The first pathway -> In our model the first pathway would

The remaining comments pertain to the supplemental notes:

Note 3: I appreciate the authors adding more information here but there are a large number of typos and it would be really helpful if this could be cleaned up and this section could be edited for clarity.

For example:
typo serious -> series
remove-> removing
insert size -> should be included?

What does step 14 refer to? There isn't consistency in numbering steps in this section

I found this language imprecise/confusing: "After applying the above filters, we removed sites if there were >2 sites filtered within 500bp, and the fraction of removed sites in this region (<500 bp) is >50%. This process was iterated until we don’t remove further sites (“guilt by association”). This aims to remove sites from bad regions."

Can the authors quantify this statement: "Because the chance of two recombination events occurring at the same location in only 11 animals is really small, we removed sites shared by 2 or more F2 animals, which may indicate that SNPs are miscalled, caused by mapping or other problems"

I found this sentence in Note 5 confusing: "It is interesting to consider the reverse: whether greater local SNP density itself could limit the length of gene conversion tracts by some mismatch detecting mechanism, or somehow related to the greater conversion rate we predict in regions with SNPs in close proximity."

Note 7: "identical in strength" seems like a potential overstatement of current evidence to me although estimates are strikingly similar. Perhaps indistinguishable?

Sincerely,

Molly Schumer

Reviewer #2 (Remarks to the Author):

I congratulate the authors for addressing so carefully all the points raised by the reviewers. The manuscript can be published in its present form. Indeed the MS reads much clearer and better now and will help the community to understand the importance and novelty of the observations/findings. Also the presentation of the model has observed quite an improvement and hopefully these ideas will be picked up by the community for further testing.

Reviewer #3 (Remarks to the Author):

I have read the revised manuscript and response letter from the authors. I am happy that the points I raised have been fully addressed.
Ian Henderson

Reviewer #1 (Remarks to the Author):

Li et al. use hybrid crosses to study the dynamics of recombination in mice. High divergence between these strains allows the authors to address several outstanding questions in the field which studies within species have been underpowered to resolve. I reviewed a previous version of this paper and feel that the authors took the comments from reviewers very seriously and have sufficiently addressed all of my concerns. I believe this paper will be extremely interesting to the broad audience that Nature Communications attracts. I have a few minor comments on this revised version:

Thank you for your very careful and helpful feedback on our work. We address your remaining concerns below.

Could the authors comment on recent results from Hinch et al. 2019 that suggest different conclusions about the stage at which asymmetric PRDM9 binding is important?

We have added comments to the discussion section to compare our findings:

> A recent crossover-mapping study, which sequenced single sperm from the same mouse cross examined here, similarly found CO depletion and DMC1 excess at asymmetric hotspots [Hinch et al. 2019]. Here we have gone further by demonstrating that both COs and NCOs are depleted at asymmetric sites, showing that many DSBs at asymmetric hotspots are not just delayed in their repair, but they often completely fail to ever repair from their homologue. Moreover, this occurs in female as well as male meiosis. This supports the hypothesis that homology search is the key process disrupted at asymmetric hotspots, rather than downstream events like CO versus NCO repair decisions.

line 173 - lots of results from other species too (with and without PRDM9) so could be informative as to mechanism

We have added a reference to dogs (Axelsson et al. 2012) as an example of PRDM9-lacking organisms that also show the telomere effect.

line 181 - I am not familiar with this term "chromatin compartment A"

This is one of two physically separated chromatin regions inferred from Hi-C data in mouse spermatocytes by Patel et al. 2019, and similar to compartments found in other cell types. All genomic regions can be assigned to one compartment or the other. Compartment A tends to be gene rich and GC rich, while compartment B is gene poor and GC poor. Patel et al. found that DSBs and crossovers are enriched in compartment A. Our results suggest that most of this effect is explained by the GC richness of chromatin compartment A. After conditioning on GC richness in a GLM analysis, regions in chromatin compartment A appear to be less likely to have CO or NCO recombination events.

lines 224-232 - fascinating!

We agree, total protein-DNA binding affinity (not just binding motif preferences) would appear to be yet another dimension affecting the evolution of PRDM9.

line 231 - is the expected effect size here consistent with the dominance of PRDM9cast?

The affinity of the humanized allele for promoters is predicted to contribute to *Prdm9*^{Cast} dominance, but it is not sufficient to explain the full effect. Using human PRDM9-B ChIP-seq peak data from our previous human cell line experiments (Altemose et al. 2017), if we sample between 1,000 and 40,000 peaks according to their enrichment values, we consistently observe around 10% of sampled peaks falling in promoters. Assuming similar binding properties within individual spermatocytes in the humanized mouse system, it's unlikely that more than 10% of actual humanized PRDM9 binding sites are unobserved due to their proximity to promoters. We have added this information to the main text.

line 585 - 640 - This is a very interesting model but I would recommend tempering the language a bit. i.e. The first pathway -> In our model the first pathway would

Thank you for the suggestion. We have modified the language considerably in this section to shorten it and to emphasize that our proposed model is a hypothesis.

The remaining comments pertain to the supplemental notes:

Note 3: I appreciate the authors adding more information here but there are a large number of typos and it would be really helpful if this could be cleaned up and this section could be edited for clarity.

For example:
typo serious -> series
remove-> removing
insert size -> should be included?

Thank you for pointing these out. We have corrected typographical errors in this section.

What does step 14 refer to? There isn't consistency in numbering steps in this section

We meant to refer to step 14 in the Supplementary Table 1. Now we have rephrased the whole paragraph to make it flow better.

I found this language imprecise/confusing: "After applying the above filters, we removed sites if there were >2 sites filtered within 500bp, and the fraction of removed sites in this region (<500 bp) is >50%. This process was iterated until we don't remove further sites ("guilt by association"). This aims to remove sites from bad regions."

We have edited these sentences for clarity:

> After applying the above filters, we removed sites within individuals if there were >2 sites filtered within 500 bp and if the fraction of removed sites in this region against all sites (<500 bp) is >50% ("guilt by association", aiming to identify and filter regions of poor genotyping quality). Because this further increases the number of removed sites, we iterated this process until it reached stationarity.

Can the authors quantify this statement: "Because the chance of two recombination events occurring at the same location in only 11 animals is really small, we removed sites shared by 2 or more F2 animals, which may indicate that SNPs are miscalled, caused by mapping or other problems"

By resampling simulated events according to their DMC1 enrichment, we estimate that this filter is likely to have removed only 3 true positives, while eliminating ~100,000 false positives. We have now noted this in the supplementary note.

I found this sentence in Note 5 confusing: "It is interesting to consider the reverse: whether greater local SNP density itself could limit the length of gene conversion tracts by some mismatch detecting mechanism, or somehow related to the greater conversion rate we predict in regions with SNPs in close proximity."

We have elaborated on this statement:

> While *inference* of mean tract length does not appear to depend on SNP density, we wondered if SNP density might affect *actual* tract lengths by some molecular mechanism. For example, perhaps high local SNP density can limit the length of gene conversion tracts by some mismatch detecting mechanism. Alternatively, because regions with lower SNP density are expected to show more overall gBGC (as illustrated in Figure 4b) and as a result are hypothesized to have a lower gene conversion rate due to the action of the 'GC-restoring' pathway (explained in Figure 6 and in Supplementary Note 7 below), then perhaps the longer observed tract lengths at the relatively SNP-poor $Prdm9^{Hum}$-controlled hotspots reflect the depletion of single-SNP G/C to A/T conversions. That is, because short tracts are more likely to contain single S/W SNPs that fail to convert, long tracts overlapping multiple SNPs are expected to become overrepresented, with a greater effect size at relatively SNP-poor $Prdm9^{Hum}$-controlled hotspots compared to SNP-rich $Prdm9^{Cast}$-controlled hotspots. This phenomenon would be expected to be amplified by the fact that $Prdm9^{Hum}$ binding sites are more G/C rich than $Prdm9^{Cast}$ binding sites. The magnitude of these effects would likely be modest, but they could contribute to the observed difference in tract lengths.

Note 7: "identical in strength" seems like a potential overstatement of current evidence to me although estimates are strikingly similar. Perhaps indistinguishable?

We agree with this point and have changed the language here.

Sincerely,

Molly Schumer

Reviewer #2 (Remarks to the Author):

I congratulate the authors for addressing so carefully all the points raised by the reviewers. The manuscript can be published in its present form. Indeed the MS reads much clearer and better now and will help the community to understand the importance and novelty of the observations/findings. Also the presentation of the model has observed quite an improvement and hopefully these ideas will be picked up by the community for further testing.

We thank the reviewer for their kind words, and we also hope our work can guide future investigations into the molecular mechanisms underlying our observations.

Reviewer #3 (Remarks to the Author):

I have read the revised manuscript and response letter from the authors. I am happy that the points I raised have been fully addressed.
Ian Henderson

Thank you again for your review of our work.
